# Selective sulfonylation and isonitrilation of *para*-quinone methides employing TosMIC as a source of sulfonyl group or isonitrile group

**DOI:** 10.3762/bjoc.17.193

**Published:** 2021-12-02

**Authors:** Chuanhua Qu, Run Huang, Yong Li, Tong Liu, Yuan Chen, Guiting Song

**Affiliations:** 1 College of Pharmacy, National & Local Joint Engineering Research Center of Targeted and Innovative Therapeutics, Chongqing Key Laboratory of Kinase Modulators as Innovative Medicine, Chongqing University of Arts and Sciences, Chongqing 402160, China

**Keywords:** chemoselective reactions, diarylmethyl sulfones, dual role, isonitrile diarylmethane, synthetic utility

## Abstract

Chemoselective sulfonylation and isonitrilation reactions for the divergent synthesis of valuable diarylmethyl sulfones and isonitrile diarylmethanes starting from easy-to-synthesize *para*-quinone methides (*p*-QMs) and commercially abundant *p*-toluenesulfonylmethyl isocyanide (TosMIC) by using respectively zinc iodide and 1,8-diazabicyclo[5.4.0]undec-7-ene (DBU) as catalysts were developed. The distinguishing feature of this method is that TosMIC plays a dual role from the same substrates in the reaction: as a sulfonyl source or as an isonitrile source. The synthetic utility of this protocol was also demonstrated in the synthesis of difluoroalkylated diarylmethane **5** and diarylmethane ketone derivatives **6** and **7**, which are important core structures in natural products and medicines.

## Introduction

**Figure 1:**
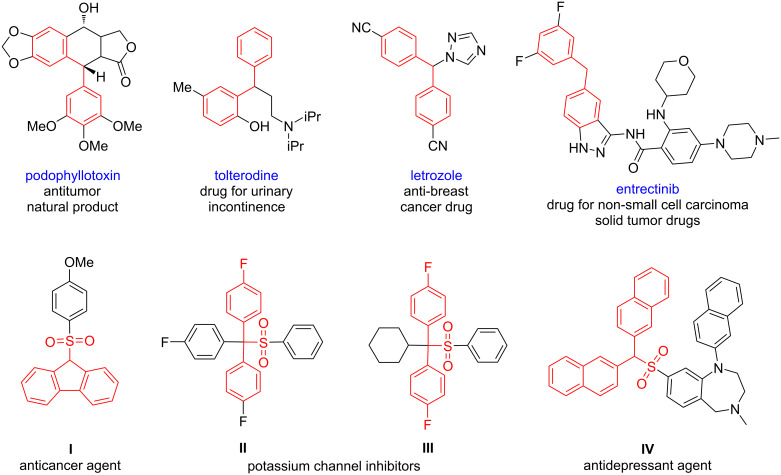
Selected bioactive compounds.

Sulfones are ubiquitous units commonly found in marketed drugs and natural products. Because of their unique electronic and structural properties, they are often used in medicinal chemistry programs to search for anti-inflammatory, anti-HIV, antimicrobial, antimalarial, and anticancer activities [1–2]. Diarylmethane motifs are widely present in natural products and pharmaceuticals that exhibit extraordinary biological activity [3–4] (Figure 1). Among them, their anticancer activity is particularly attractive, demonstrated by drugs such as letrozole [5] and entrectinib [6], with especially entrectinib showing a potent anticancer activity against a broad spectrum of human cancer cell lines. In recent decades, the construction of a hybrid system with varied biological and pharmaceutical activities has received extensive attention from medicinal chemists [7]. Therefore, merging the diarylmethane unit with the sulfonyl motif to assemble the sulfonyl diarylmethane skeleton has attractive potential application value and provides the possibility for drug discovery [8–10] (Figure 1). Consequently, the development of a rapid access to diarylmethyl sulfones is a valuable and appealing task in synthetic chemistry.

Traditionally, diarylmethyl sulfones are synthesized by transition-metal-catalyzed deoxy C–S bond-coupling reaction of sodium arylsulfinates with diarylmethanols [11], C–H functionalization of alkyl sulfones with aryl halides [12], and via a reductive strategy through nitrogen loss of sulfonyl hydrazones [13–14]. In addition, a sulfa-1,6-conjugated addition reaction [15–17] has also been developed for this purpose. Most of the reported methods are affected by long reaction times and the need for expensive metal catalysts or reagents. Therefore, there is great need to develop a more effective and rapid method for preparing diaryl methyl sulfones.

**Scheme 1:**
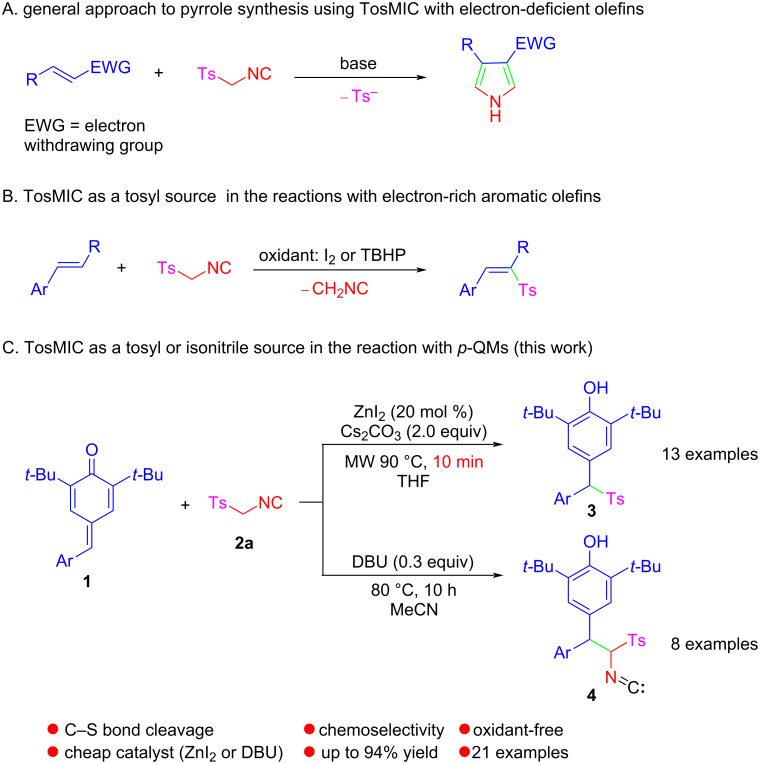
The chemistry of TosMIC in the reactions with olefins.

*p*-Toluenesulfonylmethyl isocyanide (TosMIC), a versatile synthon in organic chemistry, has been widely used to synthesize a myriad of valuable chemicals due to its high reactivity shown by the combination of acidic α-carbon atoms, isocyano groups, and sulfonyl moieties [18]. In general, TosMIC undergoes base-mediated 1,3-dipolar cycloadditions with activated alkenes to provide pyrroles as products [18] (Scheme 1A). Recently, alternative functionalizations using TosMIC as a tosyl source of arylalkenes or alkynes provided an attractive option for the synthesis of vinyl sulfones [19–23] (Scheme 1B). However, in contrast to the reaction of TosMIC as tosyl source with various aryl olefins, reports relating to reactions of TosMIC with electron-deficient olefins such as *p*-QMs for the preparation of highly valuable diarylmethyl sulfones are relatively scarce [24]. In addition, the important link between structural diversity and complexity with bioactivity represented by sulfones has led to the goal of developing strategies to as many these pivotal scaffolds as possible.

Isocyanide is an important C1 synthon. Its special reactivity, such as the ability to react with electrophilic, nucleophilic, and radical reagents [25–28], determines that it can participate in many types of reactions such as multicomponent reactions [29–32], tandem reactions [33–34], and insertion reactions [35–39], etc. In this context, the electron-rich aryl(phenol)methane isonitrile may be a new active unit, which can be seen from the previous case of *p*-QMs type reaction [40–51] and the above-mentioned properties of isocyanide.

Herein, we report the chemoselective sulfonylation and isonitrilation of *p*-QMs by changing the reaction conditions. This new general protocol allows, from the same substrates *p*-QMs **1** and *p*-tosylmethyl isocyanide (TosMIC, **2a**), the formation of quite different products **3** or **4** in a simple and mild manner, which provides an efficient entry into the rapid assembly of various diarylmethyl sulfones and isonitrile diarylmethanes (Scheme 1C).

## Results and Discussion

We commenced the study by using *p*-QMs **1a** and TosMIC (**2a**) as model substrates to optimize the reaction conditions. As shown in Table 1, when **1a** and **2a** were treated with 20 mol % Ag_2_CO_3_ in THF in the presence of Cs_2_CO_3_ at 90 °C under microwave irradiation for 10 min, fortunately, the sulfonylated diarylmethane product **3a** was isolated in 36% yield (Table 1, entry 2). When ZnI_2_ or Cu(OAc)_2_ was used instead of Ag_2_CO_3_, we found that the yield of **3a** catalyzed by ZnI_2_ reached 94% (Table 1, entry 3), and the reaction with Cu_2_(OAc)_2_ as a catalyst did not proceed smoothly (Table 1, entry 1). Other bases such as CH_3_ONa or *t*-BuONa (Table 1, entries 4 and 5) were then investigated and no better result was found. In the follow-up control experiments, we studied the reaction without adding bases, and unexpectedly found Ag salts could catalyze the 1,6-conjugate addition of TosMIC (**2a**) and *p*-QM **1a** to provide aryl(phenol)methane isonitrile **4a** under base-free conditions (Table 1, entries 6–8). When the silver salt was removed from the reaction conditions, the reaction did not proceed (Table 1, entry 9). Then, we investigated the effect of a catalytic amount of DBU on the reaction and found that reaction efficiency did not decrease, indicating that the reaction could also be catalyzed by DBU (Table 1, entry 10). When DABCO (triethylene diamine) was used instead of DBU, the reaction also proceeded, however, the reaction efficiency was lower compared to DBU (Table 1, entry 11).

**Table 1:** Optimization of the reaction conditions for the sulfonylation and isonitrilation of *p*-quinone methides with TosMIC.^a^

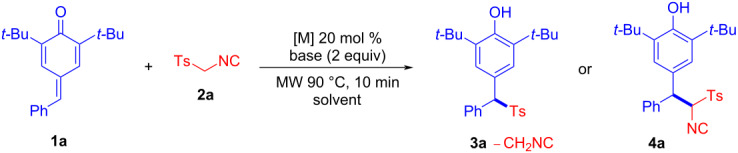					

Entry	[M] (20 mol %)	Solvent	Base	Yield of **3a** (%)^b^	Yield of **4a** (%)^b^

1	Cu(OAc)_2_	THF	Cs_2_CO_3_	trace	–
2	Ag_2_CO_3_	THF	Cs_2_CO_3_	36	–
**3**	**ZnI** **_2_**	**THF**	**Cs** **_2_** **CO** **_3_**	**94**	–
4	ZnI_2_	THF	CH_3_ONa	61	–
5	ZnI_2_	THF	*t-*BuONa	50	–
6^c^	Ag_2_CO_3_	MeCN	–	–	67 (dr = 1:1)
7^c^	Ag_2_O	MeCN	–	–	63 (dr = 1:1)
8^c^	AgOTs	MeCN	–	–	81 (dr = 1:1)
9^c^	–	MeCN	–	–	trace
**10** ^c,d^	–	**MeCN**	**DBU**	–	**82 (dr = 1:1)**
11^c,d^	–	MeCN	DABCO	–	61 (dr = 1:1)

^a^Reactions were performed on a 0.2 mmol scale of **1a** using 2.0 equiv of **2a**, 20 mol % [M], and 2.0 equiv of base, MW, 90 °C, 10 min; ^b^yields refer to the products isolated by column chromatography; ^c^80 °C oil bath for 10 h was used; ^d^0.3 equiv DBU or DABCO was used.

**Scheme 2:**
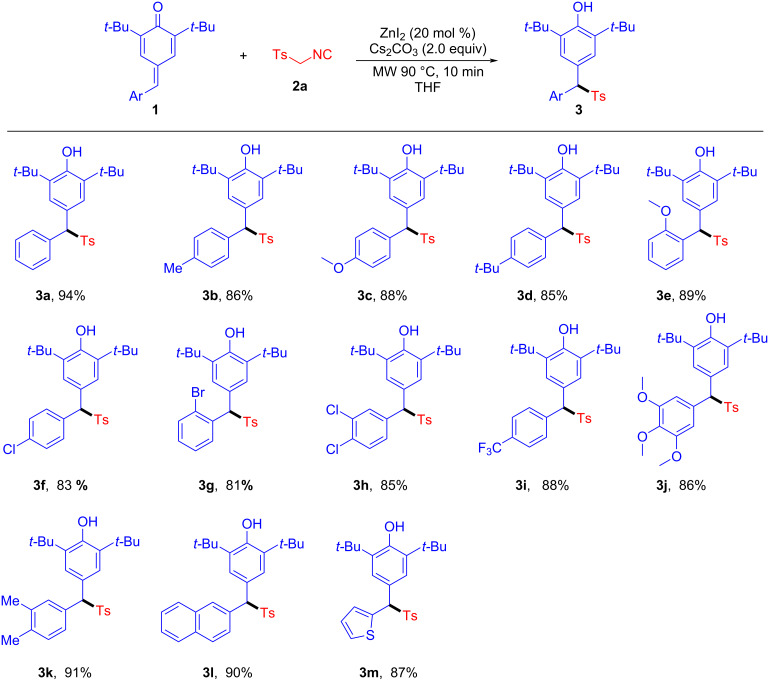
ZnI_2_-catalyzed C–S-bond cleavage of TosMIC for the synthesis of diarylmethyl sulfones **3a–m**. Reaction conditions unless otherwise specified: **1** (0.2 mmol), **2a** (0.4 mmol), ZnI_2_ (0.04 mmol), Cs_2_CO_3_ (0.4 mmol), THF (1.0 mL), MW 90 °C, under air atmosphere for 10 min; yields are reported for the isolated products.

After identifying the optimal conditions, we first evaluated the substrate scope of the sulfonylation reaction. As shown in Scheme 2, various substituted *p*-QMs were readily transformed in this sulfonylation reaction, providing the corresponding sulfonylated diarylmethane derivatives with good to excellent yields. Both electron-donating groups and electron-withdrawing substituents located in the *para*-, *ortho*-, or *meta*-position of the *p*-QMs were well tolerated and furnished the desired products **3a**–**m** in good yields (81–94% yields). It was noteworthy that *p*-QMs bearing functional groups, such as methyl, methoxy, *tert*-butyl, fluoro, chloro, bromo, and trifluoromethyl were well compatible under the optimal reaction conditions. The efficiency of this method was not affected by the pattern of substituents on the phenyl ring. In particular, *p*-QMs with naphthyl **1l** and thienyl moieties **1m** provided the products **3l** and **3m** in good yields (90 and 87%, respectively).

**Scheme 3:**
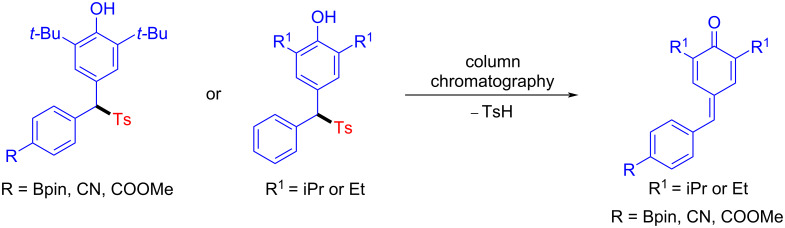
Cases encountered by other *p*-QMs examinations.

However, with other substituted *p*-QMs, such as Bpin, CN, and COOMe at C-4 position, and *p*-QMs bearing 2,6-diethyl or 2,6-diisopropyl, new main spots were detected by TLC, but they quickly decomposed during column chromatography so that the target products could not be obtained (Scheme 3), which might be due to the easy-to-cleave nature of the *p*-toluenesulfonyl group.

**Figure 2:**
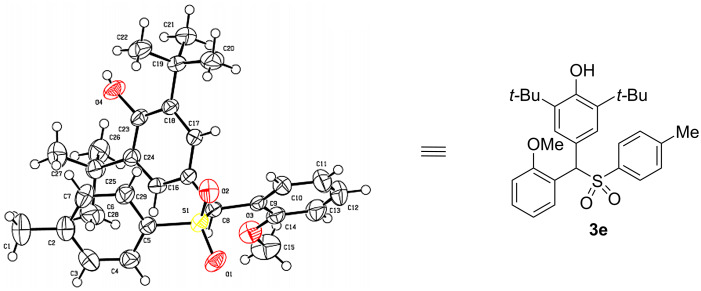
Crystal structure of diarylmethyl sulfone **3e**.

To further confirm the structure of the sulfonylated diarylmethanes, product **3e** was chosen as a representative compound and its structure was clearly verified by single crystal X-ray diffraction analysis, as shown in Figure 2 (CCDC No. 2104242).

**Scheme 4:**
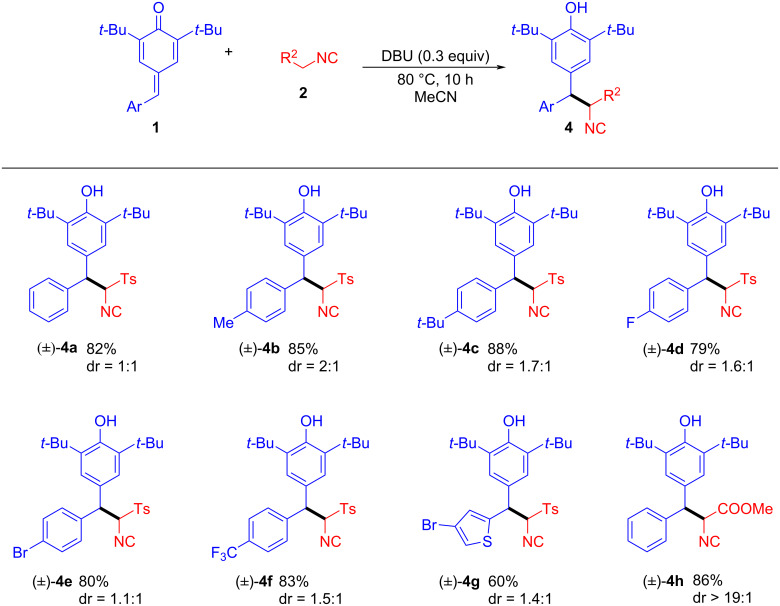
DBU-catalyzed 1,6-conjugate addition for the synthesis of isonitrile diarylmethanes **4a**–**h**. Reaction conditions unless otherwise specified: **1** (0.2 mmol), **2** (0.4 mmol), DBU (0.06 mmol), MeCN (1.0 mL), 80 °C, under air atmosphere for 10 h; yields are reported for the isolated products.

Next, the substrate scope of the 1,6-conjugate reaction of TosMIC to *p*-QMs was examined under optimized conditions (Table 1, entry 10). As depicted in Scheme 4, the substrate scope of *p*-QMs **1** was first examined. In general, the 1,6-conjugate reaction tolerated a wide range of *p*-QMs **1**, furnishing a series of isonitrile diarylmethanes **4a**–**h** in good to high yields (60–88%) with moderate diastereoselectivity. Substitution of the aryl ring of *p*-QMs **1** with functional groups such as alkyl, fluoro, bromo, trifluoromethyl, and thiophenyl was generally well tolerated (**4a**–**g**). Further, it should be noted that other activated methylene isonitriles such as methyl isocyanoacetate (**2b**) were also compatible with the reaction conditions providing, for example, the product **4h** with a yield of 86% and excellent diastereoselectivity (dr > 19:1).

**Scheme 5:**
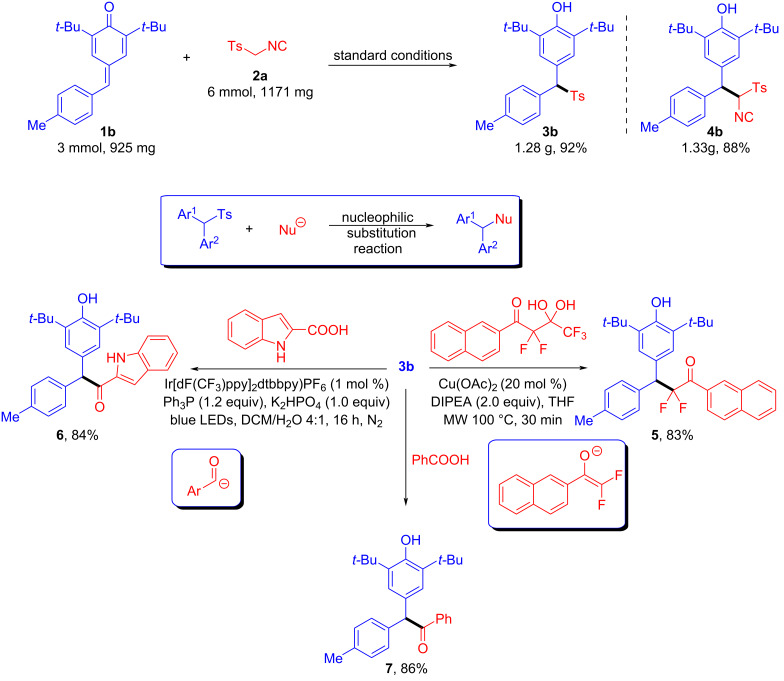
Synthetic applications of the synthesized compound **3b**.

To further underline the utility of this transformation, several experiments were carried out (Scheme 5). First, the model reaction is scalable. When **1b** (3 mmol) and **2a** (6 mmol) were mixed under the standard conditions, the desired products **3b** or **4b** were obtained with yields of 92% and 88%, respectively. Second, the sulfonylated diarylmethane **3b** obtained through the C–S bond-cleavage sulfonylation reaction is a versatile building block for preparing diarylmethane derivatives through a nucleophilic substitution process. For example, compound **3b** reacted with difluoroenolate to form the difluoroalkylated diarylmethane **5** in 83% yield via a Cu(OAc)_2_-catalyzed hydrodifluoroalkylation reaction [52]. Two other examples were the use of photoredox catalysis to generate acyl anions in situ from aromatic carboxylic acids via a triphenylphosphine-mediated deoxygenation process, followed by reaction with sulfonylated diarylmethane **3b** to obtain diarylmethane ketone derivatives **6** and **7** [53].

**Scheme 6:**
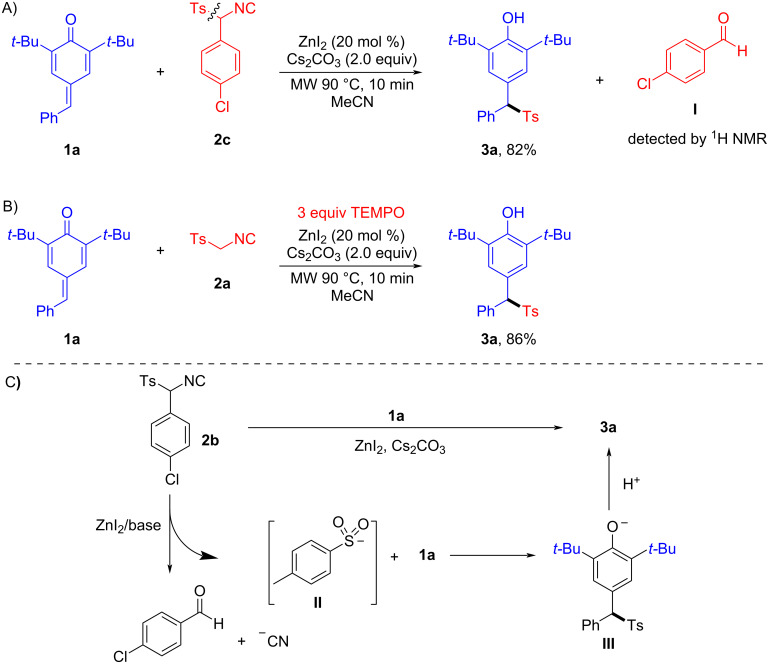
Mechanistic studies and proposed mechanism.

To gain mechanistic insight into this C–S-bond cleavage sulfonylation reaction, some control experiments were conducted (Scheme 6). The reaction of **1a** with TosMIC derivative **2c**, bearing an aromatic ring smoothly occurred to provide product **3a**; more importantly, the presence of *p*-chlorobenzaldehyde (**I**) released from **2c** can be detected by separation and ^1^H NMR analysis (Scheme 6A). This result indicates that TosMIC may decompose to a Ts anion and formaldehyde, possibly accompanied by the formation of a cyanide ion [54]. The previous reports on the reaction mechanism of TosMIC as a source of Ts are mainly a radical mechanism [19–23]. To assess the possibility of radical intermediates, a stoichiometric amount of the radical inhibitor 2,2,6,6-tetramethylpiperidine-1-oxyl (TEMPO) was subjected to the model reaction system, however, the reaction was not inhibited (Scheme 6B). This result suggests that no radical pathway is involved in this transformation. Based on the above experiments, a proposed mechanism is exemplified in Scheme 6C. The sulfonylation reaction starts with ZnI_2_/base system mediated C–S-bond cleavage of TosMIC derivative **2c** to yield Ts anion **II**, in which **2c** acts as the sulfonyl source. Finally, the sulfonylated diarylmethane **3a** is formed by a sequential addition/aromatization process.

## Conclusion

In conclusion, we have developed a chemoselective sulfonylation and isonitrilation of *p*-QMs by regulating the reaction conditions. This new general protocol allows completely different products to be formed from the same substrates in a simple and gentle manner, thereby efficiently and quickly assembling various diaryl methyl sulfones and isonitrile diarylmethanes. In the follow-up study, it was found that the isonitrile diarylmethanes are versatile building blocks for the rapid assembly of a series of compounds with novel structures. We are studying this part of the work.

## Experimental

General reaction procedure for synthesis of diarylmethyl sulfones **3**: In an oven-dried glass tube *p*-QM **1** (0.2 mmol, 1.0 equiv), TosMIC (*p*-toluenesulfonyl isonitrile (**2a**, 0.4 mmol, 2.0 equiv), Cs_2_CO_3_ (0.4 mmol, 2.0 equiv), and ZnI_2_ (0.04 mmol, 0.2 equiv) were dissolved in THF (1 mL). The glass tube was sealed and the reaction mixture was heated under microwave irradiation at 90 °C for 10 min and monitored by TLC until starting material was consumed. Then, the reaction mixture was concentrated under reduced pressure followed by column chromatography over silica gel using petroleum/EtOAc (0 to 10%) as eluent to afford the desired product **3**.

General reaction procedure for synthesis of isonitrile diarylmethane **4**: In an oven-dried glass tube *p*-QM **1** (0.2 mmol, 1.0 equiv), TosMIC (*p*-toluenesulfonyl isonitrile (**2a**, 0.4 mmol, 2.0 equiv), DBU (9 µL, 0.06 mmol, 0.3 equiv) were dissolved in MeCN (1 mL) and the reaction mixture was stirred at 80 °C for 10 h and monitored by TLC. Then, the reaction mixture was concentrated under reduced pressure followed by column chromatography over silica gel using petroleum/EtOAc 10:1 to ≈5:1 as eluent to afford the desired product **4**.

## Supporting Information

File 1 General information, characterization data, and copies of ^1^H and ^13^C NMR spectra.
