# Supplementary material for: Selective sulfonylation and isonitrilation of para-quinone methides employing TosMIC as a source of sulfonyl group or isonitrile group
Source: Beilstein J Org Chem. 2021 Dec 2;17:2822–31. doi: 10.3762/bjoc.17.193 (PMC8649203; doi:10.3762/bjoc.17.193)

BEILSTEIN JOURNAL OF ORGANIC CHEMISTRY

Supporting Information

for

Selective sulfonylation and isonitrilation of *para*-quinone methides employing TosMIC as a source of sulfonyl group or isonitrile group

Chuanhua Qu, Run Huang, Yong Li, Tong Liu, Yuan Chen and Guiting Song

Beilstein J. Org. Chem. **2021,** *17,* 2822–2831. doi:10.3762/bjoc.17.193

General information, characterization data, and copies of ^{1}H and ^{13}C NMR spectra

License and Terms: This is a supporting information file under the terms of the Creative Commons Attribution License (https://creativecommons.org/licenses/by/4.0). Please note that the reuse, redistribution and reproduction in particular requires that the author(s) and source are credited and that individual graphics may be subject to special legal provisions.
The license is subject to the *Beilstein Journal of Organic Chemistry* terms and conditions: (https://www.beilstein-journals.org/bjoc/terms)

Table of contents

1. General information

2. Microwave irradiation experiments

3. Preparation methods and physical data of products

4. References

5. Copies ^{1}H NMR, ^{13}C NMR, and ^{19}F NMR spectra

General information

^{1}H NMR, ^{19}F NMR and ^{13}C NMR spectra were measured on a 400 MHz spectrometer, using $CDCl_3$ or DMSO-d_6 as the solvent with tetramethylsilane (TMS) as the internal standard at room temperature. Chemical shifts (δ) are given in ppm relative to TMS, the coupling constants *J* are given in Hz. HRMS were obtained in the ESI mode. All reactions were carried out under air atmosphere unless otherwise noted. All solvents were obtained from commercial suppliers. Reactions were monitored by thin-layer chromatography (TLC) on silica gel plates (GF254), and analytical TLC was performed on precoated, glass-backed silica gel plates. The products were purified by Biotage Isolera™ Spektra Systems and petroleum ether/EtOAc solvent systems. All reagents and solvents were obtained from commercial sources and used without further purification. *p*-QMs **1** were prepared according to the literature [1].

Microwave irradiation experiments

All microwave irradiation experiments were carried out in a Biotage® Initiator Classic microwave apparatus with continuous irradiation power from 0 to 400 W with utilization of the standard absorbance level of 250 W maximum power. The reactions were carried out in 10 mL glass tubes, sealed with microwave cavity. The reaction was irradiated at a required ceiling temperature using maximum power for the stipulated time. Then it was cooled to 50 °C with gas jet cooling.

Gram-scale reaction to synthesis of diarylmethyl sulfone 3b

To an oven-dried glass tube (25 mL) equipped with a magnetic stirring bar, **1b** (3 mmol, 0.925 g), **2a** (2.0 equiv, 6 mmol, 1.171 g), Cs_2CO_3 (2.0 equiv, 6 mmol, 1.955 g), ZnI_2 (0.2 equiv, 0.6 mmol, 191 mg), and 15 mL THF (syringe) were added. The tube was placed in a Biotage® Initiator Classic microwave apparatus, and the resulting solution was stirred under microwave irradiation at 90 °C for 10 min and monitored by TLC. After the reaction was finished, the mixture was concentrated under vacuum to remove THF, and the residue was purified by chromatography on silica gel (EA/PE 1:10) to afford 1.28 g of product **3b** (92% yield) as colorless oil.

Gram-scale reaction to synthesis of isonitrile diarylmethane 4b

To an oven-dried glass tube (25 mL) equipped with a magnetic stirring bar, **1b** (3 mmol, 0.925 g), **2a** (2.0 equiv, 6 mmol, 1.171 g), DBU (0.3 equiv, 0.9 mmol, 135 μL), and 15 mL MeCN (syringe) were added and the resulting solution was stirred at 80 °C for 10 h and monitored by TLC. After the reaction was finished, the mixture was concentrated under vacuum to remove MeCN, and the residue was purified by chromatography on silica gel (EA/PE 1:10) to afford 1.33 gof product **4b** (88% yield) as yellow solid.

General reaction procedure for the synthesis of difluorinated diarylmethane 5 in a manner analogous to [2].

In an oven-dried glass tube, **3b** (0.2 mmol, 93 mg, 1.0 equiv), α-difluorinated *gem*-diols (0.3 mmol, 96 mg, 1.5 equiv), DIPEA (0.4 mmol, 70 μL, 2.0 equiv), $Cu(OAc)_2$ (0.04 mmol, 8 mg, 0.2 equiv) were dissolved in THF (2 mL) and the reaction mixture was sealed and heated under microwave irradiation at 100 °C for 30 min and monitored by TLC until starting material was consumed. Then, the reaction mixture was concentrated under reduced pressure followed by column chromatography over silica gel using petroleum/EtOAc (0 to 5%) as eluent to afford the desired product **5**, 85 mg, 83% yield. Compound **5** is known [2].

General reaction procedure for the synthesis of diarylmethane 6

Diarylmethyl sulfone **3b** (0.2 mmol, 93 mg, 1.0 equiv) was added to a mixture of K_2HPO_4 (0.2 mmol, 35 mg, 1 equiv), 1*H*-indole-2-carboxylic acid (0.3 mmol, 48 mg, 1.5 equiv), PPh_3 (0.24 mmol, 63 mg, 1.2 equiv) and $Ir[dF(CF_3)ppy]_2dtbbpy)PF_6$ (0.002 mmol, 2.2 mg, 0.01 equiv) with DCM (1.6 mL)/H_2O (0.4 mL) in a 10 mL glass vial equipped with a magnetic stirring bar and a nitrogen inlet. The mixture was degassed by three cycles of freeze–pump thaw and then placed in the irradiation apparatus equipped with a 24 W blue light emitting diode (LED) strip. The resulting mixture was stirred at room temperature for 16 h. The crude product was purified by column chromatography (silica gel, EtOAc/*n*-hexane 1:10) to afford the desired product **6** (76mg, 84% yield).

General reaction procedure for the synthesis of diarylmethane 7

Diarylmethyl sulfone **3b** (0.2 mmol, 93 mg, 1.0 equiv) was added to a mixture of K_2HPO_4 (0.2 mmol, 35 mg, 1 equiv), benzoic acid (0.3 mmol, 37 mg, 1.5 equiv), PPh_3 (0.24 mmol, 63 mg, 1.2 equiv) and Ir[dF(CF_3)ppy]$_2$dtbbpy)PF_6 (0.002 mmol, 2.2 mg, 0.01 equiv) with DCM (1.6 mL)/H_2O (0.4 mL) in the 10 mL glass vial equipped with a magnetic stirring bar and a nitrogen inlet. The mixture was degassed by three cycles of freeze–pump thaw and then placed in the irradiation apparatus equipped with a 24 W blue light emitting diode (LED) strip. The resulting mixture was stirred at room temperature for 16 h. The crude product was purified by column chromatography (silica gel, EtOAc/*n*-hexane 1:10) to afford the desired product **7** (71 mg, 86% yield).

2,6-Di-*tert*-butyl-4-(phenyl(tosyl)methyl)phenol (**3a**) [3]

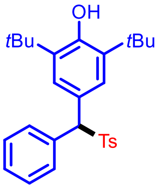

94% yield; ^{1}H NMR (400 MHz, $CDCl_3$) δ 7.55 (dd, *J* = 7.8, 1.5 Hz, 2H), 7.35 (d, *J* = 8.2 Hz, 2H), 7.26 (d, *J* = 7.8 Hz, 2H), 7.09 (s, 2H), 7.06 (d, *J* = 8.1 Hz, 2H), 5.15 (s, 1H), 5.10 (s, 1H), 2.29 (s, 3H), 1.28 (s, 18H); ^{13}C NMR (100 MHz, $CDCl_3$) δ 154.1, 144.0, 135.8, 135.6, 133.6, 130.0, 129.1 (d, *J* = 1.7 Hz), 128.7, 128.4, 127.1, 123.4, 76.8, 34.3, 30.1, 21.5. HRMS: m/z calcd for $C_{28}H_{35}O_3S^+$ $(M+H)^+$ 451.2301, found *m/z* 451.2309.

2,6-Di-*tert*-butyl-4-(*p*-tolyl(tosyl)methyl)phenol (**3b**) [3]

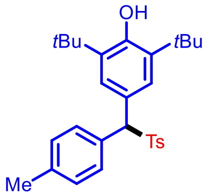

86% yield; ^{1}H NMR (400 MHz, $CDCl_3$) δ 7.51 (d, *J* = 8.1 Hz, 2H), 7.42 (d, *J* = 8.2 Hz, 2H), 7.18 – 7.11 (m, 6H), 5.20 (s, 1H), 5.13 (s, 1H), 2.36 (s, 3H), 2.33 (s, 3H), 1.35 (s, 18H); ^{13}C NMR (100 MHz, $CDCl_3$) δ 154.0, 143.9, 138.3, 135.8, 135.7, 130.4, 129.8, 129.4, 129.1 (d, *J* = 5.2 Hz), 127.0, 123.6, 76.6, 34.3, 30.1, 21.5, 21.2. HRMS: m/z calcd for $C_{29}H_{37}O_3S^+$ $(M+H)^+$ 465.2458, found *m/z* 465.2452.

2,6-Di-*tert*-butyl-4-((4-methoxyphenyl)(tosyl)methyl)phenol (**3c**) [3]

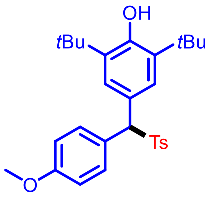

88% yield; ^{1}H NMR (400 MHz, $CDCl_3$) δ 7.46 (d, *J* = 8.6 Hz, 2H), 7.35 (d, *J* = 8.2 Hz, 2H), 7.09 – 7.03 (m, 4H), 6.80 (d, *J* = 8.8 Hz,

2H), 5.14 (d, J = 1.2 Hz, 1H), 5.06 (s, 1H), 3.72 (s, 3H), 2.29 (s, 3H), 1.28 (s, 18H); ^{13}C NMR (100 MHz, $CDCl_3$) δ 159.7, 154.0, 143.9, 135.8, 135.7, 131.2, 129.1, 127.0, 125.4, 123.7, 114.1, 76.2, 55.3, 34.3, 30.1, 21.5. HRMS: m/z calcd for $C_{29}H_{37}O_4S^+$ $(M+H)^+$ 481.2407, found *m/z* 481.2411.

2,6-Di-*tert*-butyl-4-((4-(*tert*-butyl)phenyl)(tosyl)methyl)phenol (**3d**)

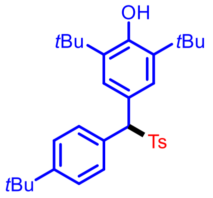

85% yield; ^{1}H NMR (400 MHz, $CDCl_3$) δ 7.49 (d, J = 8.0 Hz, 2H), 7.36 – 7.25 (m, 4H), 7.07 (s, 2H), 7.04 (d, J = 7.1 Hz, 2H), 5.12 (s, 1H), 5.06 (s, 1H), 2.28 (s, 3H), 1.27 (s, 18H), 1.23 (s, 9H); ^{13}C NMR (100 MHz, $CDCl_3$) δ 154.0, 151.4, 143.9, 135.8, 135.6, 130.2, 129.7, 129.0 (d, J = 12.6 Hz), 127.1, 125.7, 123.7, 76.7, 34.6, 34.3, 31.3, 30.1, 21.5. HRMS: m/z calcd for $C_{32}H_{43}O_3S^+$ $(M+H)^+$ 507.2927, found *m/z* 507.2919.

2,6-Di-*tert*-butyl-4-((2-methoxyphenyl)(tosyl)methyl)phenol (**3e**) [3]

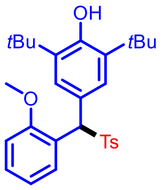

89% yield;^{1}H NMR (400 MHz, $CDCl_3$) δ 8.14 (dd, J = 7.8, 1.6 Hz, 1H), 7.44 (d, J = 8.2 Hz, 2H), 7.28 – 7.26 (m, 1H), 7.22 (s, 2H), 7.13 (d, J = 8.1 Hz, 2H), 7.07 – 7.02 (m, 1H), 6.73 (d, J = 7.9 Hz, 1H), 5.93 (s, 1H), 5.20 (s, 1H), 3.60 (s, 3H), 2.36 (s, 3H), 1.36 (s, 18H); ^{13}C NMR (100 MHz, $CDCl_3$) δ 156.9, 154.0, 143.7, 136.1, 135.6, 129.8, 129.4, 129.1, 128.9, 127.4, 123.4, 122.4, 120.7, 110.7, 66.9, 55.5, 34.3, 30.1, 21.5. HRMS: m/z calcd for $C_{29}H_{37}O_4S^+$ $(M+H)^+$ 481.2407, found *m/z* 481.2413.

2,6-Di-*tert*-butyl-4-((4-chlorophenyl)(tosyl)methyl)phenol (**3f**) [3]

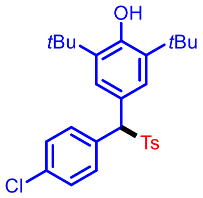

83% yield; ^{1}H NMR (400 MHz, $CDCl_3$) δ 7.50 (d, J = 8.4 Hz, 2H), 7.34 (d, J = 8.1 Hz, 2H), 7.25 (d, J = 8.4 Hz, 2H), 7.07 (d, J = 8.1 Hz, 2H), 7.02 (s, 2H), 5.18 (s, 1H), 5.08 (s, 1H), 2.28 (s, 3H), 1.27 (s, 18H) ppm; ^{13}C NMR (100 MHz, $CDCl_3$) δ 154.2, 144.3, 136.0, 135.3, 134.5, 132.1, 131.3, 129.2, 129.1, 128.9, 127.0, 123.0, 76.0, 34.3, 30.1, 21.6 ppm; HRMS: m/z calcd for $C_{28}H_{34}ClO_3S^+$ $(M+H)^+$ 485.1912, found *m/z* 485.1915.

4-((2-Bromophenyl)(tosyl)methyl)-2,6-di-*tert*-butylphenol (3g) [3]

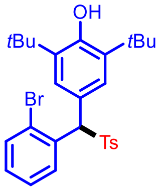

81% yield; ^{1}H NMR (400 MHz, $CDCl_3$) δ 8.40 (d, *J* = 7.5 Hz, 1H), 7.48 (dd, *J* = 20.1, 7.8 Hz, 4H), 7.28–7.06 (m, 5H), 5.94 (s, 1H), 5.29 (s, 1H), 2.40 (s, 3H), 1.39 (s, 18H) ppm; ^{13}C NMR (101 MHz, $CDCl_3$) δ 154.2, 144.4, 135.9, 135.6, 133.8, 133.2, 130.1, 129.7, 129.2, 129.0, 127.8, 127.2, 126.0, 122.4, 73.9, 34.3, 30.1, 21.6 ppm; HRMS: m/z calcd for $C_{28}H_{34}BrO_3S^+$ $(M+H)^+$ 529.1407, found *m/z* 529.1403.

2,6-Di-*tert*-butyl-4-((3,4-dichlorophenyl)(tosyl)methyl)phenol (3h)

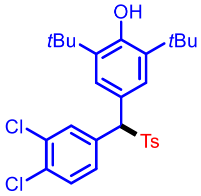

85% yield; ^{1}H NMR (400 MHz, $CDCl_3$) δ 7.67 (d, *J* = 2.1 Hz, 1H), 7.57 (dd, *J* = 8.4, 2.2 Hz, 1H), 7.43 (dd, *J* = 11.3, 4.9 Hz, 3H), 7.16 (d, *J* = 8.0 Hz, 2H), 7.05 (s, 2H), 5.27 (s, 1H), 5.11 (s, 1H), 2.38 (s, 3H), 1.34 (s, 18H); ^{13}C NMR (100 MHz, $CDCl_3$) δ 154.3, 144.6, 136.1, 135.0, 133.7, 132.8 (d, *J* = 5.2 Hz), 132.0, 130.5, 129.3, 129.1, 129.0, 126.9, 122.5, 75.5, 34.3, 30.1, 21.6. HRMS: m/z calcd for $C_{28}H_{33}Cl_2O_3S^+$ $(M+H)^+$ 519.1522, found *m/z* 519.1531.

2,6-Di-*tert*-butyl-4-(tosyl(4-(trifluoromethyl)phenyl)methyl)phenol (3i) [3]

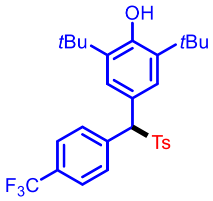

88% yield; ^{1}H NMR (400 MHz, $CDCl_3$) δ 7.79 (d, *J* = 8.1 Hz, 2H), 7.62 (d, *J* = 8.2 Hz, 2H), 7.42 (d, *J* = 8.1 Hz, 2H), 7.15 (d, *J* = 8.0 Hz, 2H), 7.09 (s, 2H), 5.26 (s, 1H), 5.22 (s, 1H), 2.38 (s, 3H), 1.34 (s, 18H); ^{13}C NMR (100 MHz, $CDCl_3$) δ 154.3, 144.5, 137.6, 136.1, 135.1, 130.3, 129.2, 129.1, 127.0, 125.6 (d, *J* = 3.7 Hz), 122.7, 76.3, 34.3, 30.1, 21.6; ^{19}F NMR (377 MHz, $CDCl_3$) δ -62.72 (s). HRMS: m/z calcd for $C_{29}H_{34}F_3O_3S+$ $(M+H)^+$ 519.2175, found *m/z* 519.2168.

2,6-Di-*tert*-butyl-4-(tosyl(3,4,5-trimethoxyphenyl)methyl)phenol (3j)

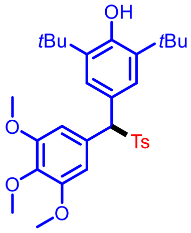

86% yield; ^{1}H NMR (400 MHz, $CDCl_3$) δ 7.43 (d, *J* = 8.2 Hz, 2H), 7.16 (t, *J* = 4.0 Hz, 4H), 6.88 (s, 2H), 5.24 (s, 1H), 5.08 (s, 1H), 3.85

(s, 6H), 3.83 (s, 3H), 2.37 (s, 3H), 1.35 (s, 18H); ^{13}C NMR (100 MHz, $CDCl_3$) δ 154.2, 153.12, 144.2, 138.1, 135.8, 135.5, 129.1, 128.7, 127.0, 123.3, 107.2, 60.9, 56.0, 34.3, 30.1, 21.5. HRMS: m/z calcd for $C_{31}H_{41}O_6S^+$ $(M+H)^+$ 541.2618, found *m/z* 541.2624.

2,6-Di-*tert*-butyl-4-((3,4-dimethylphenyl)(tosyl)methyl)phenol (**3k**)

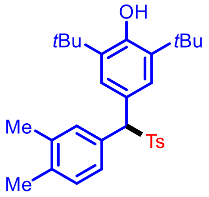

91% yield; ^{1}H NMR (400 MHz, $CDCl_3$) δ 7.45 – 7.37 (m, 3H), 7.35 (s, 1H), 7.12 (dd, J = 6.9, 3.6 Hz, 5H), 5.19 (s, 1H), 5.09 (s, 1H), 2.36 (s, 3H), 2.23 (s, 6H), 1.34 (s, 18H); ^{13}C NMR (100 MHz, $CDCl_3$) δ 154.0, 143.9, 136.9 (d, J = 9.7 Hz), 135.7, 131.3, 130.7, 129.9, 129.1 (d, J = 12.0 Hz), 127.2, 127.0, 123.8, 76.7, 34.3, 30.1, 21.5, 19.9, 19.5. HRMS: m/z calcd for $C_{30}H_{39}O_3S^+$ $(M+H)^+$ 479.2614, found *m/z* 479.2612.

2,6-Di-*tert*-butyl-4-(naphthalen-2-yl(tosyl)methyl)phenol (**3l**)

90% yield; ^{1}H NMR (400 MHz, $CDCl_3$) δ 8.06 (s, 1H), 7.85 – 7.77 (m, 4H), 7.49 –

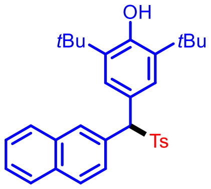

7.45 (m, 4H), 7.21 (s, 2H), 7.12 (d, J = 8.0 Hz, 2H), 5.35 (s, 1H), 5.23 (s, 1H), 2.35 (s, 3H), 1.35 (s, 18H); ^{13}C NMR (100 MHz, $CDCl_3$) δ 154.1, 144.1, 135.9, 135.6, 133.2, 133.0, 131.1, 129.6, 129.1 (d, J = 2.6 Hz), 128.3 (d, J = 5.6 Hz), 127.6, 127.2 (d, J = 2.0 Hz), 126.5 126.3, 123.5, 76.9, 34.3, 30.1, 21.5. HRMS: m/z calcd for $C_{32}H_{37}O_3S^+$ $(M+H)^+$ 501.2458, found *m/z* 501.2453.

2,6-Di-*tert*-butyl-4-(thiophen-2-yl(tosyl)methyl)phenol (**3m**)

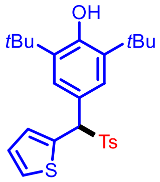

87% yield; ^{1}H NMR (400 MHz, DMSO-d_6) δ 7.56 (dd, J = 5.1, 1.1 Hz, 1H), 7.40 (d, J = 8.2 Hz, 2H), 7.25 (d, J = 8.1 Hz, 2H), 7.21 (s, 2H), 7.17 (d, J = 2.8 Hz, 1H), 7.09 (s, 1H), 7.01 (dd, J = 5.1, 3.6 Hz, 1H), 6.18 (s, 1H), 2.32 (s, 3H), 1.30 (s, 18H); ^{13}C NMR (100 MHz, DMSO-d_6) δ 154.6, 144.4, 139.2, 135.2 (d, J = 5.2 Hz), 130.3, 129.5, 129.1, 128.1, 127.1 (d, J = 8.3 Hz), 124.2, 70.5, 34.9, 30.6, 21.5. HRMS: m/z calcd for $C_{26}H_{33}O_3S_2^+$ $(M+H)^+$ 457.1866, found *m/z* 457.1878.

2,6-Di-*tert*-butyl-4-(2-isocyano-1-phenyl-2-tosylethyl)phenol (**±-4a**)

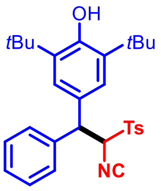

82% yield; dr = 1/1, ^{1}H NMR (400 MHz, $CDCl_3$) δ 7.54 (d, *J* = 8.3 Hz, 1.25H), 7.48 (d, *J* = 8.3 Hz, 1H), 7.35 (dd, *J* = 7.9, 1.4 Hz, 1.38H), 7.26 (dd, *J* = 12.6, 4.8 Hz, 2.4H), 7.19 (dd, *J* = 7.2, 2.1 Hz, 1.65H), 7.16–7.02 (m, 4.4H), 5.10 (tdd, *J* = 13.8, 7.8, 5.6 Hz, 2H), 4.76 (dd, *J* = 4.6, 1.9 Hz, 1H), 2.32 (d, *J* = 9.9 Hz, 3H), 1.30 (d, *J* = 7.2 Hz, 18H) ppm; ^{13}C NMR (100 MHz, $CDCl_3$) δ 167.9, 153.6, 153.3, 146.0, 145.9, 139.3, 137.4, 136.3, 135.7, 132.6, 132.0, 130.2, 129.8, 129.6, 129.5, 129.3, 129.1, 129.0, 128.5, 127.9, 127.8, 127.6, 127.1, 126.8, 126.3, 124.74, 77.9, 49.8, 49.5, 34.5, 34.4, 30.3, 30.2, 30.1, 21.8 ppm; HRMS (ESI) m/z calcd for $C_{30}H_{36}NO_3S^+$ $(M+H)^+$ 490.2410, found *m/z* 490.2416.

2,6-Di-*tert*-butyl-4-(2-isocyano-1-phenyl-2-tosylethyl)phenol (**4a-up**)

^{1}H NMR (400 MHz, $CDCl_3$) δ 7.56 (d, *J* = 8.3 Hz, 2H), 7.39–7.31 (m, 4H), 7.27 (d, *J* = 7.1 Hz, 1H), 7.20 (d, *J* = 8.2 Hz, 2H), 7.17 (s, 2H), 5.19 (d, *J* = 4.9 Hz, 1H), 5.17 (s, 1H), 4.84 (d, *J* = 4.9 Hz, 1H), 2.39 (s, 3H), 1.38 (s, 18H) ppm; ^{13}C NMR (100 MHz, $CDCl_3$) δ167.9, 153.6, 145.9, 139.3, 135.7, 132.0, 130.2, 129.5, 129.0, 127.9, 127.6, 126.8, 126.3, 77.9, 49.8, 34.4, 30.2, 21.8 ppm; HRMS (ESI) m/z calcd for $C_{30}H_{36}NO_3S^+$ $(M+H)^+$ 490.2410, found *m/z* 490.2410.

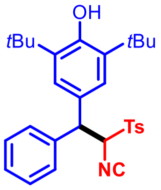

2,6-Di-*tert*-butyl-4-(2-isocyano-1-phenyl-2-tosylethyl)phenol (**4a-down**)

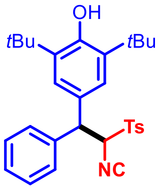

^{1}H NMR (400 MHz, $CDCl_3$) δ 7.63 (d, *J* = 8.3 Hz, 2H), 7.48–7.41 (m, 2H), 7.29 (ddd, *J* = 5.5, 4.2, 2.5 Hz, 4H), 7.24 (s, 1H), 7.16 (s, 2H), 5.19 (s, 1H), 5.14 (d, *J* = 4.8 Hz, 1H), 4.85 (d, *J* = 4.8 Hz, 1H), 2.42 (s, 3H), 1.40 (s, 18H) ppm; ^{13}C NMR (100 MHz, $CDCl_3$) δ167.9, 153.3, 146.0, 137.4, 136.3, 132.6, 129.8, 129.5, 129.3, 128.5, 127.8, 124.7, 77.22, 49.5, 34.4, 30.2, 21.8 ppm; HRMS (ESI) m/z calcd for $C_{30}H_{36}NO_3S^+$ $(M+H)^+$ 490.2410, found *m/z* 490.2420.

2,6-Di-*tert*-butyl-4-(2-isocyano-1-(*p*-tolyl)-2-tosylethyl)phenol (**4b**)

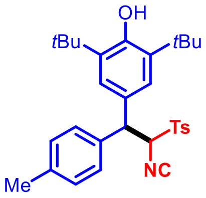

85% yield; dr = 2/1, ^{1}H NMR (400 MHz, $CDCl_3$) δ 7.58–7.52 (m, 0.63H), 7.46–7.39 (m, 1.09H), 7.37–7.32 (m, 1.11H), 7.27–7.22 (m, 0.70H), 7.16 (d, *J* = 8.4 Hz, 0.75H), 7.12–6.97 (m, 5.87H), 5.13–4.96 (m, 2.47H), 4.73 (d, *J* = 4.5 Hz, 0.49H), 2.34-2.23 (m, 6H), 1.31 (s, 6H), 1.27 (s, 12H) ppm; ^{13}C NMR (100 MHz, $CDCl_3$) δ 167.8, 154.0, 153.3, 146.0, 144.0, 138.3, 137.5, 136.3, 135.8, 135.7, 134.5, 132.7, 130.5, 129.8, 129.4, 129.1, 127.1, 124.7, 123.7, 76.6, 49.2, 34.5, 34.3, 30.2, 30.2, 21.8, 21.6, 21.1 ppm; HRMS: m/z calcd for $C_{31}H_{38}NO_3S^+$ $(M+H)^+$ 504.2567, found *m/z* 504.2568.

2,6-Di-*tert*-butyl-4-(1-(4-(*tert*-butyl)phenyl)-2-isocyano-2-tosylethyl)phenol (**4c**)

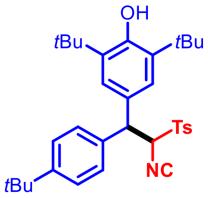

88% yield; dr = 1.7/1, ^{1}H NMR (400 MHz, $CDCl_3$) δ 7.58–7.41 (m, 2H), 7.36–7.23 (m, 3H), 7.18 (t, *J* = 3.9 Hz, 1H), 7.13–6.98 (m, 4H), 5.19–4.99 (m, 2.38H), 4.70 (d, *J* = 4.4 Hz, 0.51H), 2.39–2.12 (m, 3H), 1.32-1.27 (m, 18H), 1.22-1.21 (m, 9H) ppm; ^{13}C NMR (100 MHz, $CDCl_3$) δ 167.9, 154.0, 153.3, 151.4, 150.5, 145.7, 143.9, 136.2, 135.8, 135.6, 134.2, 132.5, 130.2, 129.9, 129.7, 129.6, 129.1, 129.0, 128.8, 127.0, 125.7, 125.4, 124.8, 123.7, 77.7, 49.4, 34.6, 34.4, 34.3, 31.4, 31.3, 30.3, 30.2, 30.1, 21.8, 21.5 ppm; HRMS: m/z calcd for $C_{34}H_{44}NO_3S+$ $(M+H)^+$ 546.3036, found *m/z* 546.3043.

2,6-Di-*tert*-butyl-4-(2-isocyano-1-(2-methoxyphenyl)-2-tosylethyl)phenol (**4d**)

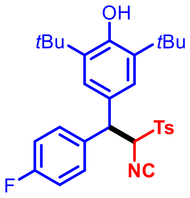

79% yield; ^{1}H NMR (400 MHz, $CDCl_3$) δ 7.63 (ddd, *J* = 11.7, 8.8, 5.2 Hz, 2.25H), 7.41 (dd, *J* = 9.9, 5.1 Hz, 2H), 7.29 (d, *J* = 8.1 Hz, 1H), 7.14 (dd, *J* = 8.7, 5.4 Hz, 3.42H), 7.08–6.95 (m, 2.26H), 5.25-5.22 (m, 1H), 5.19-5.17 (m, 1H), 5.10-4.90 (m, 1H), 2.44 (s, 1.16H), 2.37 (s, 1.84H), 1.40 (s, 6.81), 1.36 (s, 11.2H) ppm; ^{13}C NMR (100 MHz, $CDCl_3$) δ 168.2, 168.1, 161.6-160.8 (m), 154.2, 153.7, 153.4, 146.3, 146.0, 144.3, 136.5, 136.0, 135.4, 135.1, 133.1, 132.6, 132.0, 131.7, 131.1, 130.2, 129.9, 129.8, 129.6, 129.2, 129.1, 127.0, 126.8, 126.2, 124.6, 123.2, 115.6 (q, *J* = 21.2 Hz), 75.9,

49.2, 48.3, 34.5, 34.3, 30.2, 30.1, 21.8, 21.6 ppm; ^{19}F NMR (377 MHz, $CDCl_3$) δ -113.3 (s), -114.5 (s). HRMS: m/z calcd for $C_{30}H_{35}FNO_3S+$ $(M+H)^+$ 508.2316, found *m/z* 508.2319.

4-(1-(4-Bromophenyl)-2-isocyano-2-tosylethyl)-2,6-di-*tert*-butylphenol (4e)

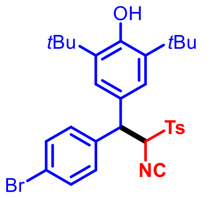

80% yield; ^{1}H NMR (400 MHz, $CDCl_3$) δ 7.56 (d, *J* = 7.7 Hz, 1H), 7.42 (q, *J* = 8.3 Hz, 2H), 7.38–7.30 (m, 2H), 7.24–7.20 (m, 1H), 7.13 (d, *J* = 7.9 Hz, 1H), 7.07 (d, *J* = 7.8 Hz, 1H), 7.02 (d, *J* = 8.8 Hz, 2H), 5.16 (d, *J* = 12.8 Hz, 1H), 5.12–5.05 (m, 1H), 5.03–4.64 (m, 1H), 2.35 (s, 1.35H), 2.28 (s, 1.56H), 1.31 (s, 7.8H), 1.26 (s, 9.42H) ppm; ^{13}C NMR (101 MHz, $CDCl_3$) δ 168.3, 168.1, 154.2, 153.7, 153.5, 146.3, 146.1, 136.5, 136.3, 136.0, 135.3, 132.6, 132.4, 132.0, 131.8, 131.6, 131.6, 131.1, 130.1, 129.9, 129.8, 129.6, 129.2, 129.1, 127.0, 126.1, 124.6, 122.9, 122.8, 122.0, 76.0, 49.5, 48.5, 34.5, 34.4, 34.3, 30.2, 30.2, 30.1, 21.8, 21.6 ppm; HRMS: m/z calcd for $C_{30}H_{35}BrNO_3S^+$ $(M+H)^+$ 568.1516 (100%), $C_{30}H_{35}{}^{81}BrNO_3S^+$ $(M+H)^+$ 570.1495 (97.3%), found *m/z* 570.1495.

2,6-Di-*tert*-butyl-4-(2-isocyano-2-tosyl-1-(4-(trifluoromethyl)phenyl)ethyl)phenol (4f)

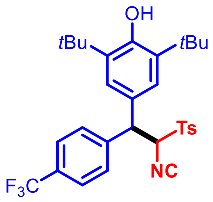

83% yield; ^{1}H NMR (400 MHz, $CDCl_3$) δ 7.71 (d, *J* = 8.2 Hz, 1H), 7.55 (dd, *J* = 8.1, 5.0 Hz, 2H), 7.51–7.42 (m, 3H), 7.37 (dd, *J* = 14.9, 8.3 Hz, 1H), 7.08-7.02 (t, *J* = 13.4 Hz, 3H), 5.19-5.13 (m, 1.67H), 5.06–4.77 (m, 1.46H), 2.34 (s, 1.81H), 2.29 (s, 1.18H), 1.32 (s, 11H), 1.27 (s, 7H) ppm; ^{13}C NMR (101 MHz, $CDCl_3$) δ 168.7, 154.3, 153.6, 146.4, 144.5, 141.2, 137.6, 136.6, 136.1, 135.1, 132.2, 130.3, 130.1, 129.9, 129.8, 129.8, 129.7, 129.2, 129.1, 128.7, 128.3, 127.0, 126.2, 122.5 (q, *J* = 298.6 Hz), 125.6, 125.6, 125.6, 125.5, 125.5, 125.4, 125.4, 125.3, 124.6, 122.7, 76.2, 49.9, 48.8, 34.5, 34.3, 30.2, 30.1, 21.7, 21.6 ppm; ^{19}F NMR (377 MHz, $CDCl_3$) δ -62.6 (s), -62.7 (s). HRMS (ESI) m/z calcd for $C_{31}H_{35}F_3NO_3S^+$ $(M+H)^+$ 558.2284, found *m/z* 558.2299.

4-(1-(4-Bromothiophen-2-yl)-2-isocyano-2-tosylethyl)-2,6-di-*tert*-butylphenol (4g)

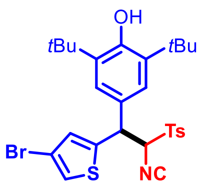

60% yield; ^{1}H NMR (400 MHz, $CDCl_3$) δ 7.60 (d, *J* = 8.3 Hz, 0.83H), 7.32 (d, *J* = 8.3 Hz, 1.07H), 7.22 (d, *J* = 8.1 Hz, 0.96H), 7.17 (d, *J* = 1.5 Hz, 0.52H), 7.13–7.05 (m, 2.70H), 7.01 (s, 1.37H), 6.94 (d, *J* = 0.9 Hz, 0.43H), 5.38–4.74 (m, 3H), 2.38 (s, 1.26H), 2.30 (s, 1.73H), 1.34 (s, 7.5H), 1.28 (s, 10.5H) ppm; ^{13}C NMR (101 MHz, $CDCl_3$) δ 169.2, 154.6, 153.9, 146.5, 144.5, 140.2, 136.6, 136.1, 135.9, 134.3, 132.0, 131.9, 130.2, 129.9, 129.8, 129.2, 129.2, 128.4, 127.0, 124.6, 124.3, 122.8, 122.4, 109.7, 109.5, 72.3, 45.2, 34.5, 34.3, 31.4, 30.2, 30.1, 30.1, 21.8, 21.6 ppm; HRMS (ESI) m/z calcd for $C_{28}H_{33}BrNO_3S_2^+$ $(M+H)^+$ 574.1080, found *m/z* 574.1082.

Methyl 3-(3,5-di-*tert*-butyl-4-hydroxyphenyl)-2-isocyano-3-phenylpropanoate (4h)

86% yield; ^{1}H NMR (400 MHz, $CDCl_3$) δ 7.43–7.26 (m, 5H), 7.12 (d, *J* = 7.9 Hz, 2H), 5.21 (s, 1H), 4.91 (dd, *J* = 6.9, 4.3 Hz, 1H), 4.60 (t, *J* = 7.4 Hz, 1H), 3.66 (d, *J* = 4.8 Hz, 3H), 1.44 (d, *J* = 4.2 Hz, 18H); ^{13}C NMR (101 MHz, $CDCl_3$) δ 166.5, 166.4, 161.9, 153.3, 153.2, 139.2, 138.4, 136.1, 135.9, 129.1, 128.7, 128.7, 128.5, 128.1, 128.1, 127.6, 127.5, 125.3, 124.8, 61.3, 53.2, 53.2, 53.0, 34.4, 30.3, 30.3 ppm; HRMS (ESI) m/z calcd for $C_{25}H_{32}NO_3^+$ $(M+H)^+$ 394.2377, found *m/z* 394.2377.

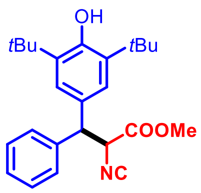

3-(3,5-Di-*tert*-butyl-4-hydroxyphenyl)-2,2-difluoro-1-(naphthalen-2-yl)-3-(*p*-tolyl) propan-1-one (5)

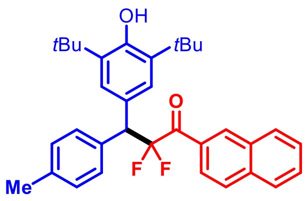

83% yield; ^{1}H NMR (400 MHz, $CDCl_3$) δ 8.35 (s, 1H), 7.88 – 7.81 (m, 4H), 7.60 (t, *J* = 7.1 Hz, 1H), 7.53 (t, *J* = 7.2 Hz, 1H), 7.35 (d, *J* = 7.9 Hz, 2H), 7.12 (d, *J* = 5.8 Hz, 4H), 5.08 (s, 1H), 4.88 (dd, *J* = 19.6, 16.9 Hz, 1H), 2.31 (s, 3H), 1.31 (s, 18H) ppm; ^{13}C NMR (100 MHz, $CDCl_3$) δ 191.1 (t, *J* = 29.4 Hz), 153.2, 137.0, 135.7, 135.6, 133.8 (d, *J* = 3.6 Hz), 132.2, 132.0 (t, *J* = 5.1 Hz), 130.8, 129.9, 129.5, 129.2, 129.0, 128.2, 127.7, 126.8, 126.6, 126.4 (d, *J* = 4.7 Hz), 124.8, 124.2 (d, *J* =

48.5 Hz), 119.5 (dd, J = 283.9, 234.4 Hz), 55.20 (t, J = 21.6 Hz), 34.3, 30.2, 21.0 ppm; ^{19}F NMR (376 MHz, $CDCl_3$) δ -98.78 (dd, J = 267.7, 16.6 Hz, 1F), -100.58 (ddd, J = 287.5, 267.7, 18.4 Hz, 1F) ppm; HRMS (ESI) m/z: $[M+Na]^+$ calcd for $C_{34}H_{36}F_2NaO_2^+$ 537.2576; found 537.2579.

2-(3,5-Di-*tert*-butyl-4-hydroxyphenyl)-1-(1*H*-indol-2-yl)-2-(*p*-tolyl)ethan-1-one (**6**)

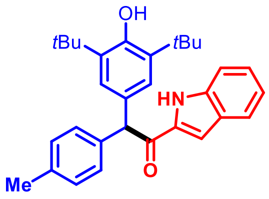

84% yield; ^{1}H NMR (400 MHz, $CDCl_3$) δ 9.96 – 9.43 (m, 1H), 7.56 (d, J = 8.1 Hz, 1H), 7.20 (t, J = 7.5 Hz, 3H), 7.17–7.13 (m, 1H), 7.10 (d, J = 4.6 Hz, 3H), 7.06–6.97 (m, 3H), 5.79 (s, 1H), 5.07 (s, 1H), 2.22 (s, 3H), 1.30 (s, 18H) ppm; ^{13}C NMR (101 MHz, $CDCl_3$) δ 192.2, 153.0, 137.7, 136.8, 136.6, 135.9, 135.2, 129.6, 129.4, 128.8, 127.6, 126.4, 125.7, 123.0, 120.9, 112.4, 110.2, 58.9, 34.4, 30.3, 21.1 ppm; HRMS (ESI) m/z calcd for $C_{31}H_{36}NO_2^+$ $(M+H)^+$ 454.2741, found *m/z* 454.2745.

2-(3,5-Di-*tert*-butyl-4-hydroxyphenyl)-1-phenyl-2-(*p*-tolyl)ethan-1-one (**7**)

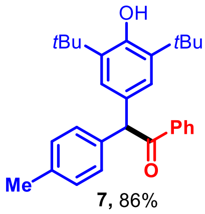

7, 86%

86% yield; ^{1}H NMR (400 MHz, $CDCl_3$) δ 8.00 (d, J = 7.7 Hz, 2H), 7.49 (t, J = 7.3 Hz, 1H), 7.39 (t, J = 7.6 Hz, 2H), 7.19 (d, J = 7.9 Hz, 2H), 7.12 (d, J = 7.9 Hz, 2H), 7.07 (s, 2H), 5.90 (s, 1H), 5.10 (s, 1H), 2.30 (s, 3H), 1.38 (s, 18H) ppm; ^{13}C NMR (101 MHz, $CDCl_3$) δ 199.0, 152.8, 137.3, 136.7, 136.5, 135.8, 132.7, 129.6, 129.4, 128.9, 128.9, 128.5, 125.8, 59.0, 34.4, 30.3, 21.1 ppm; HRMS (ESI) m/z calcd for $C_{29}H_{35}O_2^+$ $(M+H)^+$ 415.2632, found *m/z* 415.2629.

Crystal structure of diarylmethyl sulfone 3e.

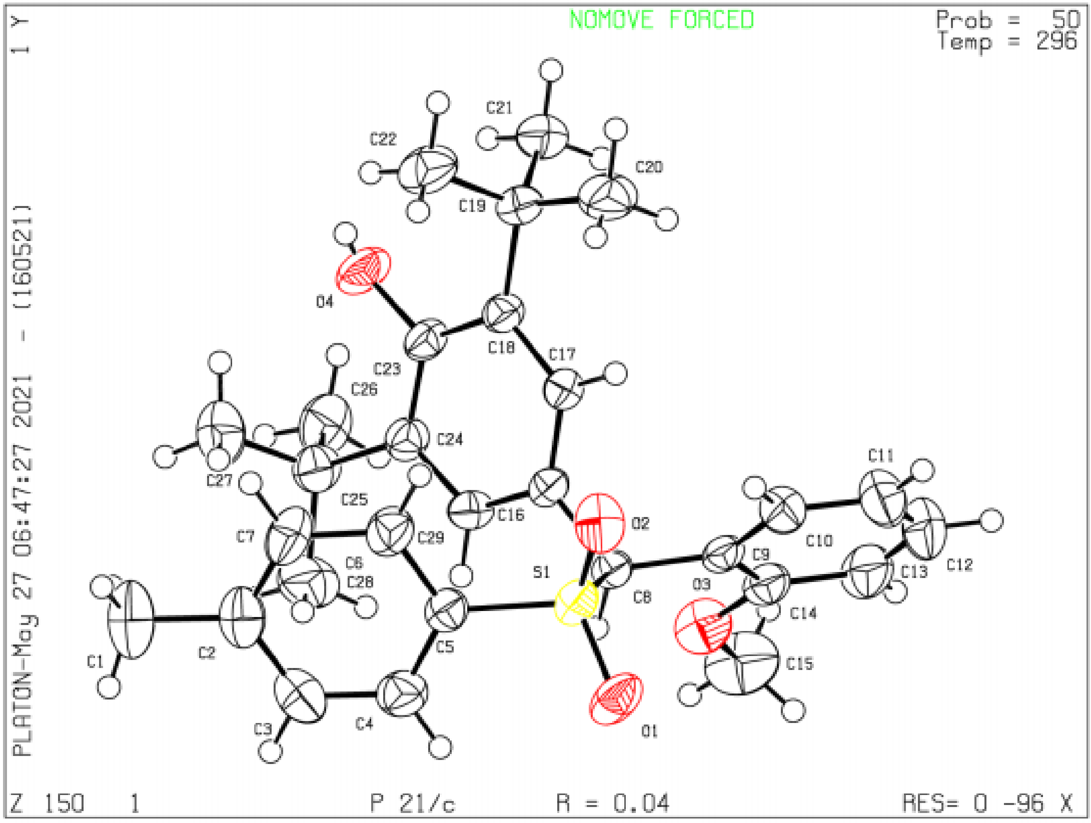

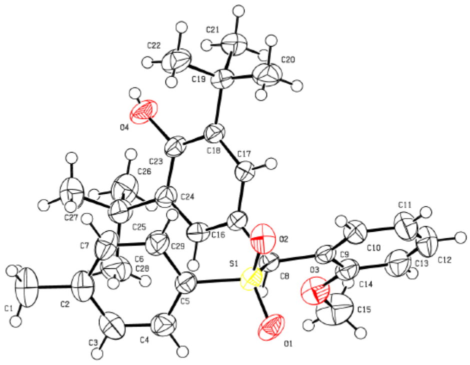

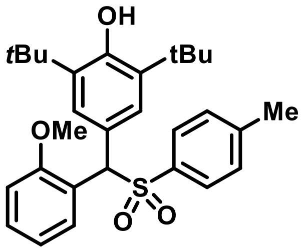

3e

Datablock: 1

Bond precision: C-C = 0.0027 A   Wavelength=0.71073

Cell:	a=11.7410(9)	b=11.8818(10)	c=19.9583(15)
	alpha=90	beta=93.939(1)	gamma=90
Temperature:	296 K		

	Calculated	Reported
Volume	2777.7(4)	2777.7(4)
Space group	P 21/c	P 21/c
Hall group	-P 2ybc	-P 2ybc
Moiety formula	C29 H36 O4 S	?
Sum formula	C29 H36 O4 S	C29 H36 O4 S
Mr	480.64	480.64
Dx,g cm-3	1.149	1.149
Z	4	4
Mu (mm-1)	0.147	0.147
F000	1032.0	1032.0
F000'	1032.94	
h,k,lmax	13,14,23	13,14,23
Nref	4881	4874
Tmin,Tmax	0.963,0.968	
Tmin'	0.963	

Correction method= Not given

Data completeness= 0.999   Theta(max)= 24.998

R(reflections)= 0.0387(3661)   wR2(reflections)= 0.1042(4874)

S = 1.099   Npar= 320

The following ALERTS were generated. Each ALERT has the format
test-name_ALERT_alert-type_alert-level.
Click on the hyperlinks for more details of the test.

Alert level C

```
PLAT222_ALERT_3_C NonSolvent Resd 1  H   Uiso(max)/Uiso(min) Range        6.1 Ratio
PLAT354_ALERT_3_C Short  O-H (X0.82,N0.98A)  O4        - H4      .       0.68 Ang.
PLAT414_ALERT_2_C Short Intra D-H..H-X        H4        ..H22A             1.93 Ang.
                                                        x,y,z  =        1_555 Check
PLAT601_ALERT_2_C Unit Cell Contains Solvent Accessible VOIDS of .          50 Ang**3
PLAT906_ALERT_3_C Large K Value in the Analysis of Variance ......      3.135 Check
PLAT911_ALERT_3_C Missing FCF Refl Between Thmin & STh/L=     0.595          7 Report
```

Alert level G

```
PLAT002_ALERT_2_G Number of Distance or Angle Restraints on AtSite         2 Note
PLAT172_ALERT_4_G The CIF-Embedded .res File Contains DFIX Records         1 Report
PLAT793_ALERT_4_G Model has Chirality at C8           (Centro SPGR)        S Verify
PLAT860_ALERT_3_G Number of Least-Squares Restraints .............         1 Note
PLAT883_ALERT_1_G No Info/Value for _atom_sites_solution_primary .     Please Do !
PLAT909_ALERT_3_G Percentage of I>2sig(I) Data at Theta(Max) Still       50% Note
PLAT910_ALERT_3_G Missing # of FCF Reflection(s) Below Theta(Min).         1 Note
PLAT913_ALERT_3_G Missing # of Very Strong Reflections in FCF ....         1 Note
PLAT933_ALERT_2_G Number of OMIT Records in Embedded .res File ...         5 Note
PLAT941_ALERT_3_G Average HKL Measurement Multiplicity ...........       2.8 Low
PLAT961_ALERT_5_G Dataset Contains no Negative Intensities .......     Please Check
PLAT965_ALERT_2_G The SHELXL WEIGHT Optimisation has not Converged     Please Check
PLAT978_ALERT_2_G Number C-C Bonds with Positive Residual Density.         8 Info
```

```
  0 ALERT level A = Most likely a serious problem - resolve or explain
  0 ALERT level B = A potentially serious problem, consider carefully
  6 ALERT level C = Check. Ensure it is not caused by an omission or oversight
 13 ALERT level G = General information/check it is not something unexpected

  1 ALERT type 1 CIF construction/syntax error, inconsistent or missing data
  6 ALERT type 2 Indicator that the structure model may be wrong or deficient
  9 ALERT type 3 Indicator that the structure quality may be low
  2 ALERT type 4 Improvement, methodology, query or suggestion
  1 ALERT type 5 Informative message, check
```

References

1. Kale, S. B.; Jori, P. K.; Thatikonda, T.; Gonnade, R. G.; Das, U. *Org. Lett.* **2019**, *21*, 7736.

2. Qu, C.; Song, G.; Tang, D.; Shao, J.; Li, H.; Xu, Z.; Chen, Z. *J. Org. Chem.*, **2020**, *85*, 12785-12796.

3. Guan, X.; Zhang, L.; You, P.; Liu, S.; Liu, Z. *Tetrahedron Lett.*, **2019**, *60*, 244-247.

Copies ^{1}H NMR, ^{13}C NMR, ^{19}F NMR

*2,6-Di-tert-butyl-4-(phenyl(tosyl)methyl)phenol (**3a**)*

^{1}H NMR (400 MHz, CDCl$_3$):

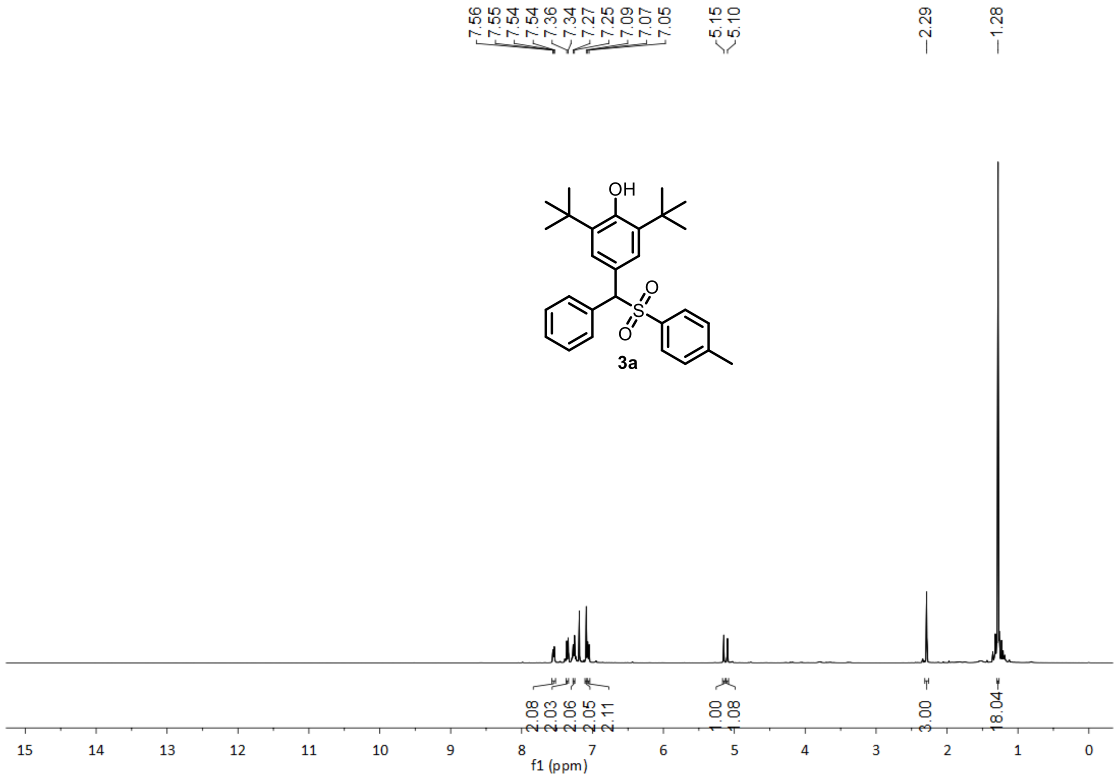

^{13}C NMR (100 MHz, CDCl$_3$):

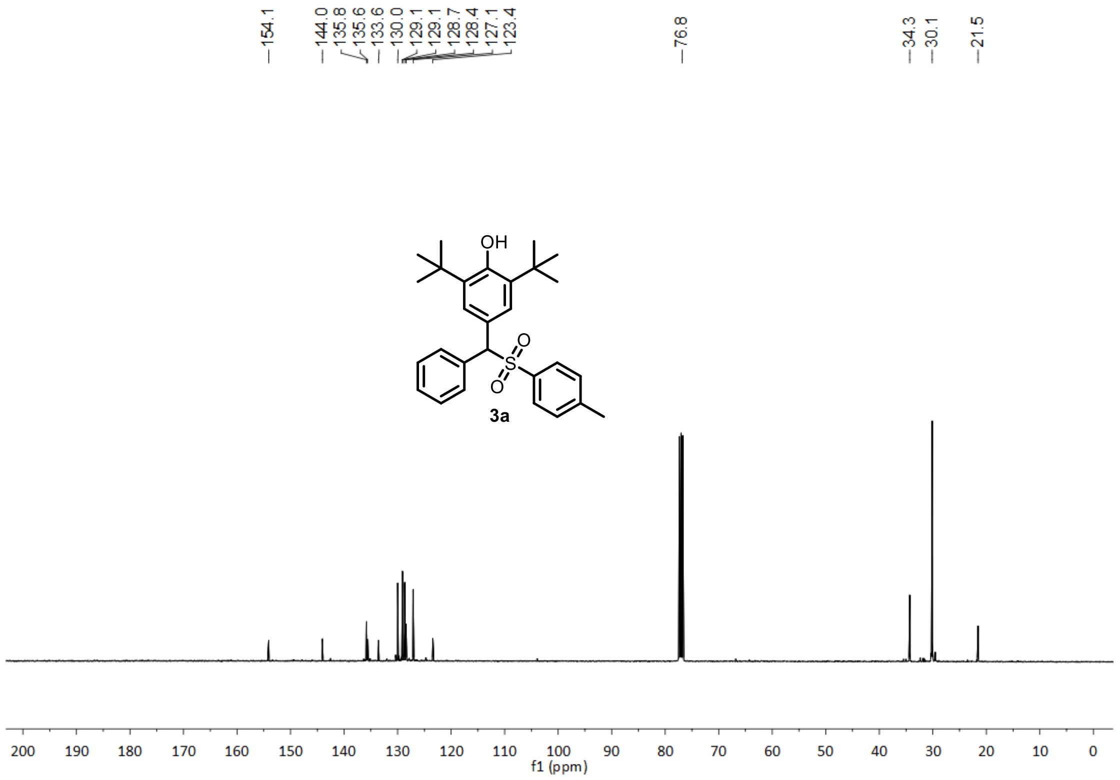

DEPT135

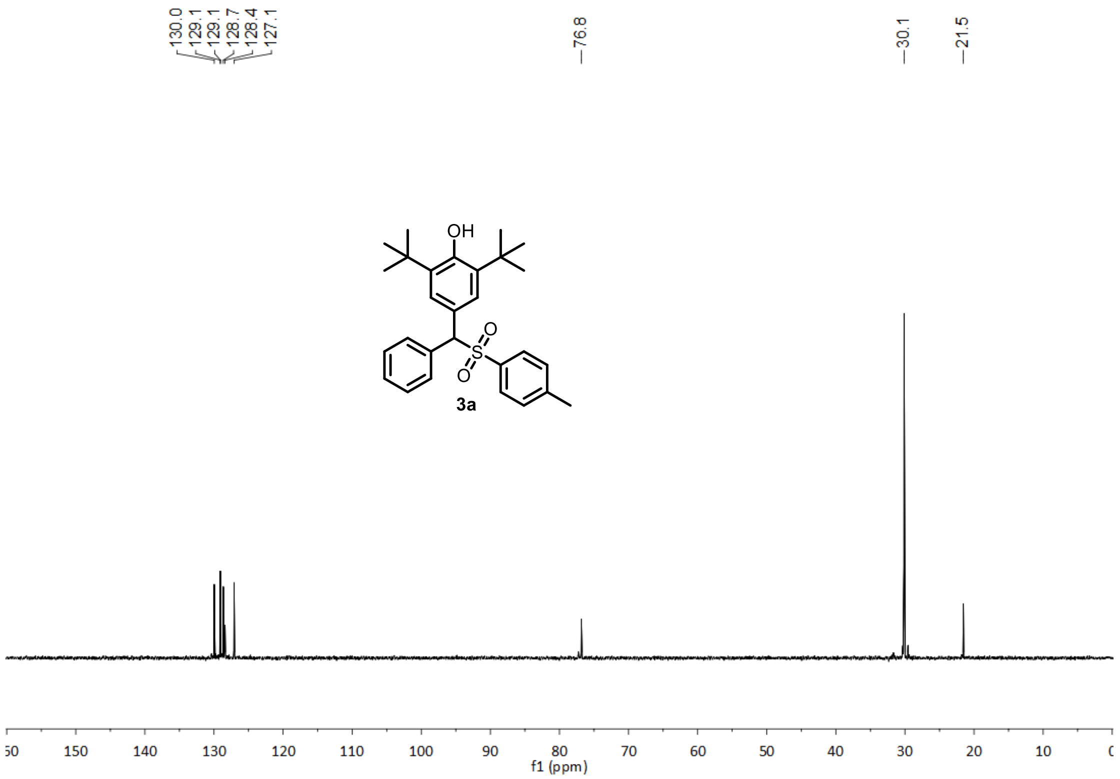

*2,6-Di-tert-butyl-4-(phenyl(tosyl)methyl)phenol (**3b**)*

^{1}H NMR (400 MHz, $CDCl_3$)

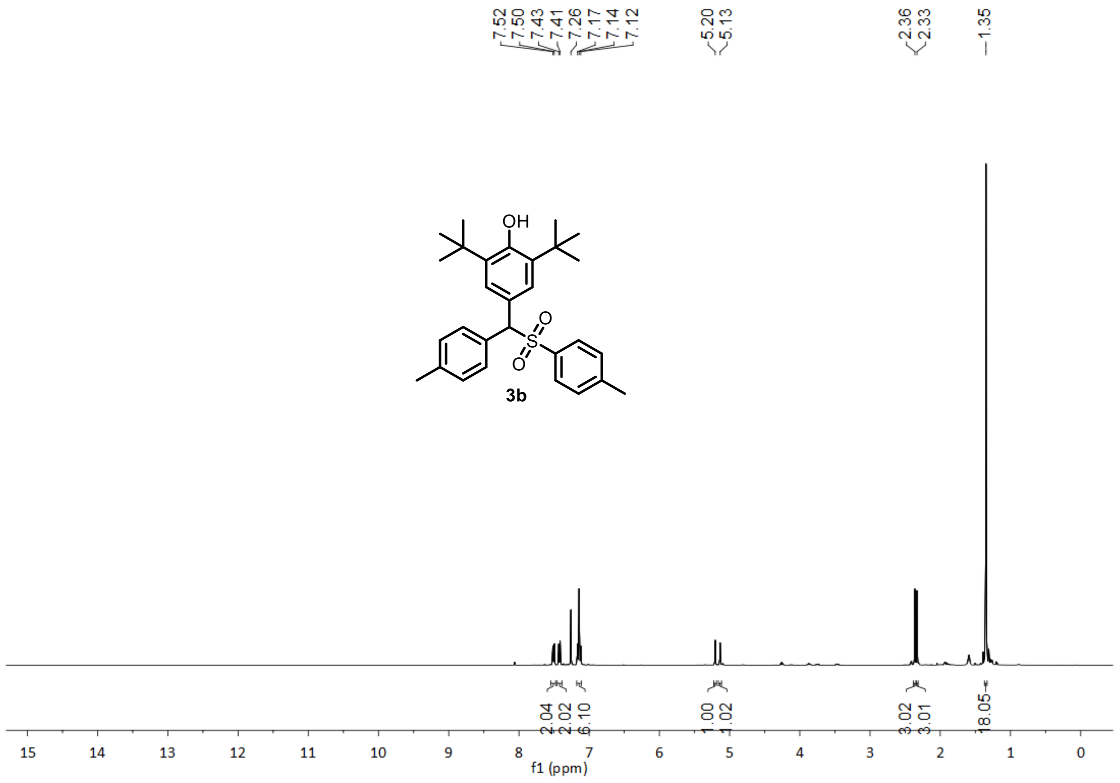

^{13}C NMR (100 MHz, $CDCl_3$):

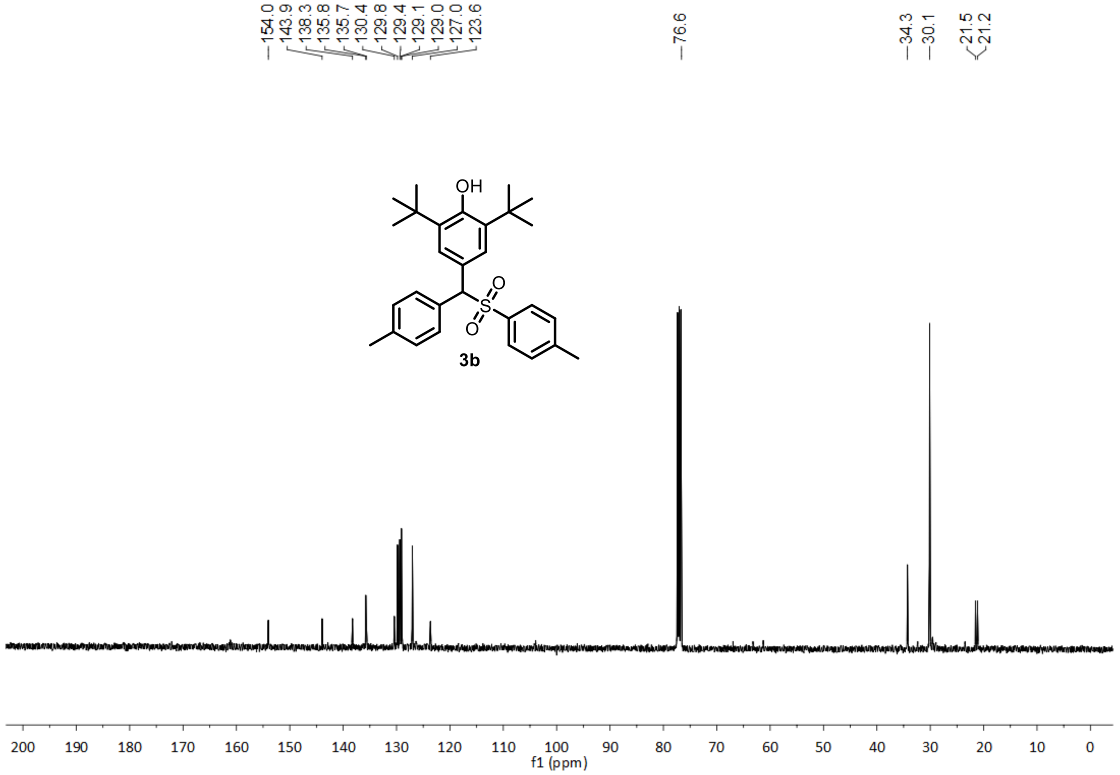

DEPT135

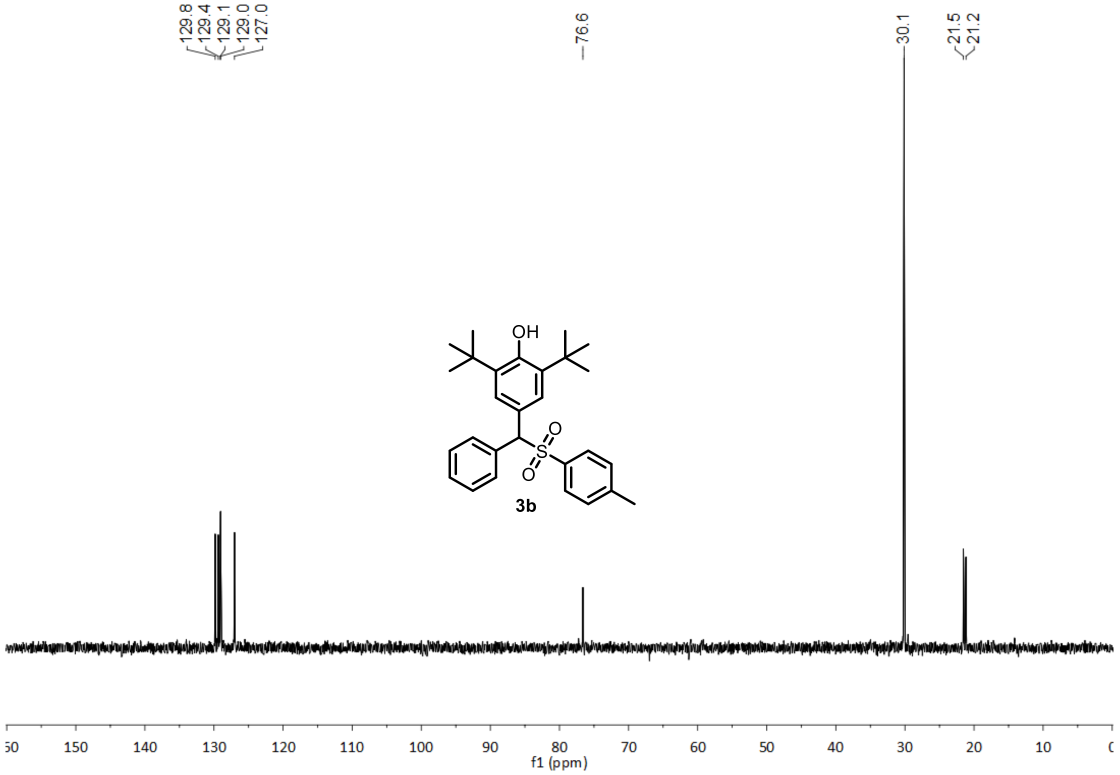

2,6-Di-*tert*-butyl-4-((4-methoxyphenyl)(tosyl)methyl)phenol (**3c**)

^{1}H NMR (400 MHz, $CDCl_3$)

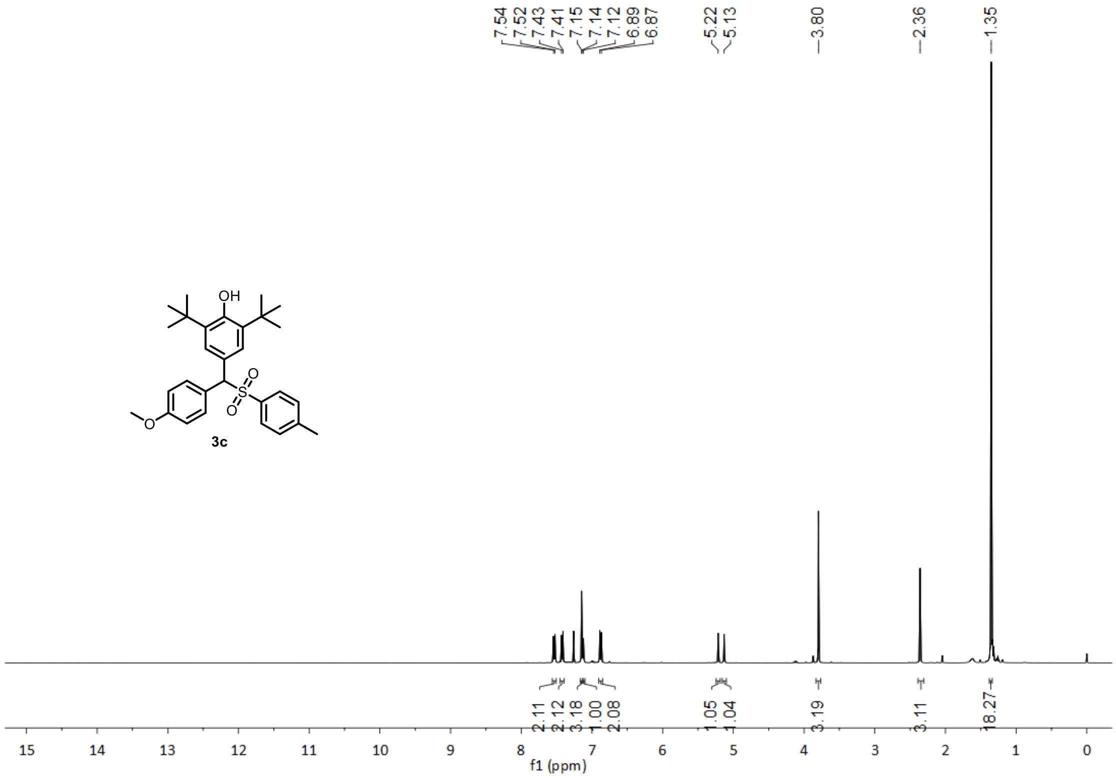

^{13}C NMR (100 MHz, $CDCl_3$):

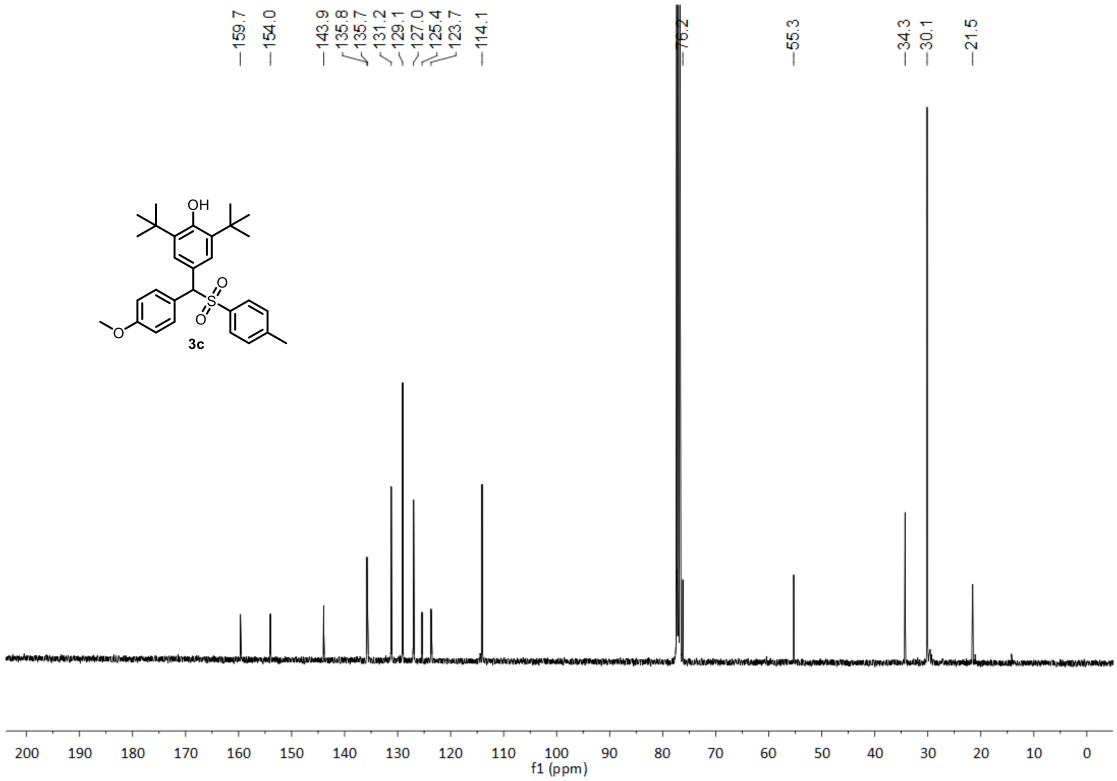

DEPT135

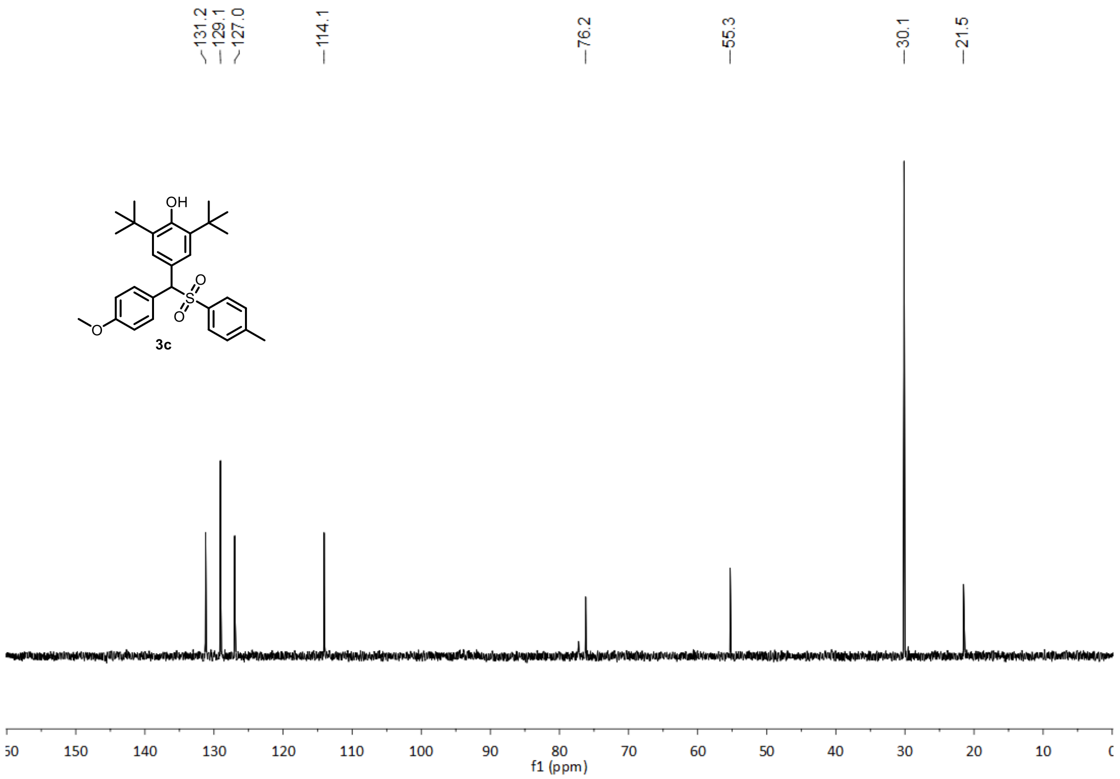

2,6-Di-*tert*-butyl-4-((4-(*tert*-butyl)phenyl)(tosyl)methyl)phenol (**3d**)

^{1}H NMR (400 MHz, $CDCl_3$)

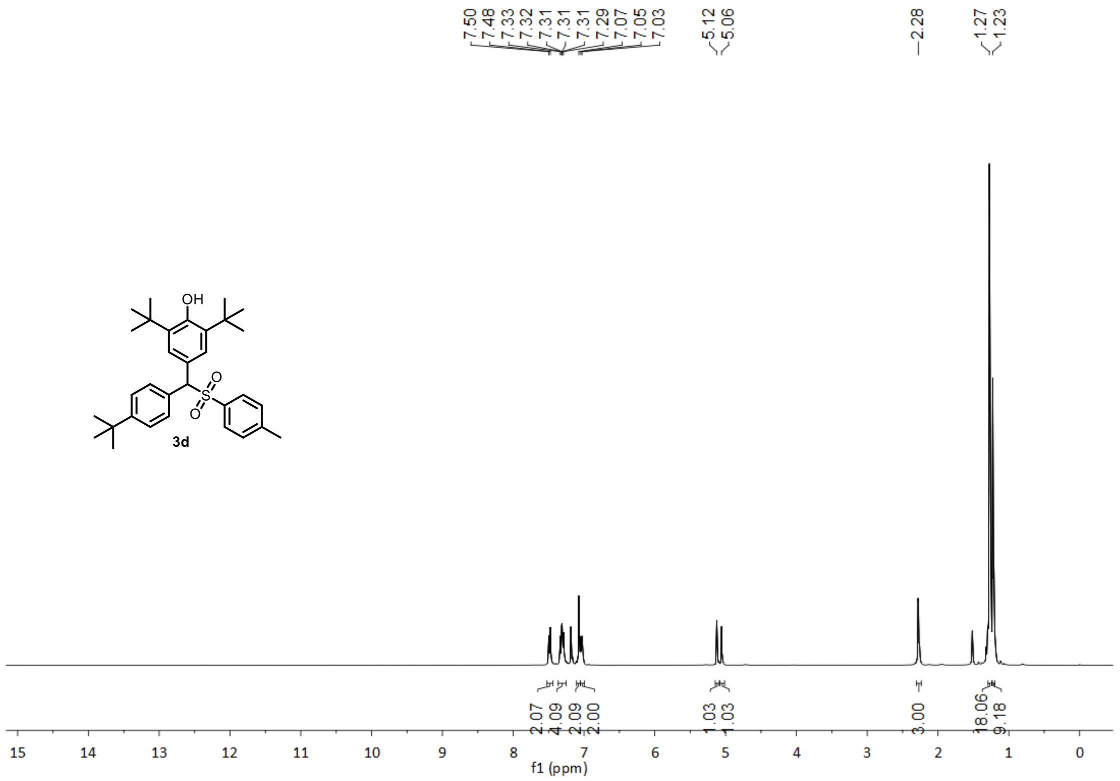

^{13}C NMR (100 MHz, $CDCl_3$):

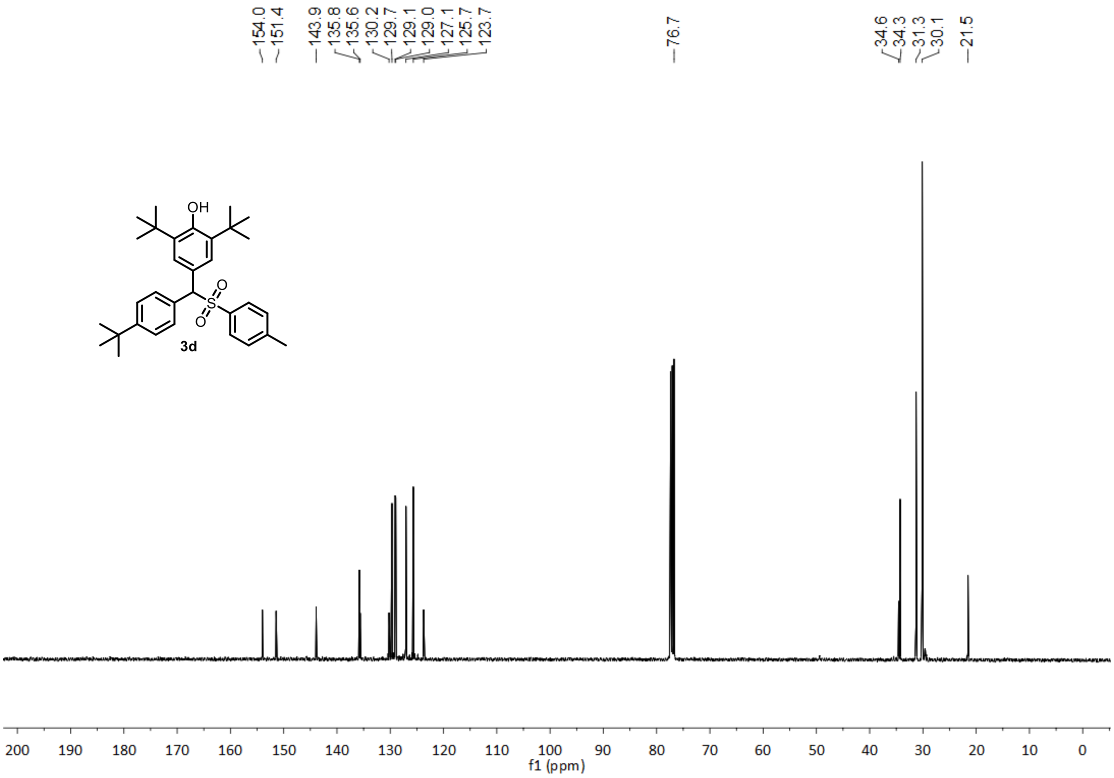

DEPT135

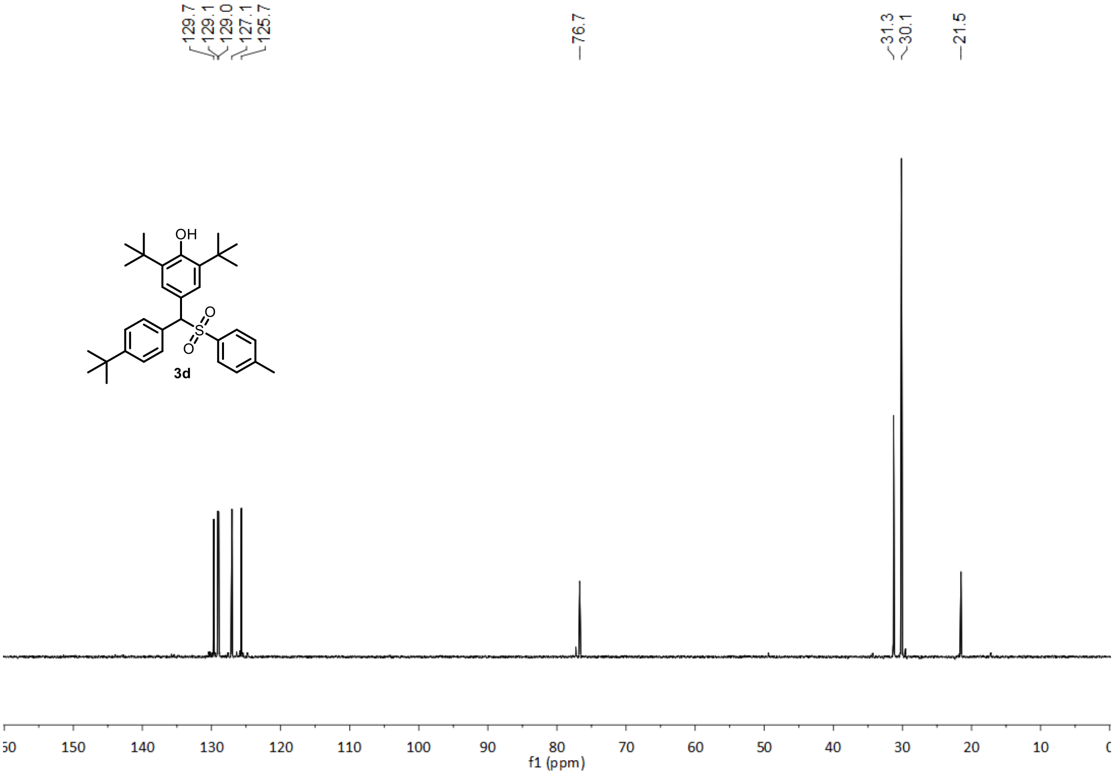

2,6-Di-*tert*-butyl-4-((2-methoxyphenyl)(tosyl)methyl)phenol (**3e**)

^{1}H NMR (400 MHz, CDCl$_3$)

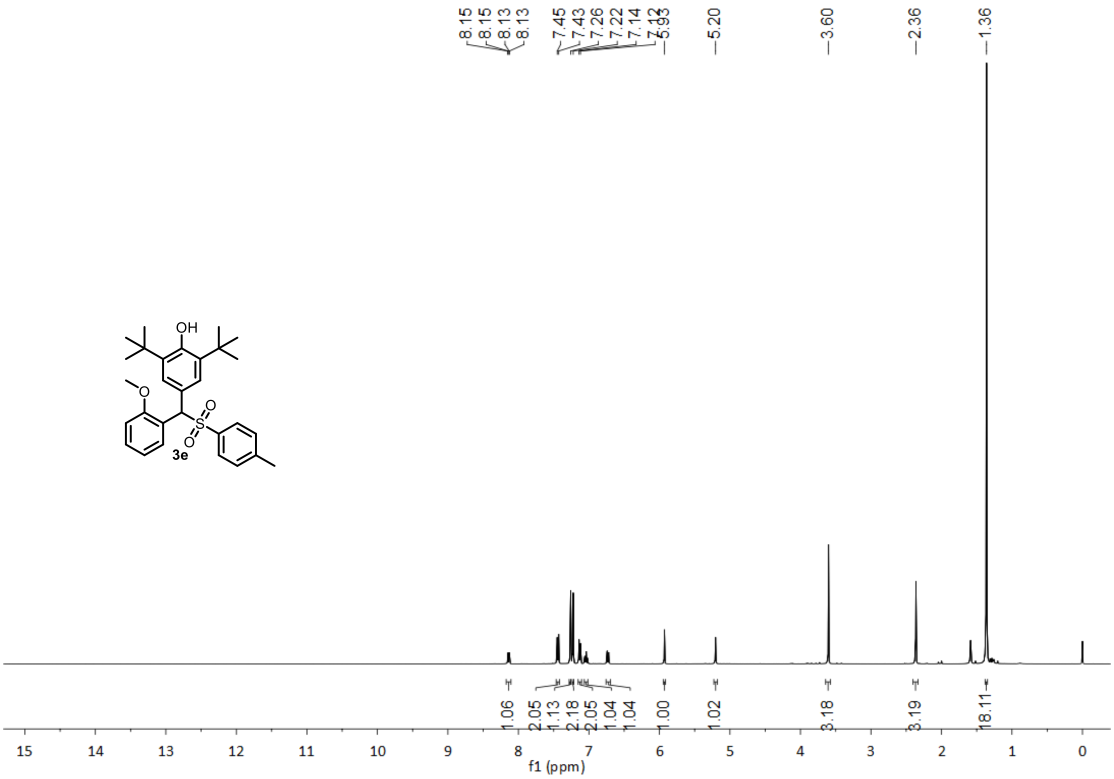

^{13}C NMR (100 MHz, CDCl$_3$):

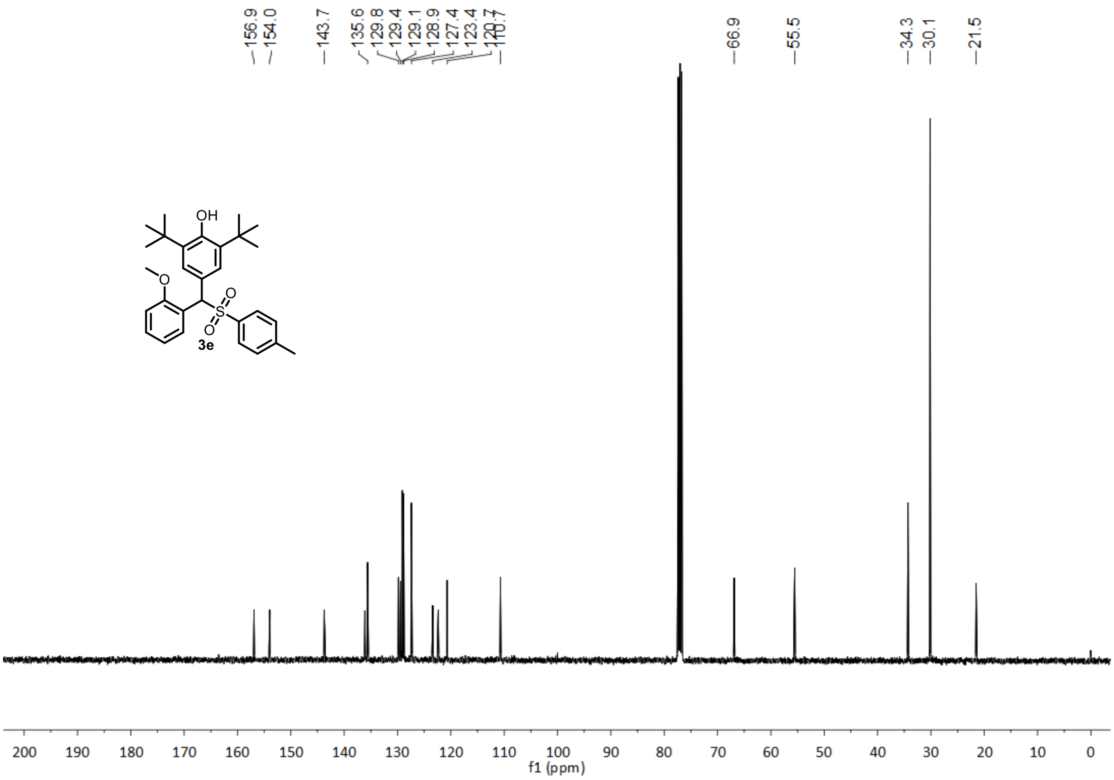

DEPT135

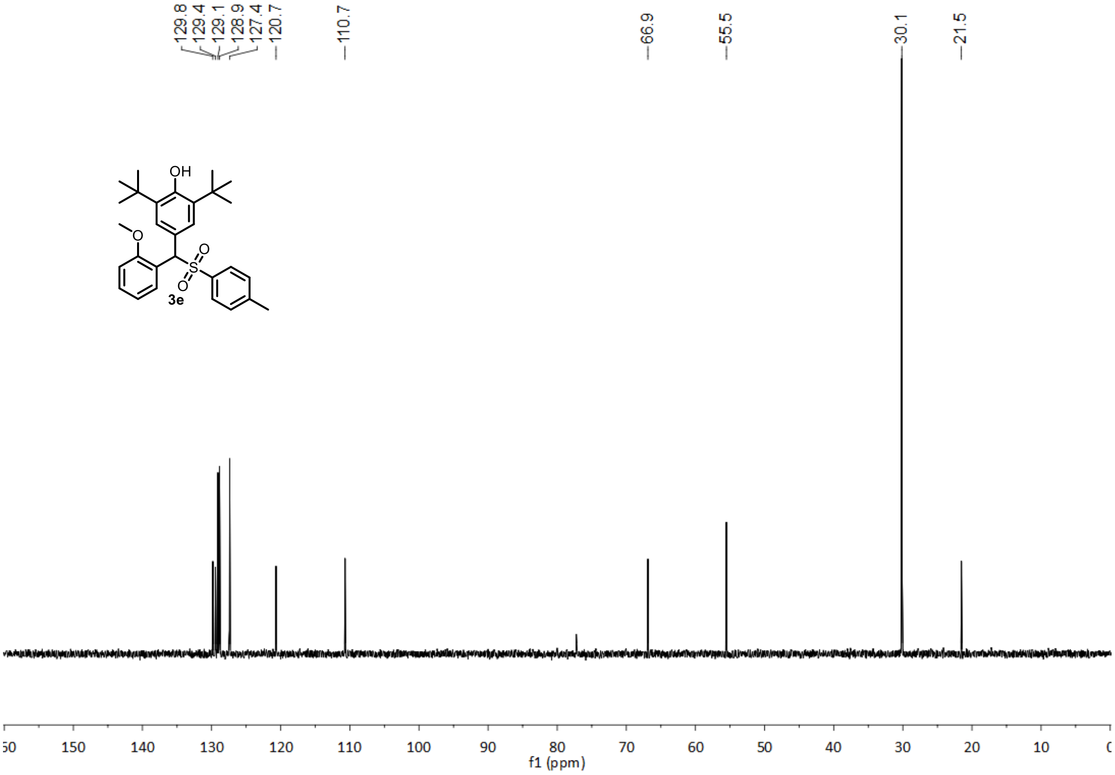

2,6-Di-*tert*-butyl-4-((4-chlorophenyl)(tosyl)methyl)phenol (**3f**)

^{1}H NMR (400 MHz, $CDCl_3$):

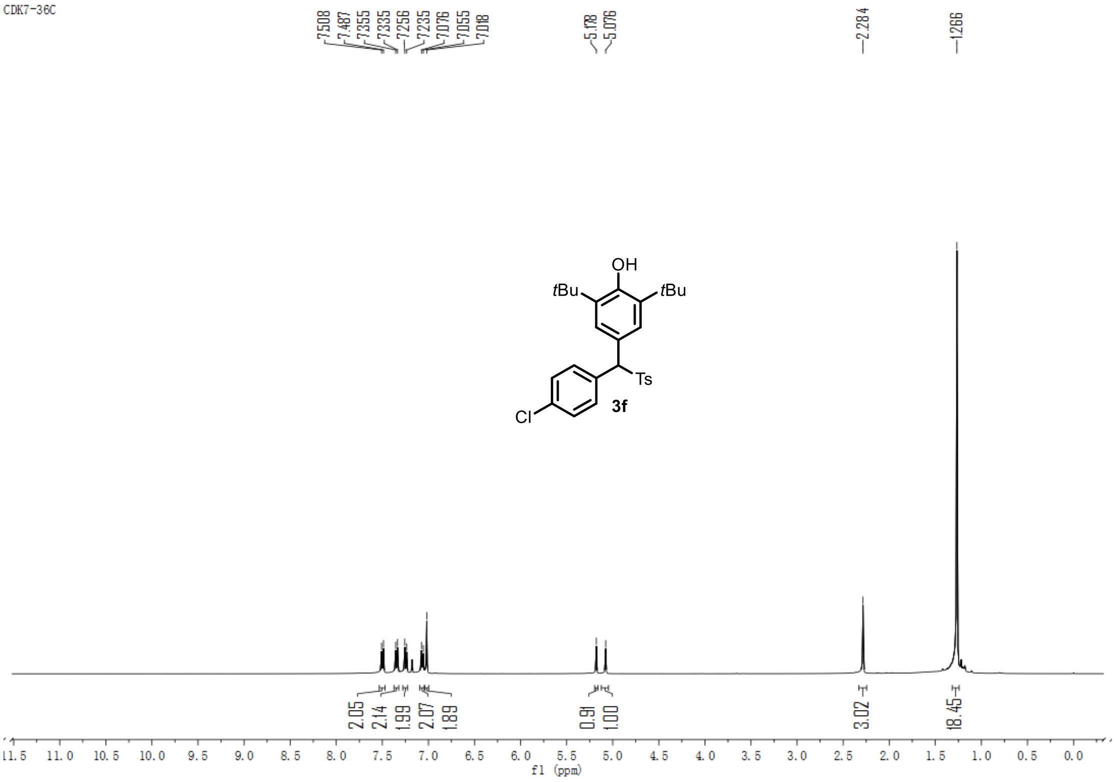

^{13}C NMR (100 MHz, $CDCl_3$):

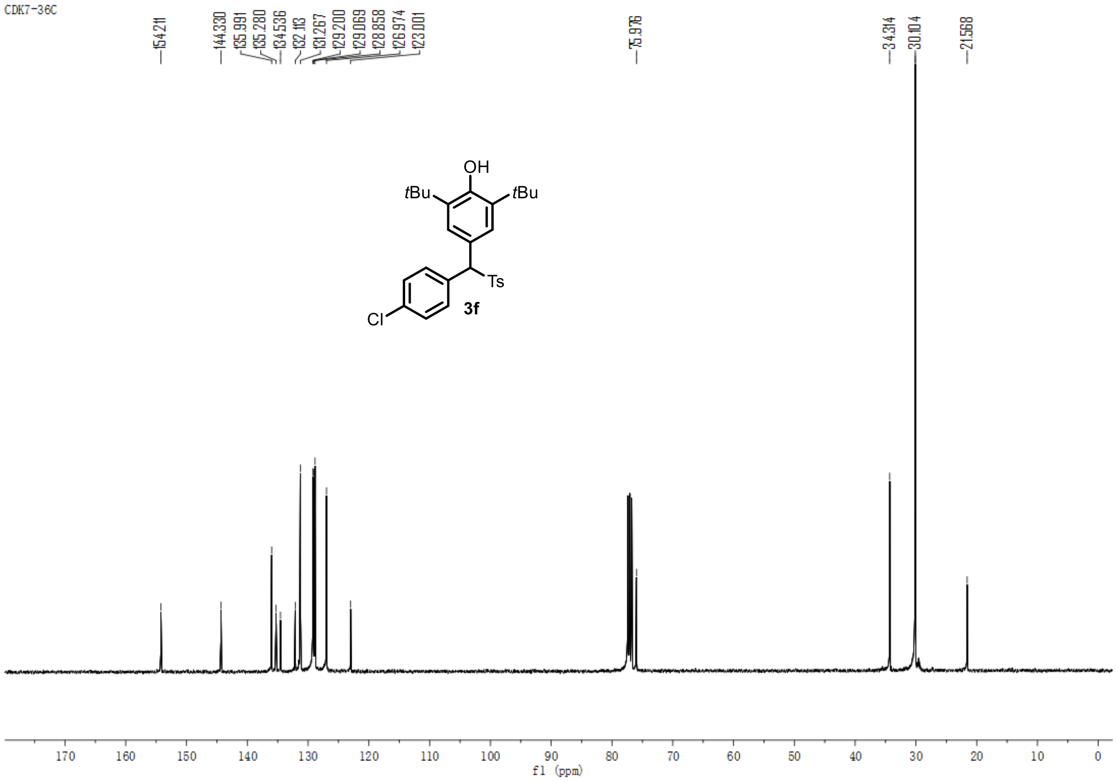

4-((2-Bromophenyl)(tosyl)methyl)-2,6-di-*tert*-butylphenol (**3g**)

^{1}H NMR (400 MHz, $CDCl_3$)

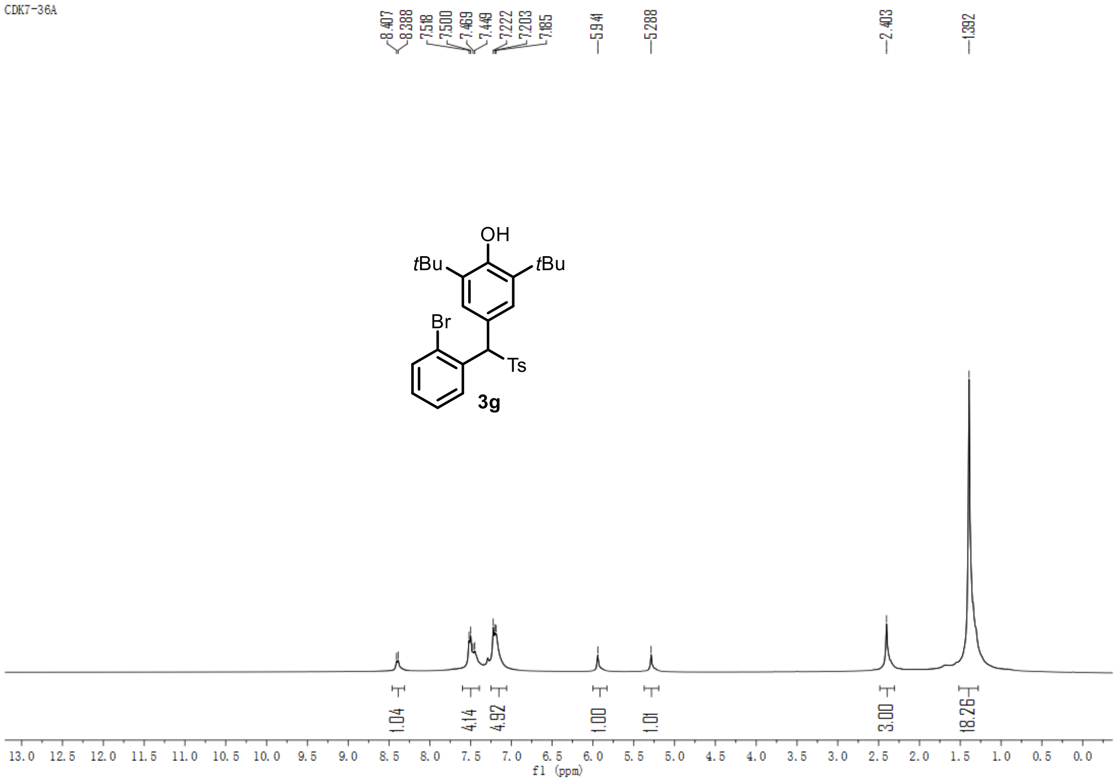

^{13}C NMR (100 MHz, $CDCl_3$):

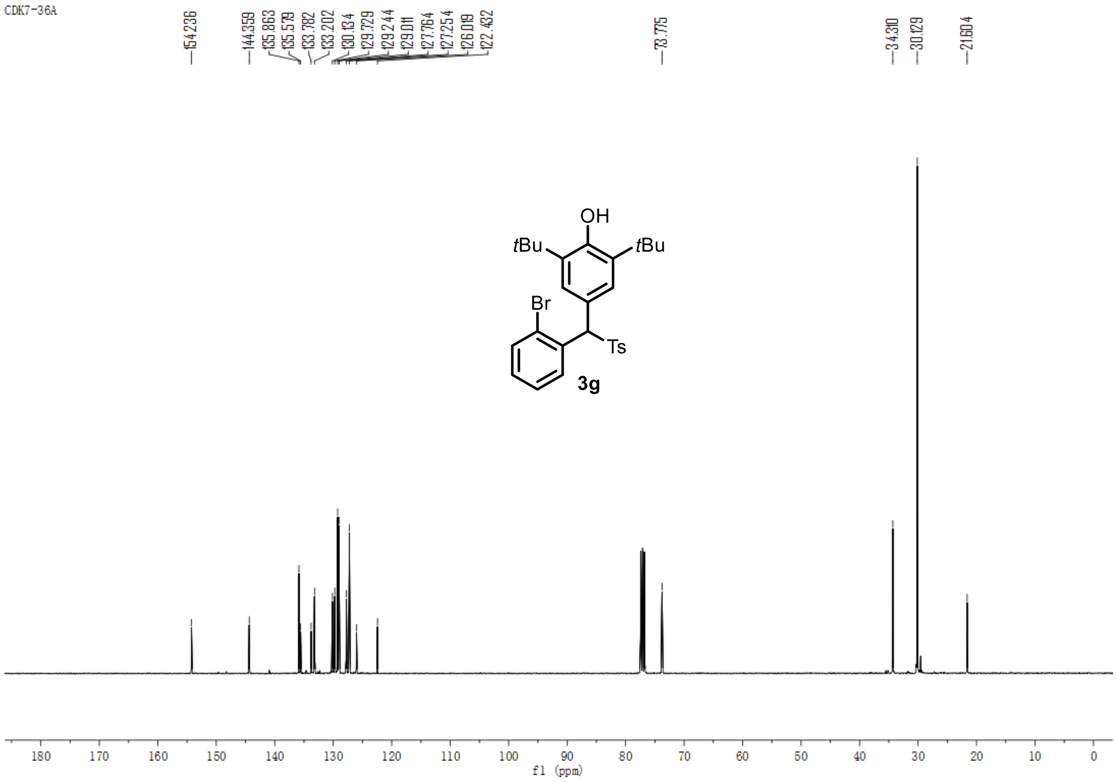

2,6-Di-*tert*-butyl-4-((3,4-dichlorophenyl)(tosyl)methyl)phenol (**3h**)

^{1}H NMR (400 MHz, $CDCl_3$)

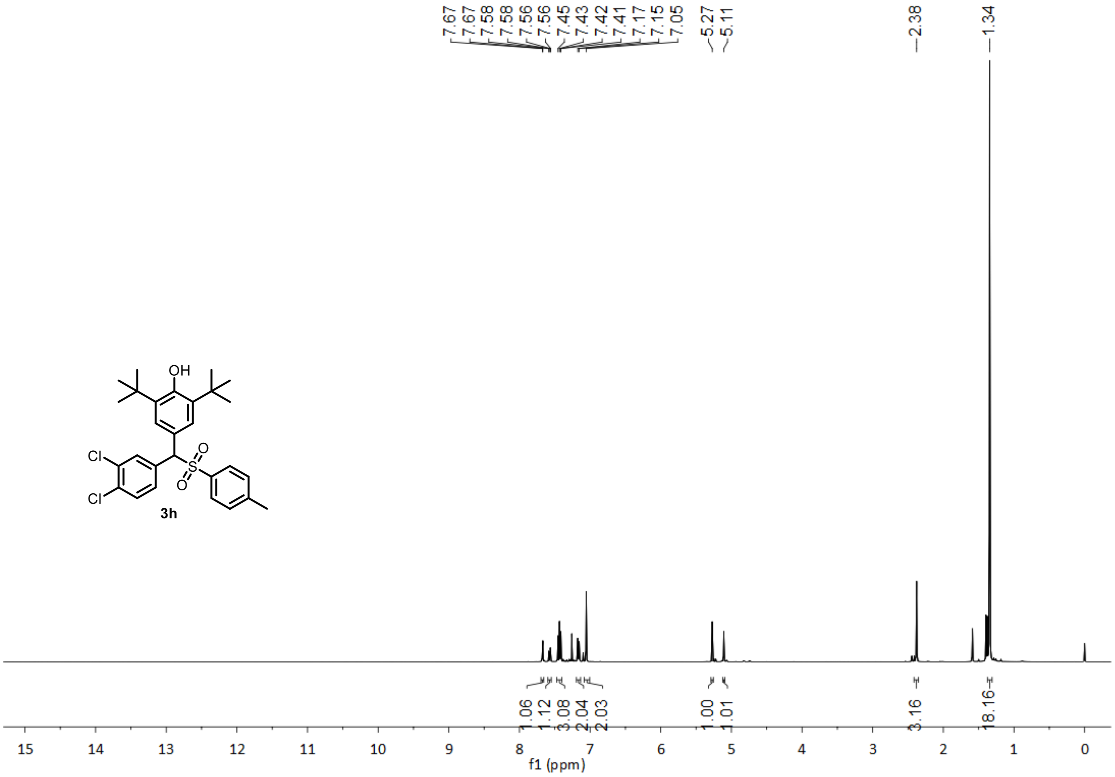

^{13}C NMR (100 MHz, CDCl$_3$):

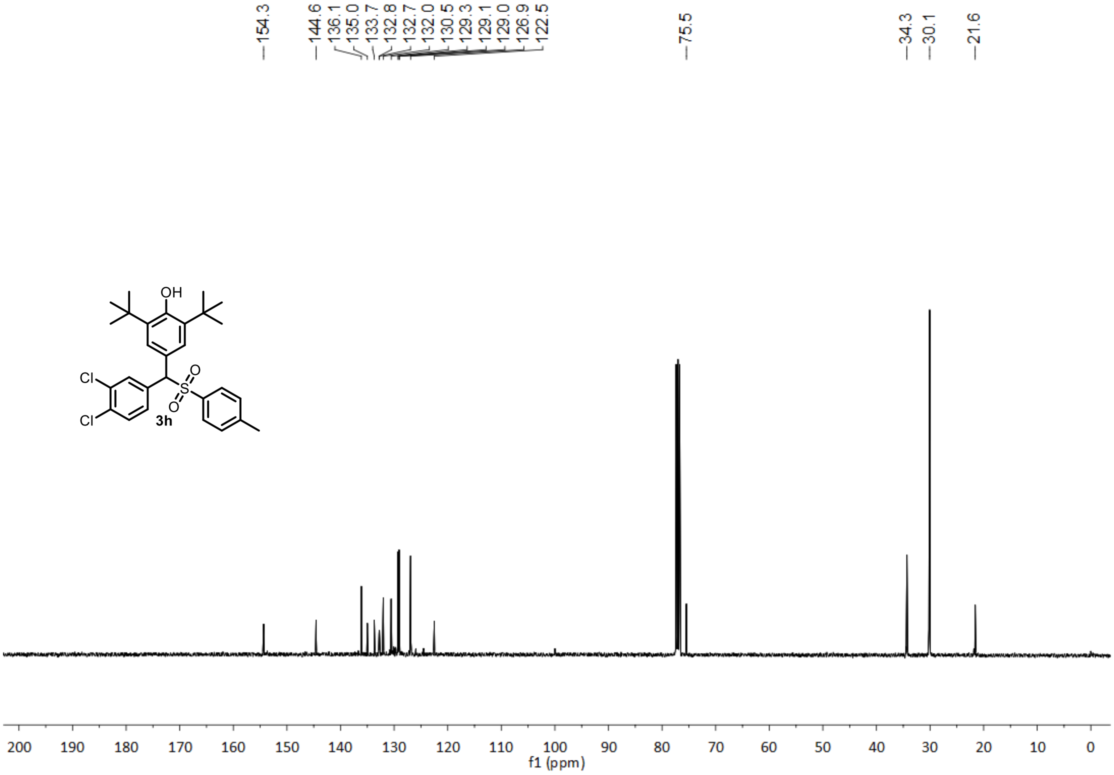

2,6-Di-*tert*-butyl-4-(tosyl(4-(trifluoromethyl)phenyl)methyl)phenol (**3i**)

^{1}H NMR (400 MHz, CDCl$_3$)

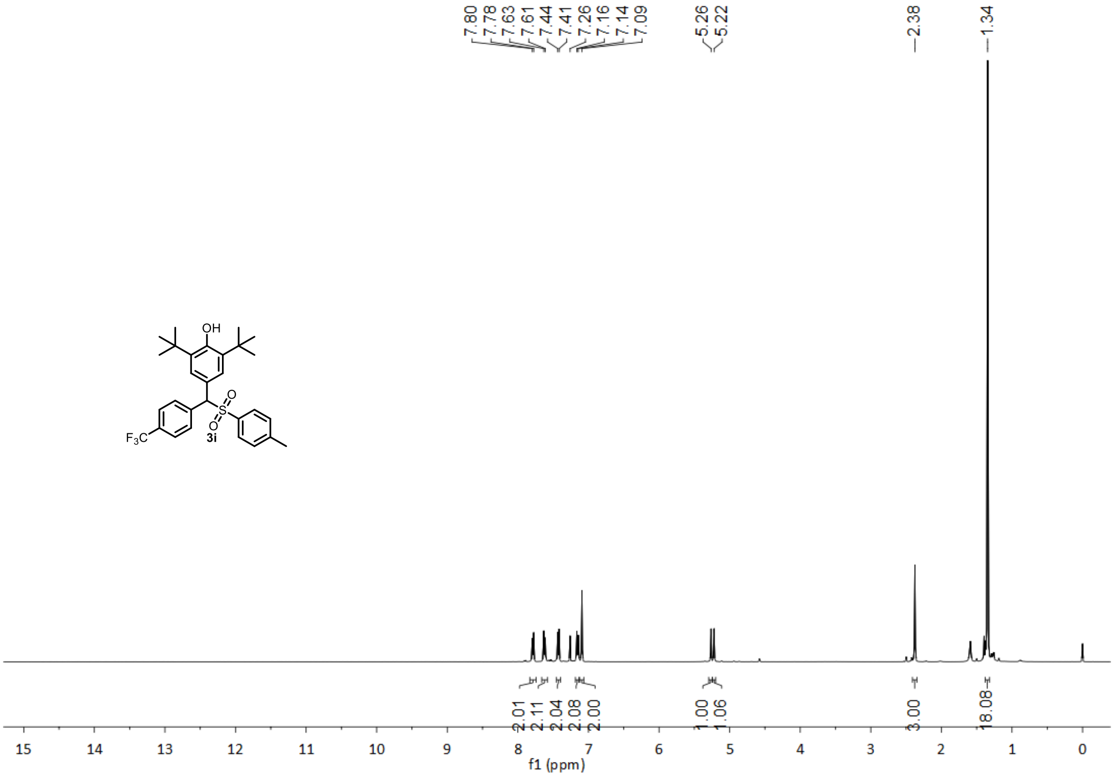

^{13}C NMR (100 MHz, CDCl$_3$):

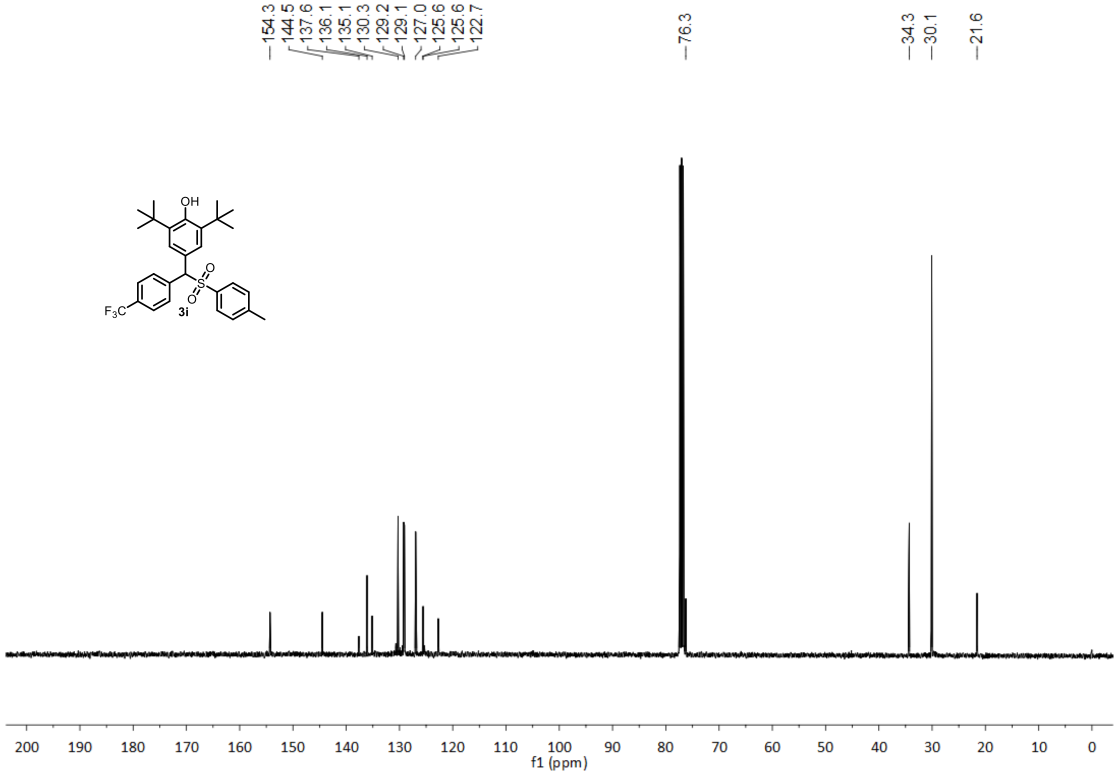

^{19}F NMR (376 MHz, CDCl$_3$):

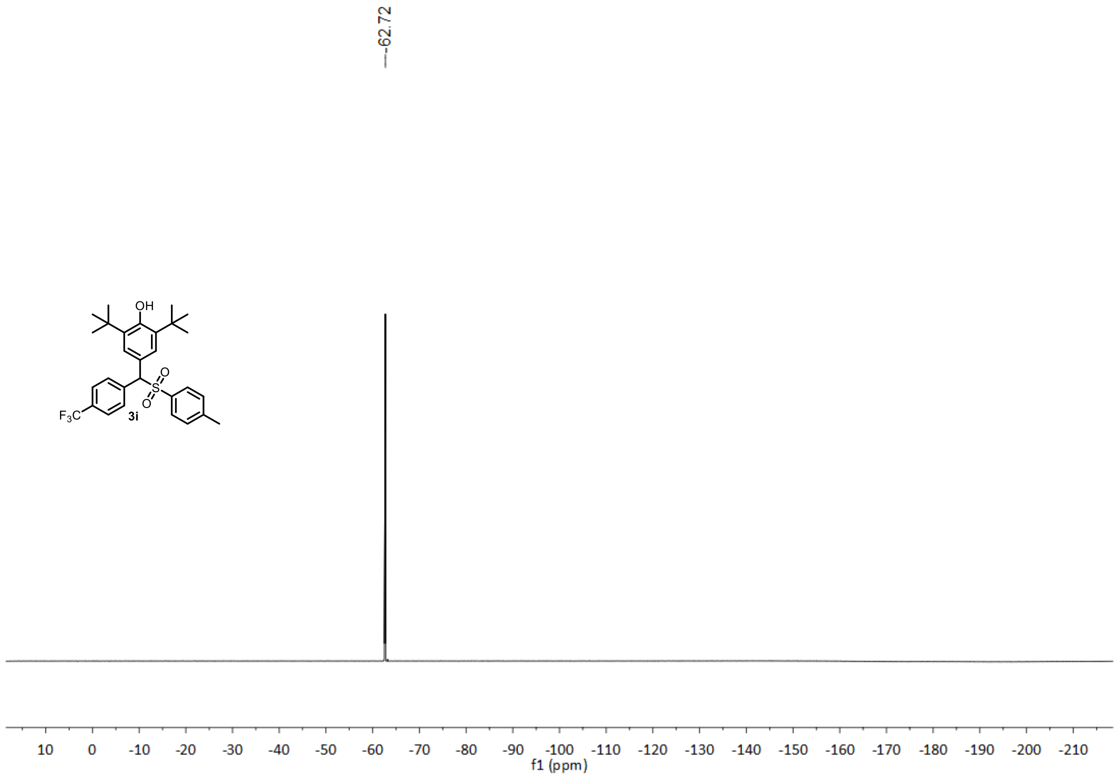

DEPT135

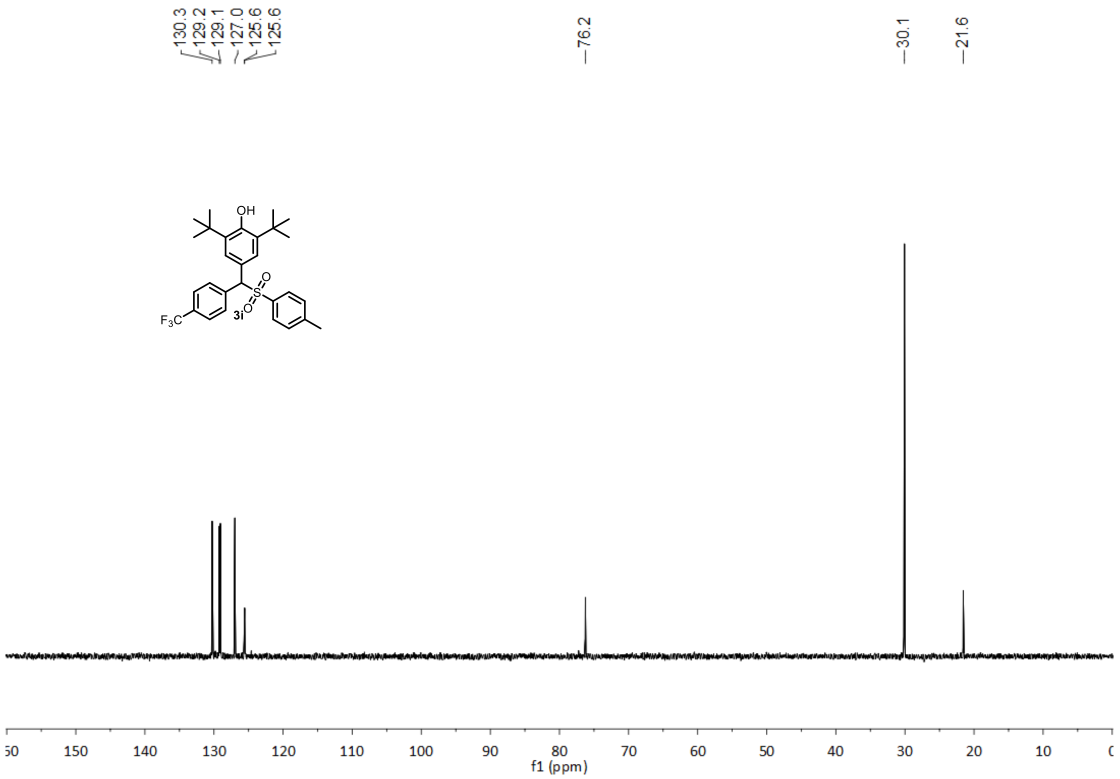

2,6-Di-*tert*-butyl-4-(tosyl(3,4,5-trimethoxyphenyl)methyl)phenol (**3j**)

^{1}H NMR (400 MHz, $CDCl_3$)

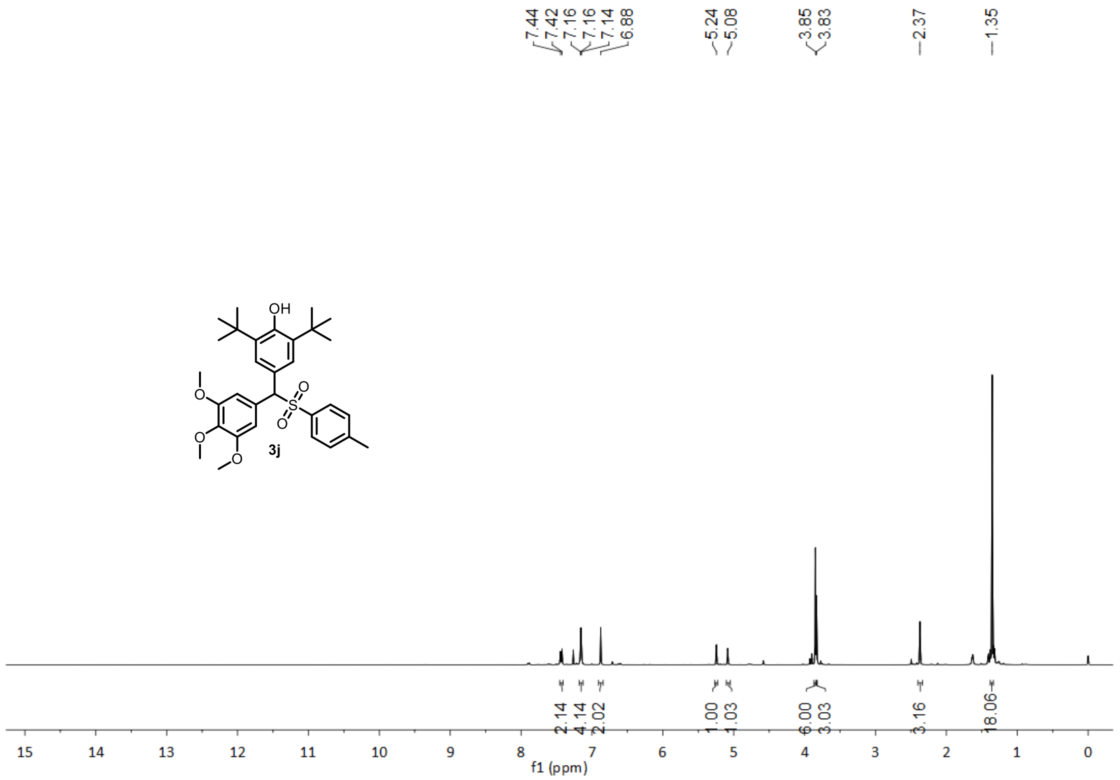

^{13}C NMR (100 MHz, CDCl$_3$):

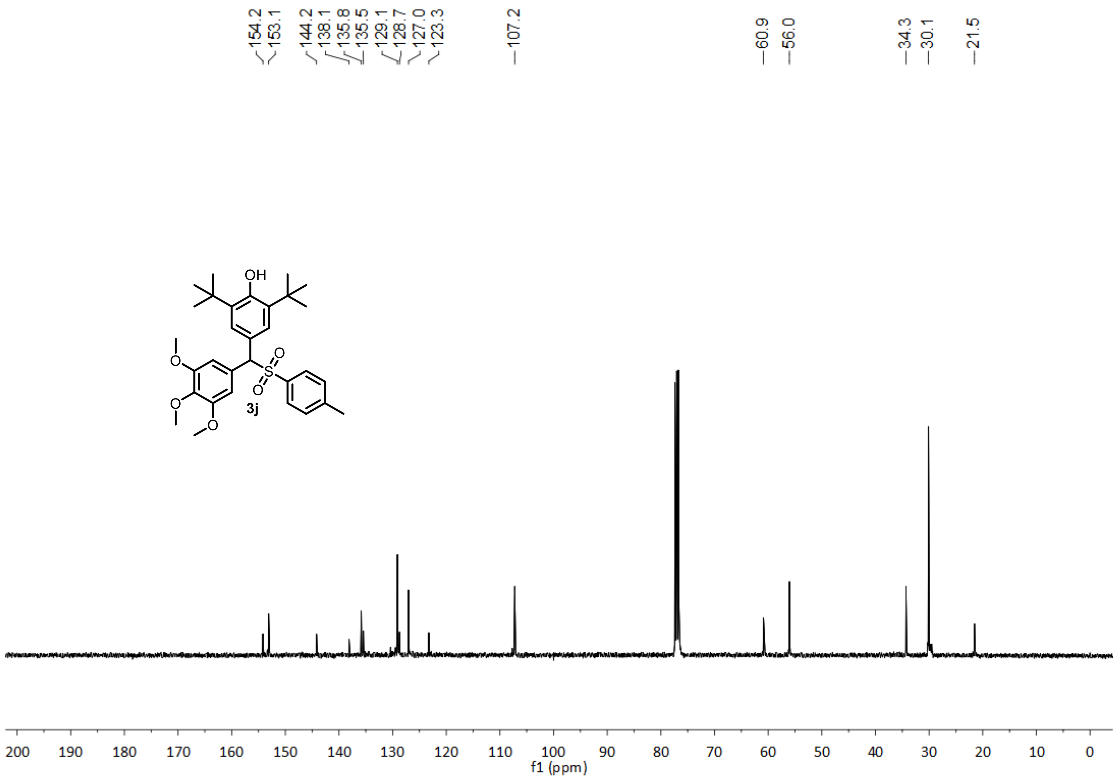

2,6-Di-*tert*-butyl-4-((3,4-dimethylphenyl)(tosyl)methyl)phenol (**3k**)

^{1}H NMR (400 MHz, CDCl$_3$)

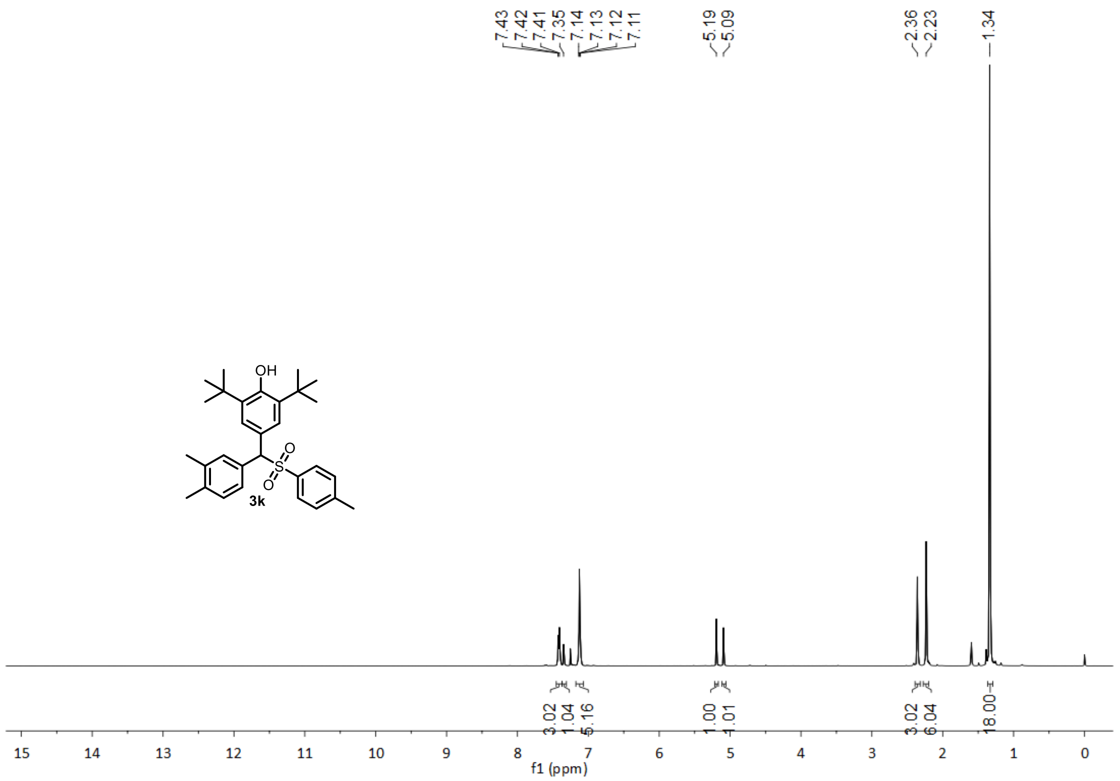

^{13}C NMR (100 MHz, $CDCl_3$):

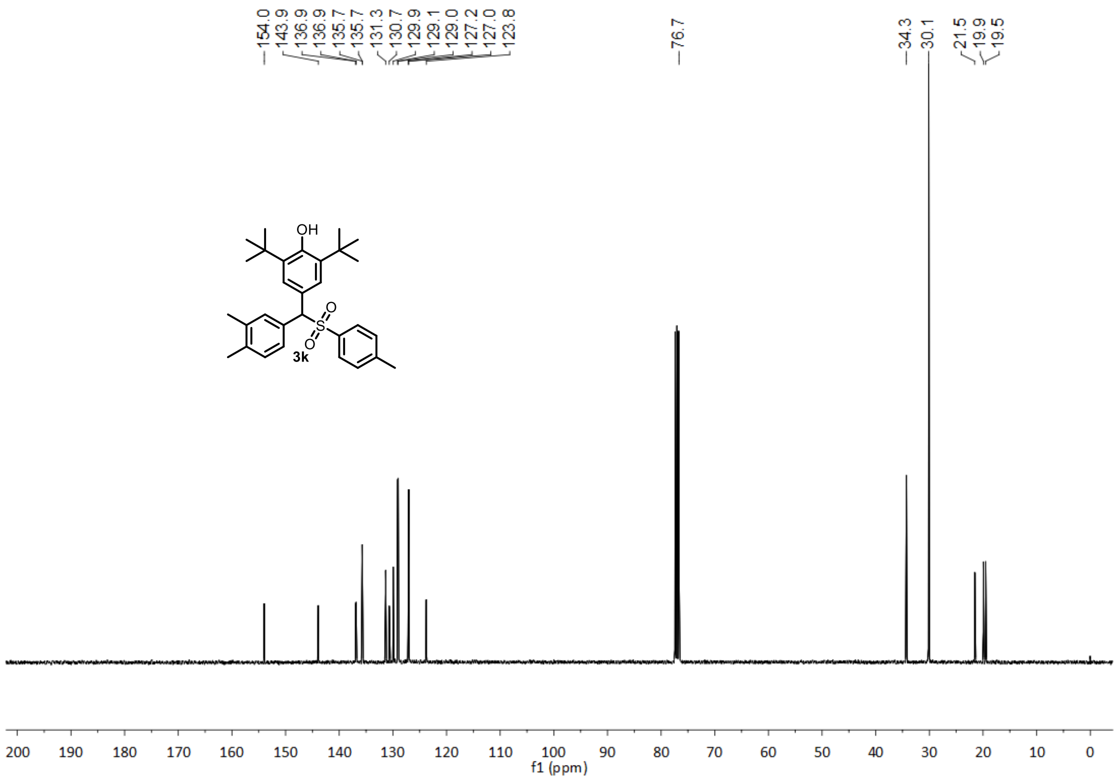

2,6-Di-*tert*-butyl-4-(naphthalen-2-yl(tosyl)methyl)phenol (**3l**)

^{1}H NMR (400 MHz, $CDCl_3$)

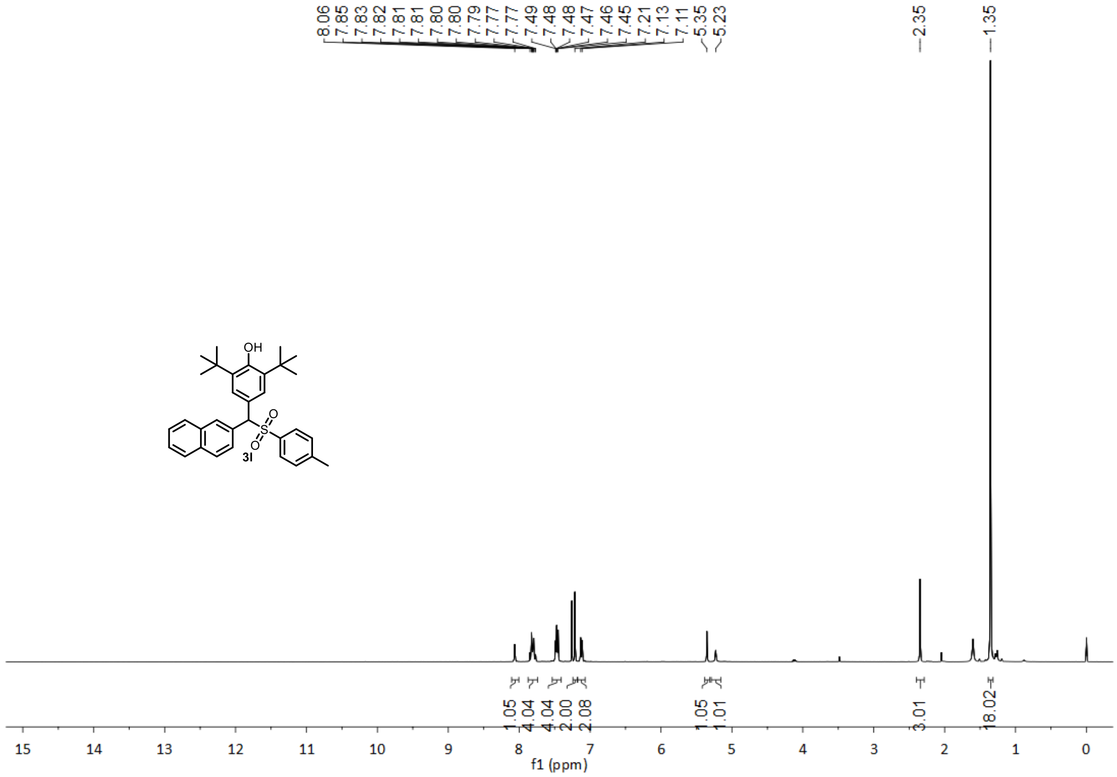

^{13}C NMR (100 MHz, CDCl$_3$):

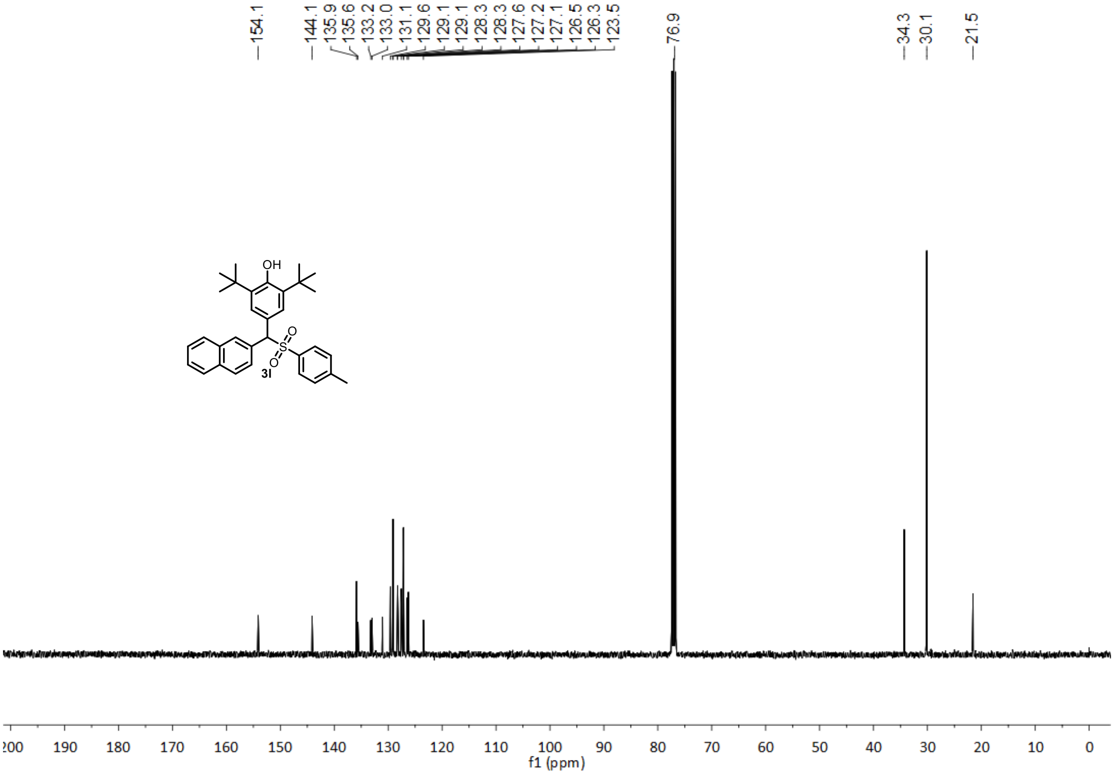

2,6-Di-*tert*-butyl-4-(thiophen-2-yl(tosyl)methyl)phenol (**3m**)

^{1}H NMR (400 MHz, DMSO-d_6)

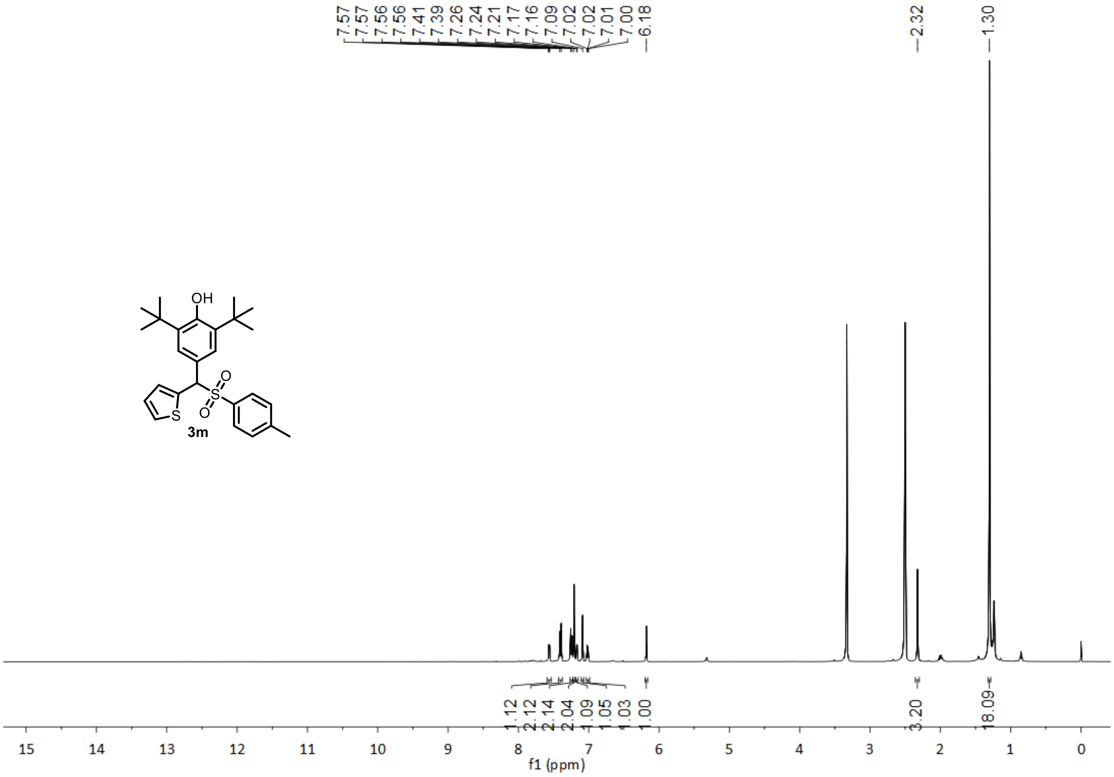

^{13}C NMR (100 MHz, DMSO-d_6):

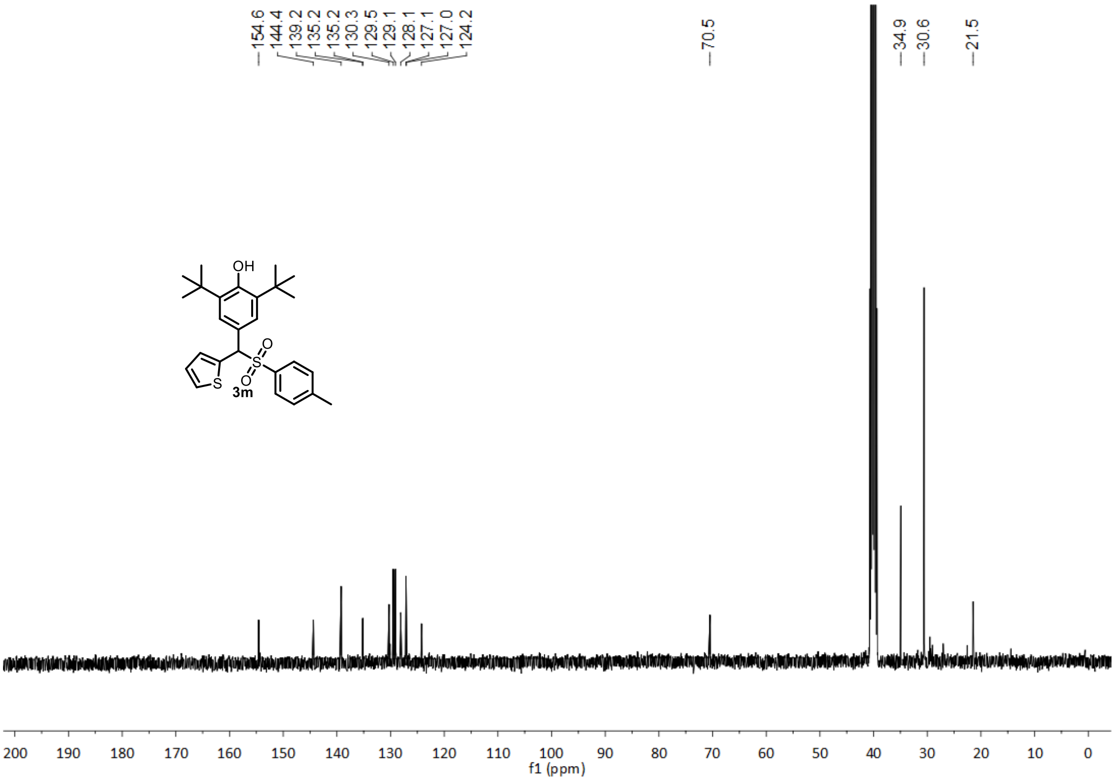

(±)-2,6-Di-*tert*-butyl-4-(2-isocyano-1-phenyl-2-tosylethyl)phenol (**±4a**)

^{1}H NMR (400 MHz, CDCl$_3$):

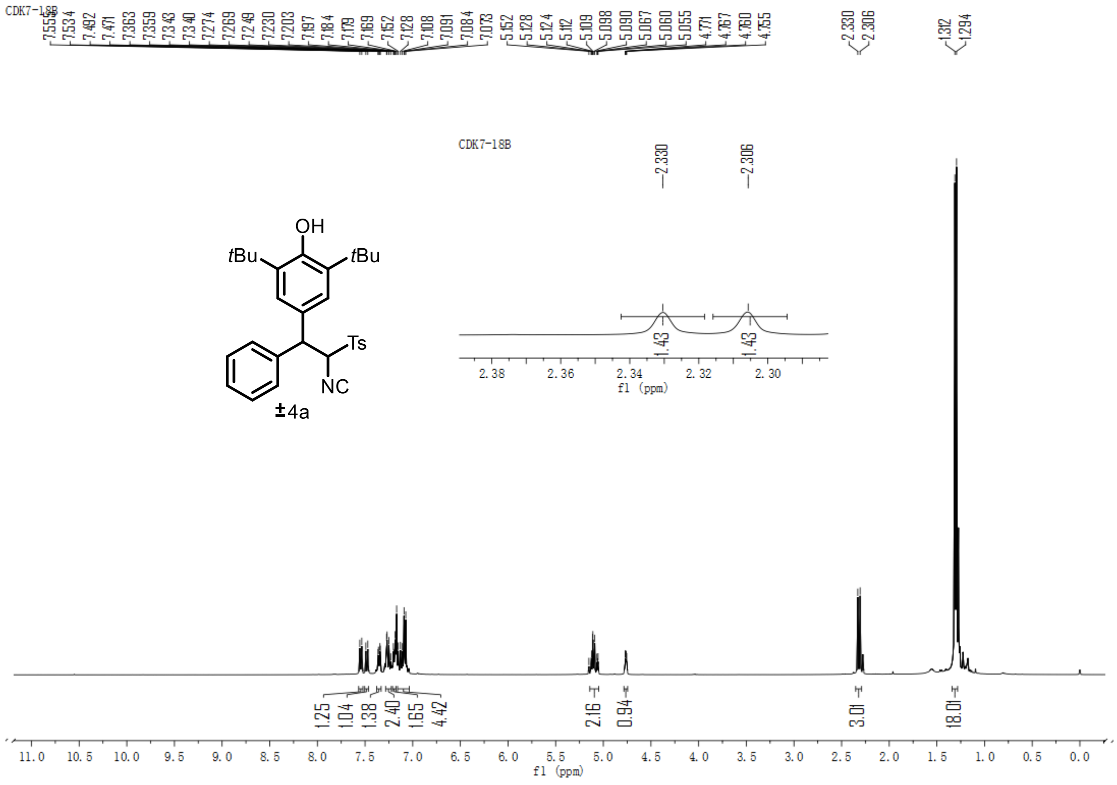

^{13}C NMR (100 MHz, CDCl$_3$):

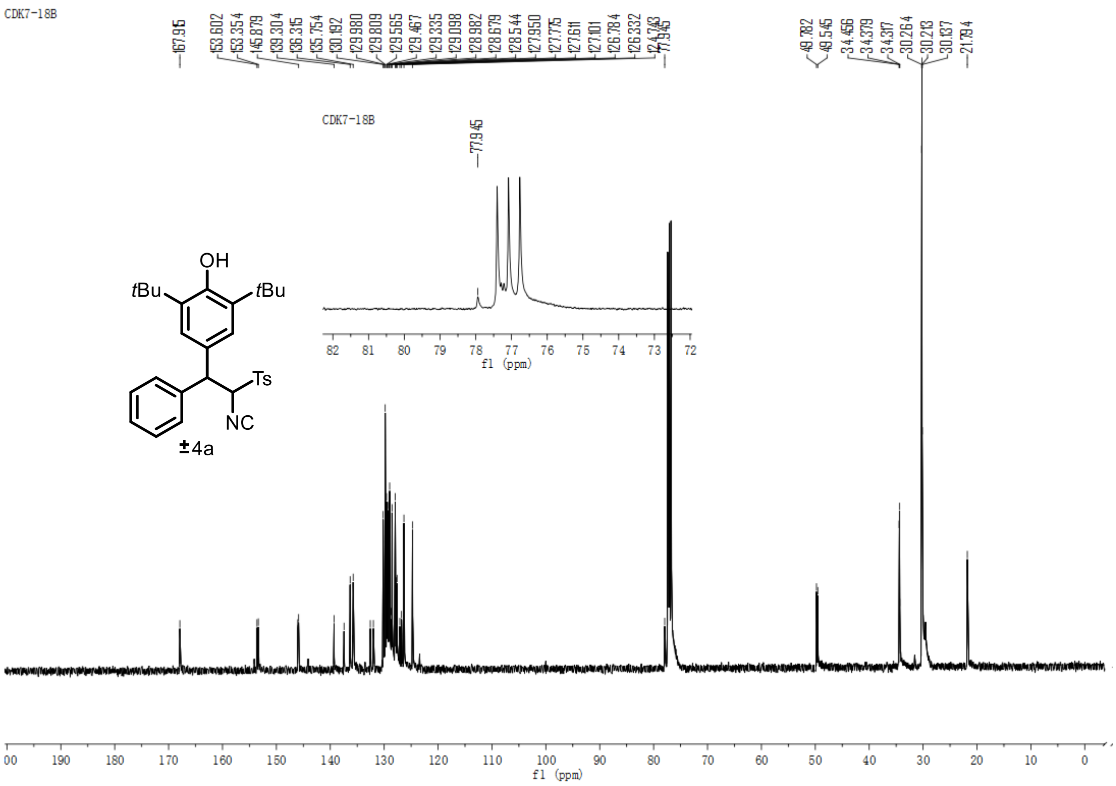

2,6-Di-*tert*-butyl-4-(2-isocyano-1-phenyl-2-tosylethyl)phenol (**4a-up**)

^{1}H NMR (400 MHz, CDCl$_3$):

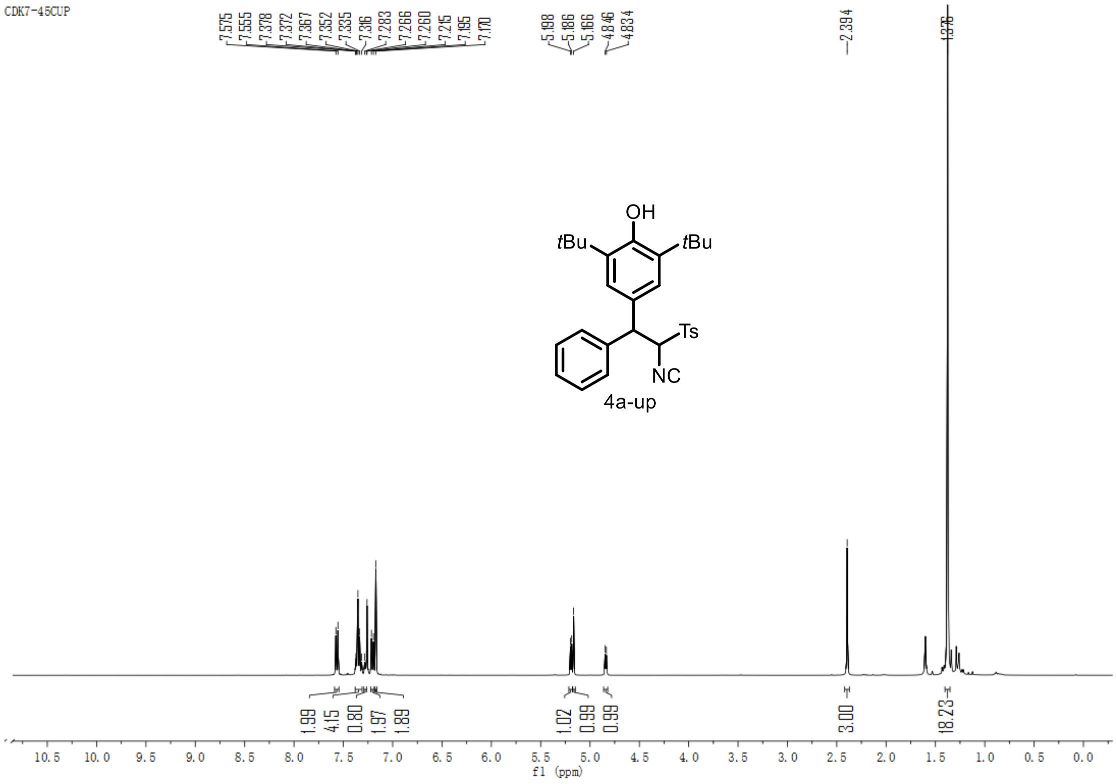

^{13}C NMR (100 MHz, CDCl$_3$):

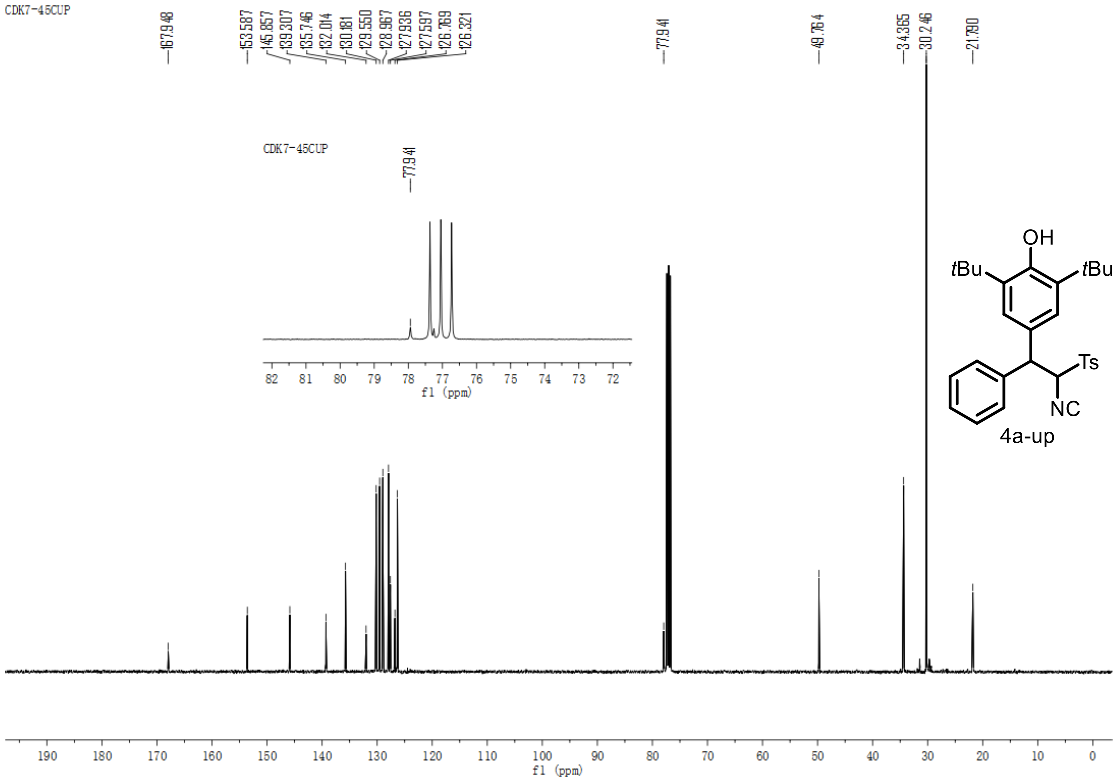

2,6-Di-*tert*-butyl-4-(2-isocyano-1-phenyl-2-tosylethyl)phenol (**4a-down**)

^{1}H NMR (400 MHz, CDCl$_3$):

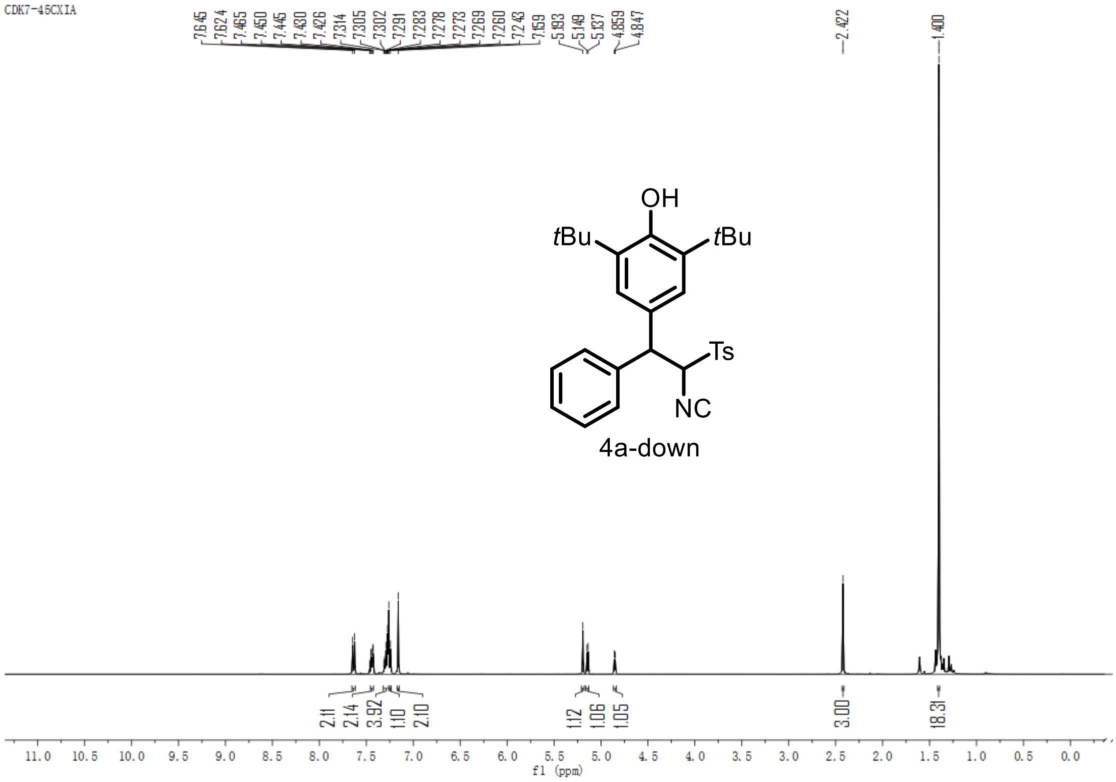

^{13}C NMR (100 MHz, CDCl$_3$):

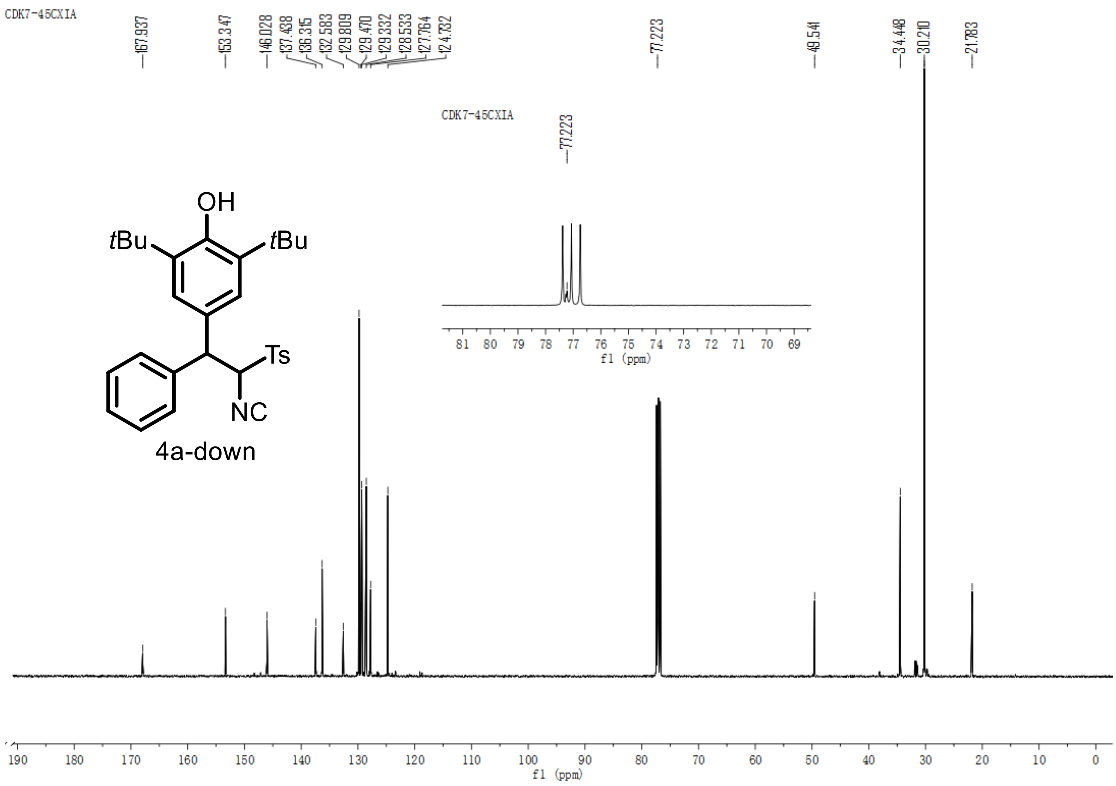

(±)-2,6-Di-*tert*-butyl-4-(2-isocyano-1-(*p*-tolyl)-2-tosylethyl)phenol (**±4b**)

^{1}H NMR (400 MHz, CDCl$_3$):

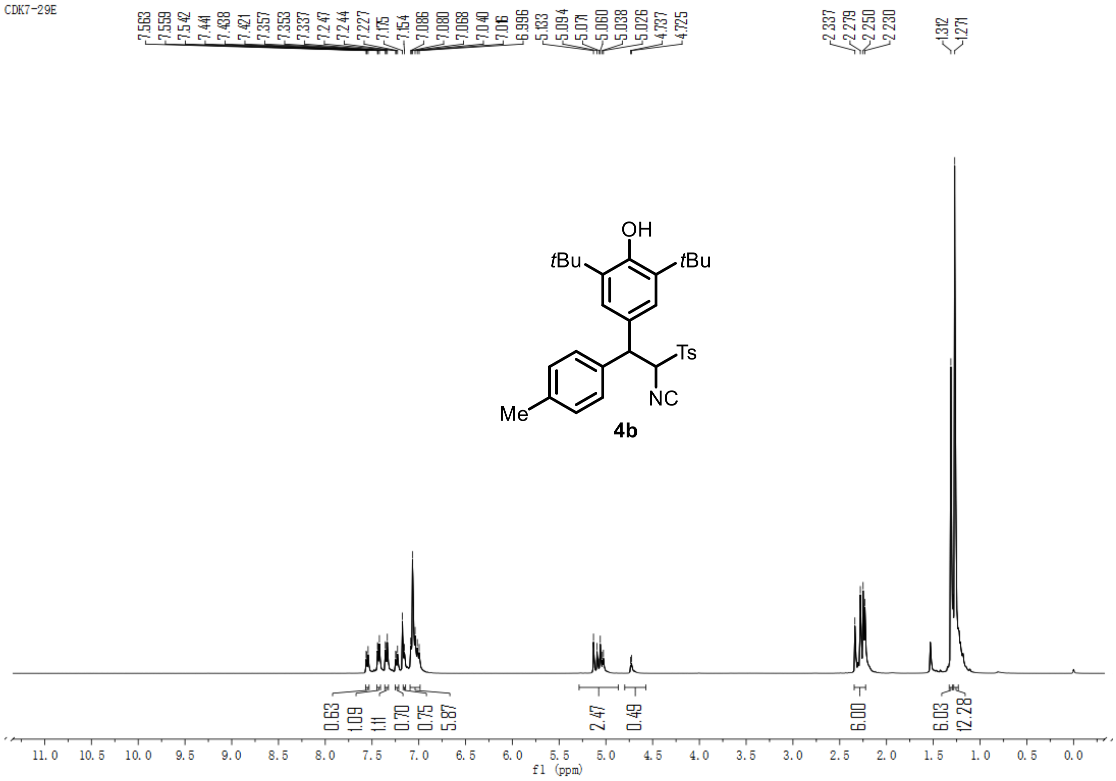

^{13}C NMR (100 MHz, CDCl$_3$):

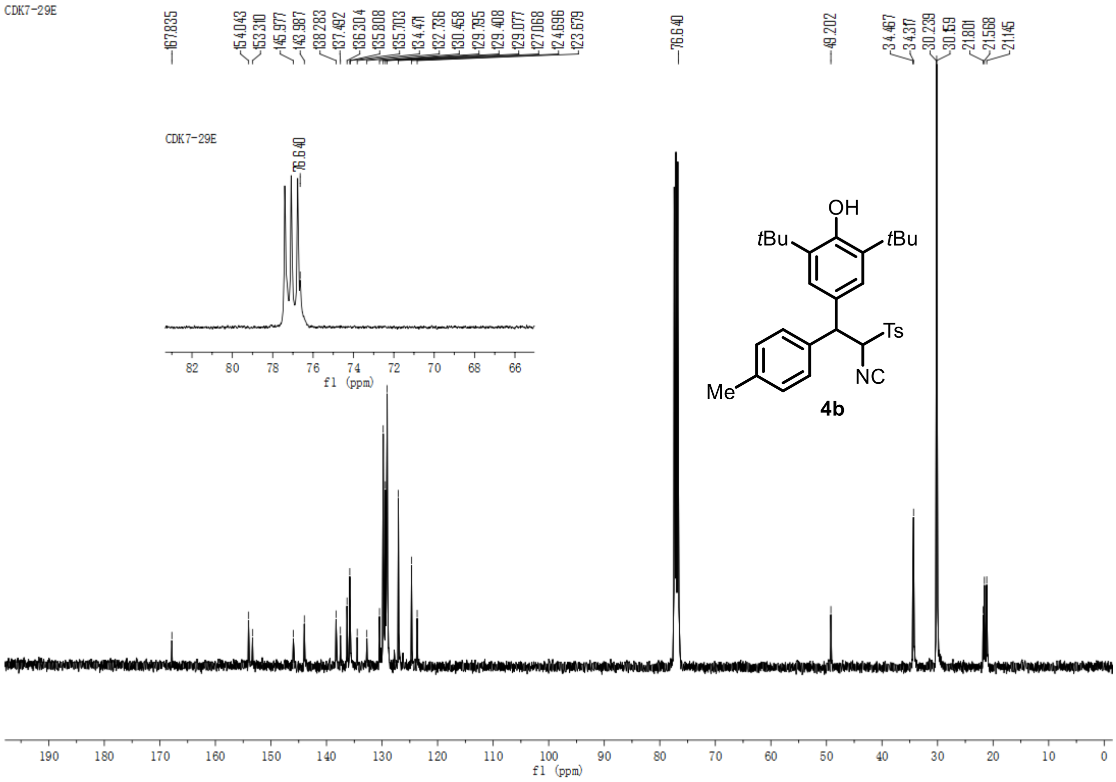

(±)-2,6-Di-*tert*-butyl-4-1-(4-(*tert*-butyl)phenyl)-2-isocyano-2-tosylethyl)phenol (**±4c**)

^{1}H NMR (400 MHz, CDCl$_3$):

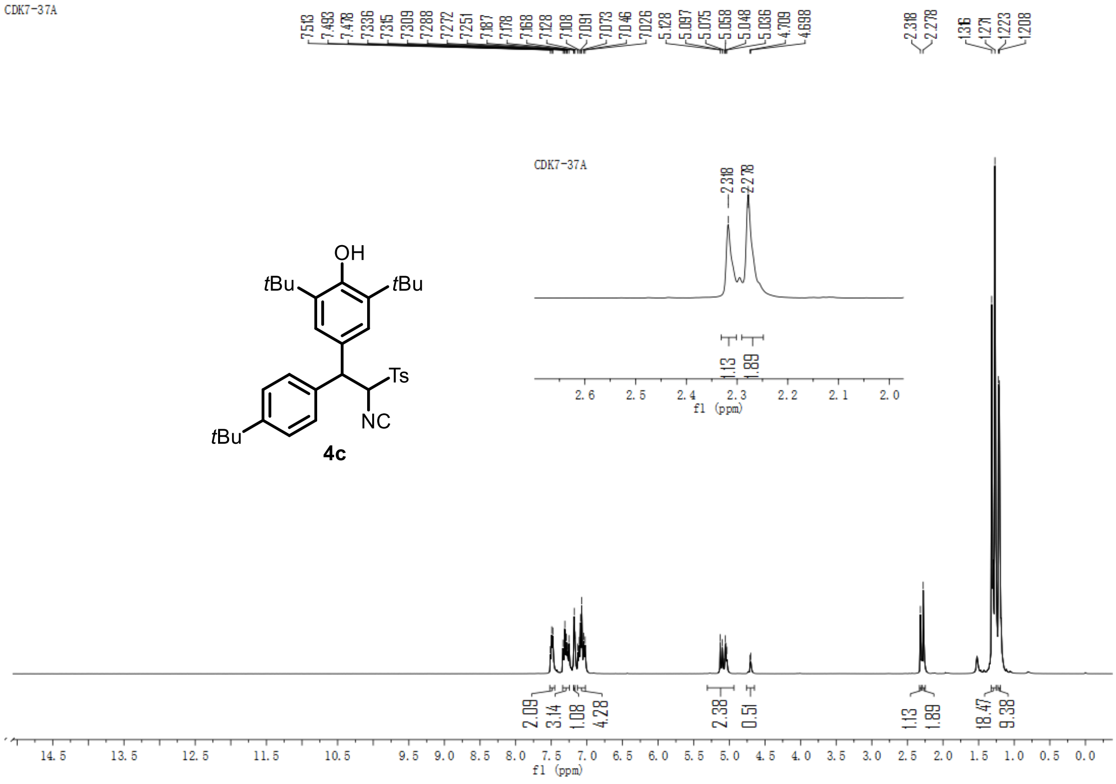

^{13}C NMR (100 MHz, CDCl₃):

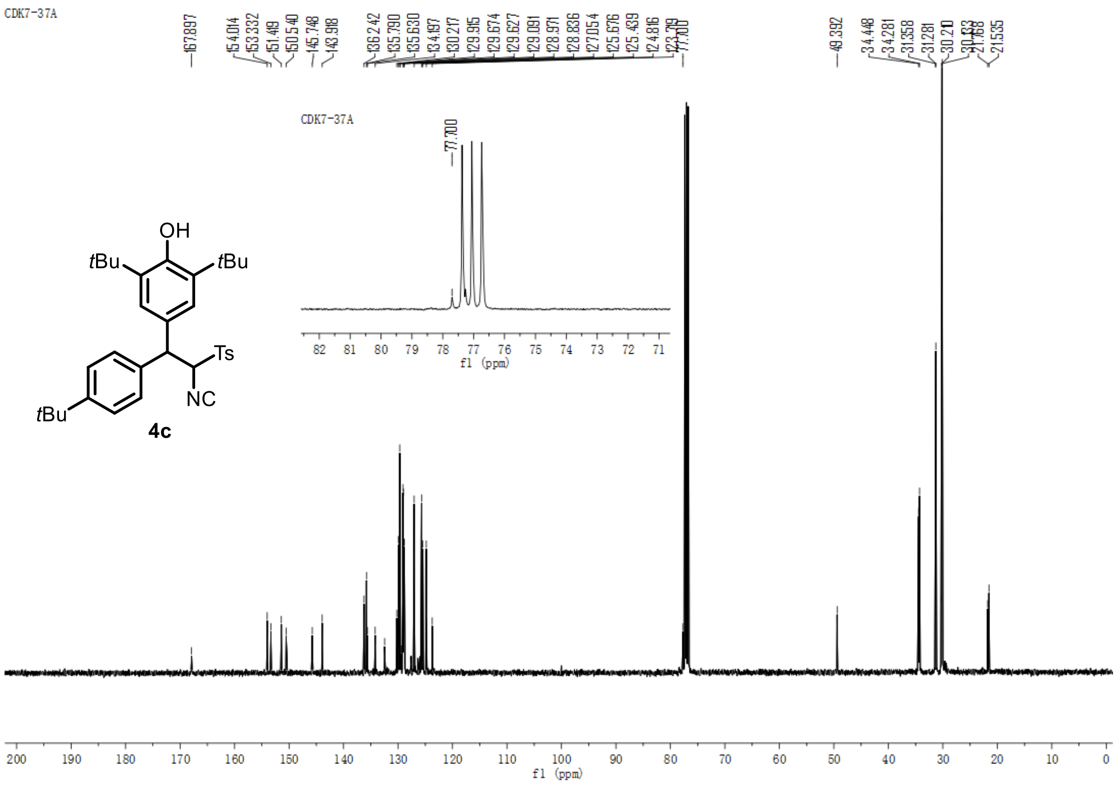

(±)-2,6-Di-*tert*-butyl-4-(1-(4-fluorophenyl)-2-isocyano-2-tosylethyl)phenol (**±4d**)

^{1}H NMR (400 MHz, CDCl₃)

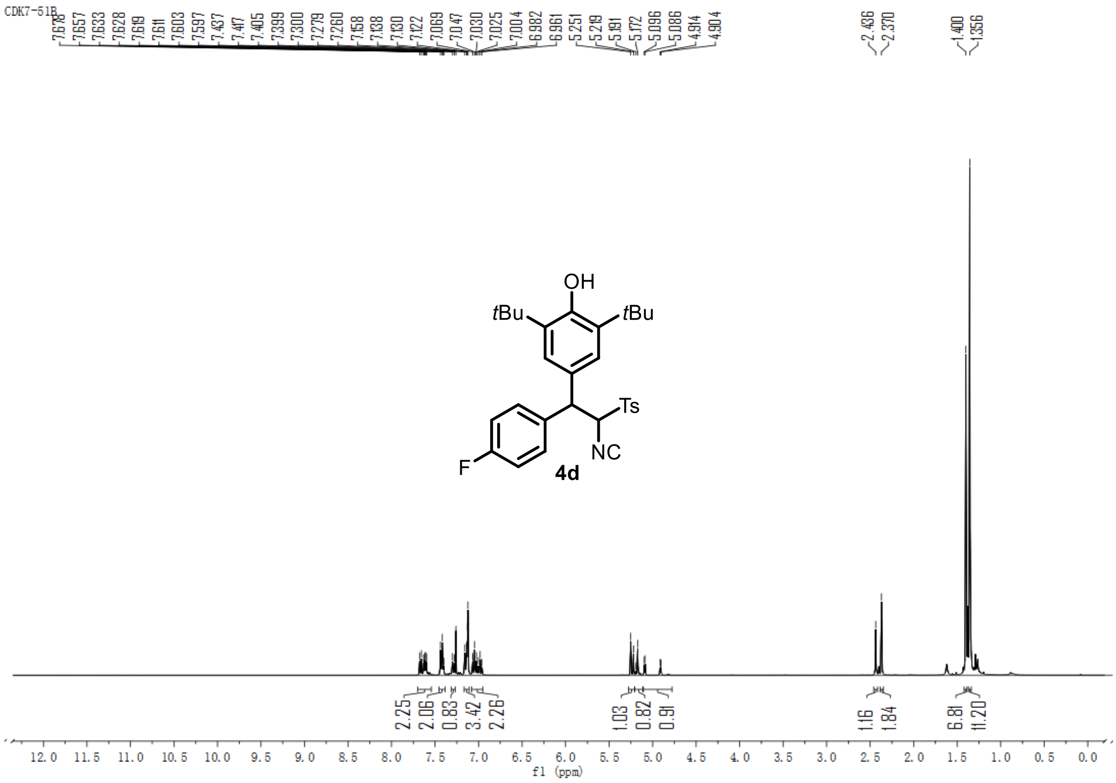

^{13}C NMR (100 MHz, $CDCl_3$):

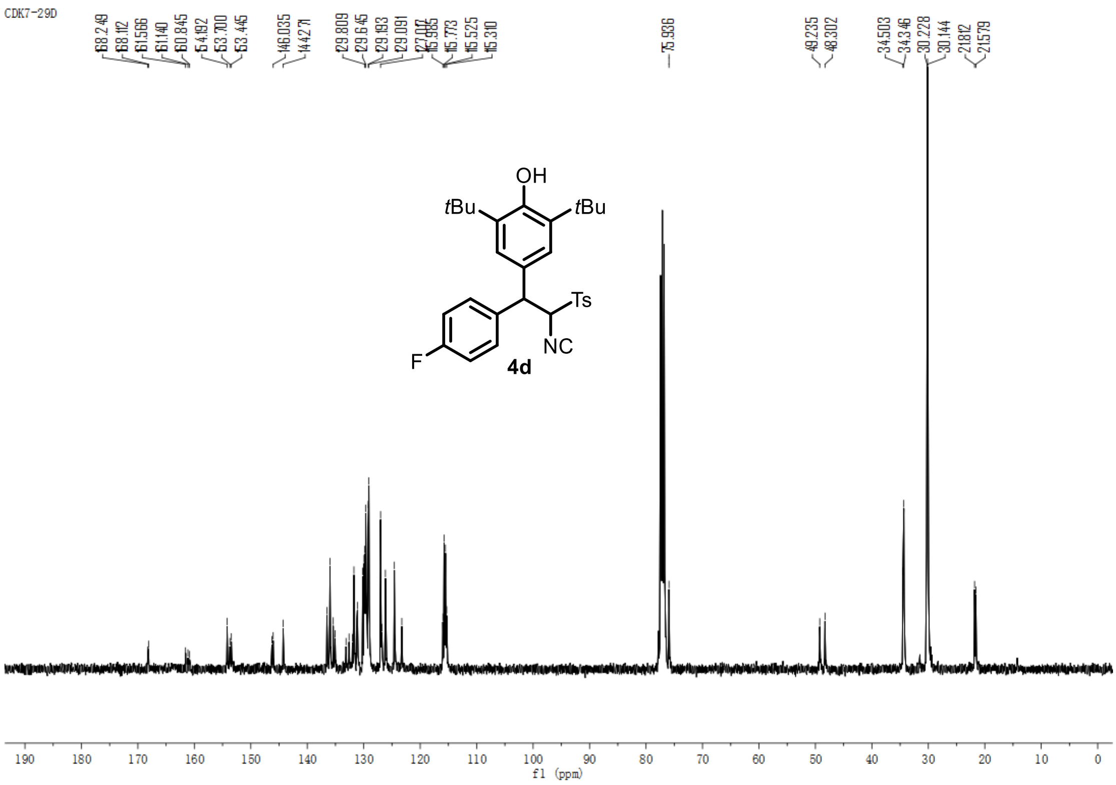

^{19}F NMR (376 MHz, $CDCl_3$):

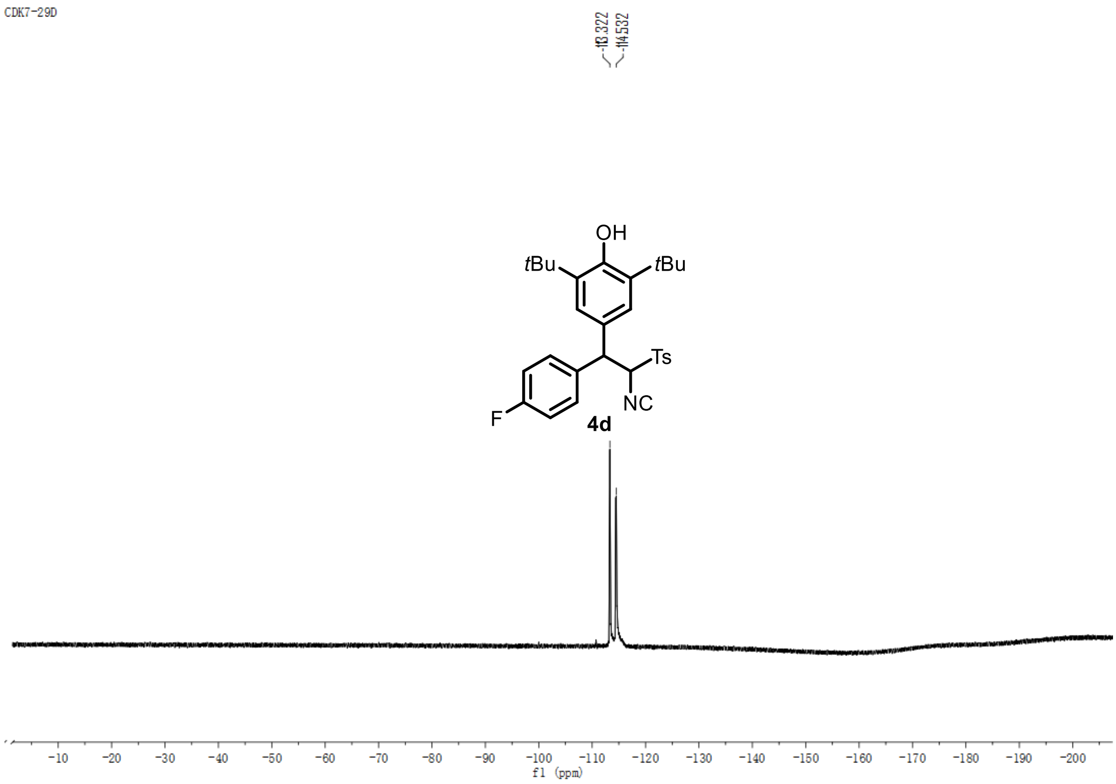

(±)-4-(1-(4-Bromophenyl)-2-isocyano-2-tosylethyl)-2,6-di-*tert*-butylphenol (**±4e**)

^{1}H NMR (400 MHz, $CDCl_3$)

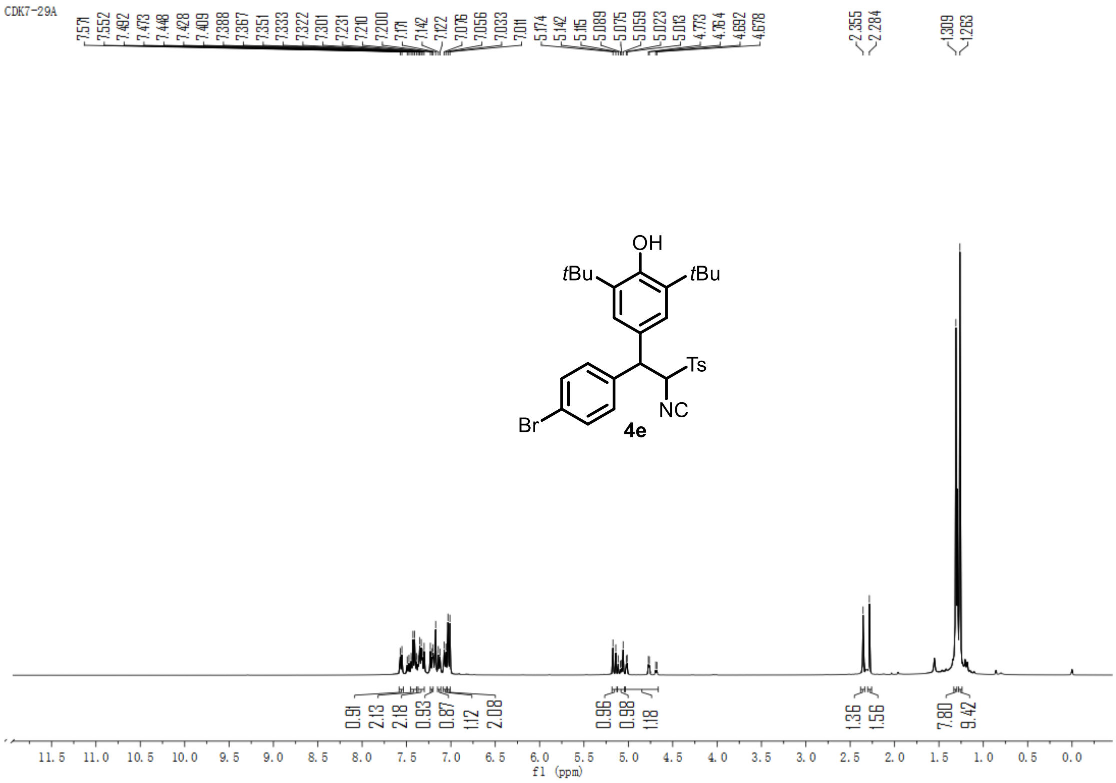

^{13}C NMR (100 MHz, $CDCl_3$):

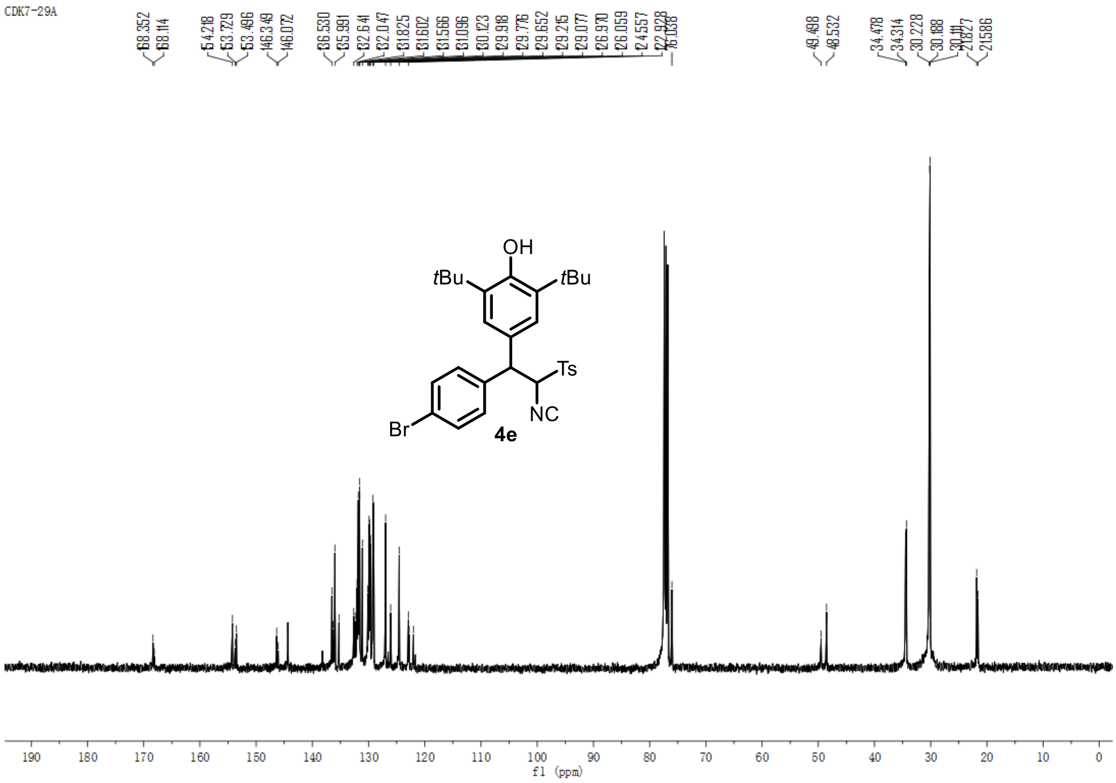

(±)-2,6-Di-*tert*-butyl-4-(2-isocyano-2-tosyl-1-(4-(trifluoromethyl)phenyl)ethyl)phenol (±**4f**)

^{1}H NMR (400 MHz, CDCl$_3$)

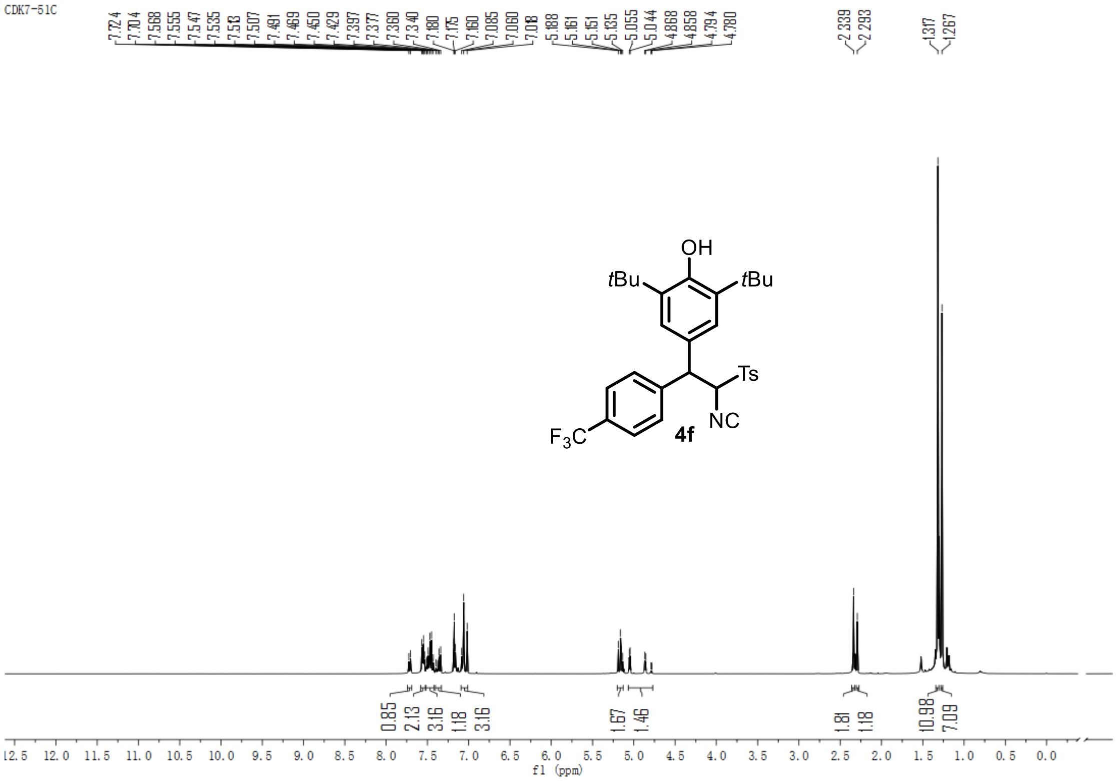

^{13}C NMR (100 MHz, CDCl$_3$):

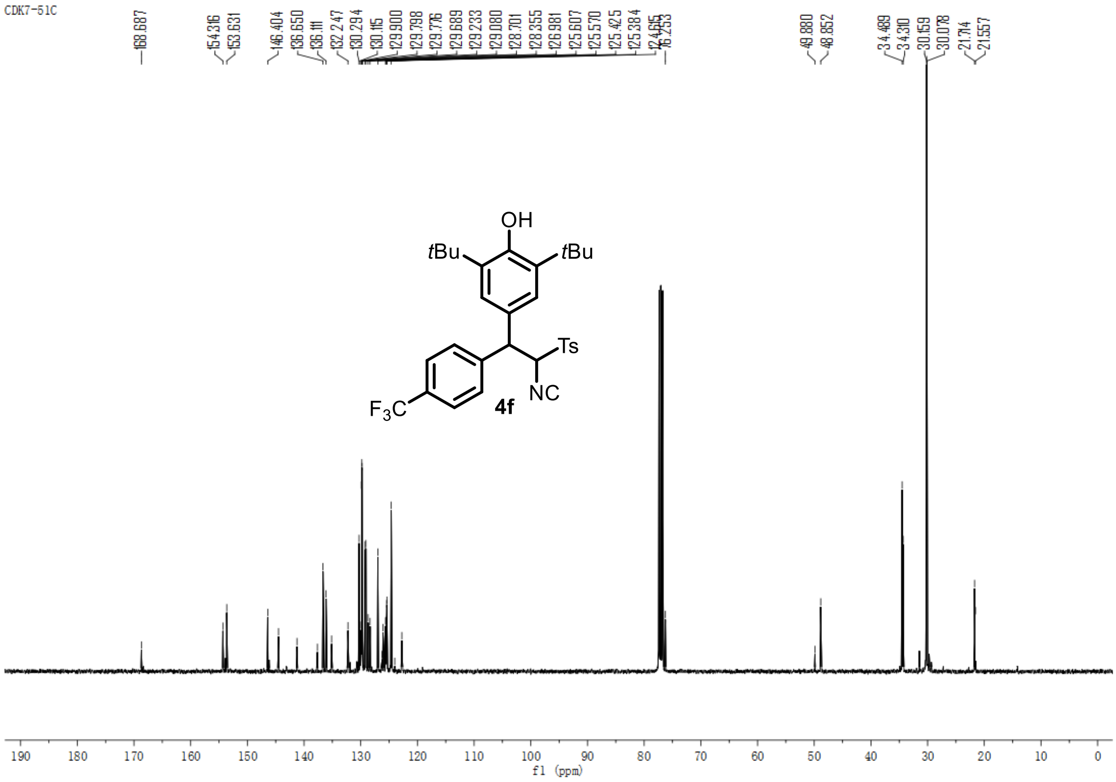

^{19}F NMR (376 MHz, CDCl$_3$):

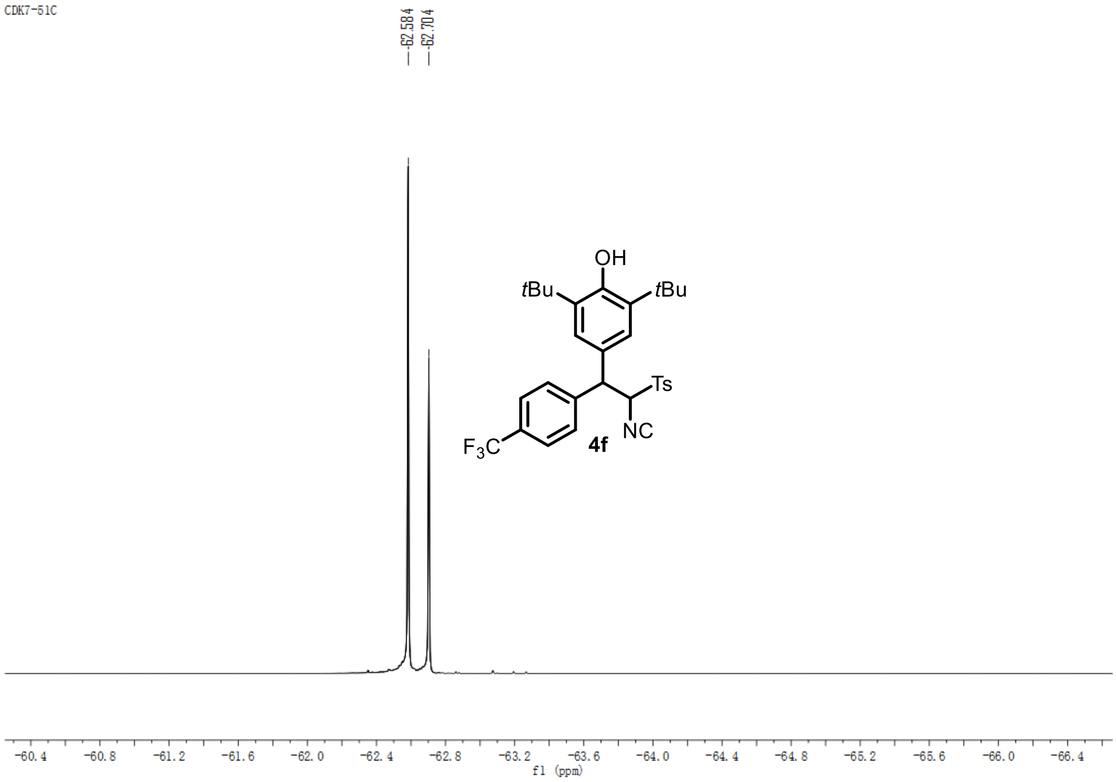

4-(1-(4-Bromothiophen-2-yl)-2-isocyano-2-tosylethyl)-2,6-di-*tert*-butylphenol (**4g**)

^{1}H NMR (400 MHz, CDCl$_3$)

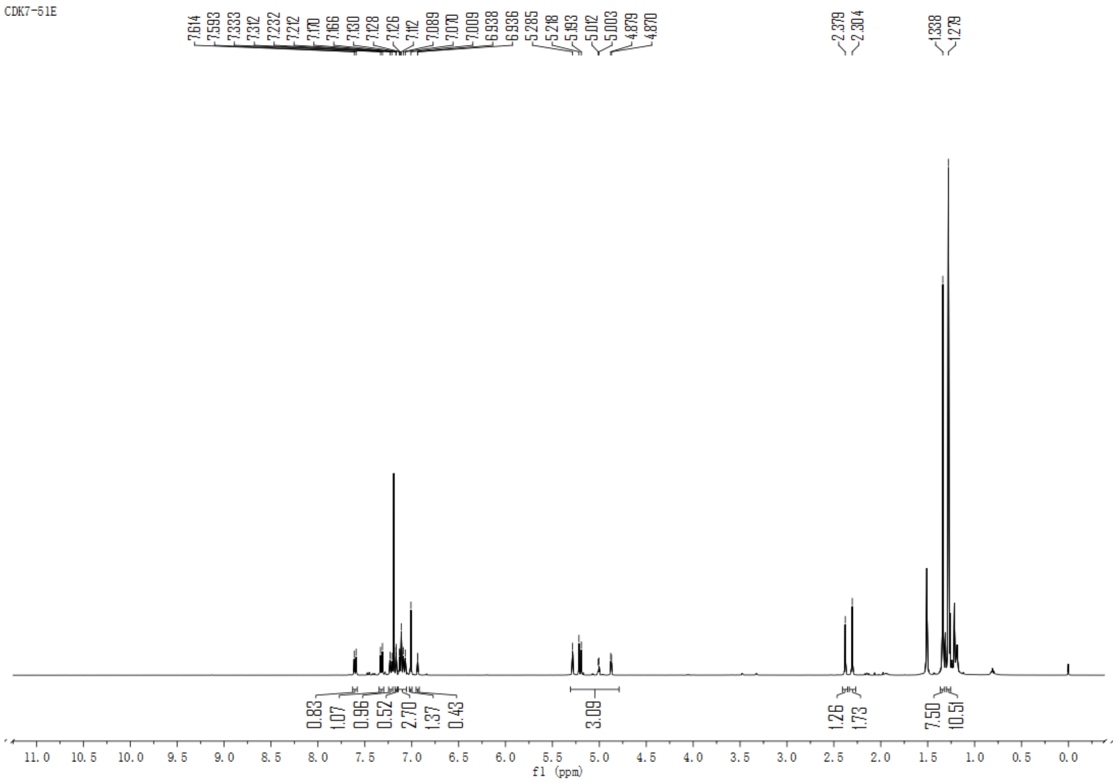

^{13}C NMR (100 MHz, CDCl$_3$):

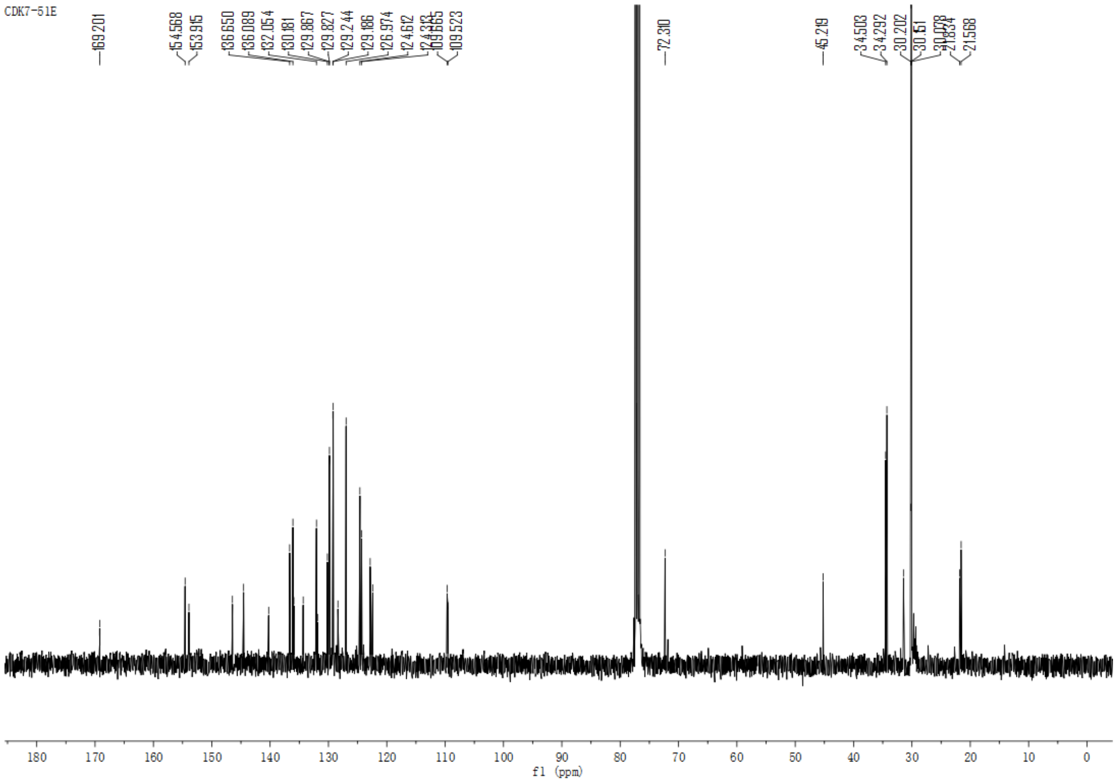

Methyl 3-(3,5-di-*tert*-butyl-4-hydroxyphenyl)-2-isocyano-3-phenylpropanoate (**4h**)

^{1}H NMR (400 MHz, CDCl$_3$)

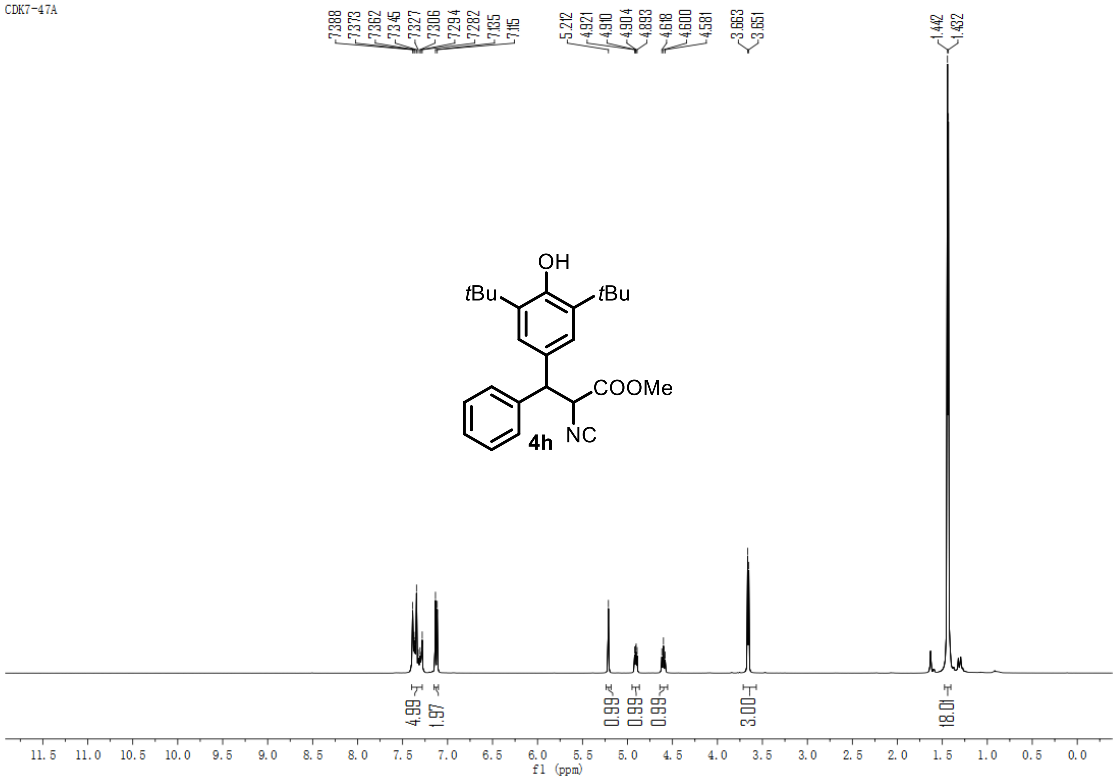

^{13}C NMR (100 MHz, $CDCl_3$):

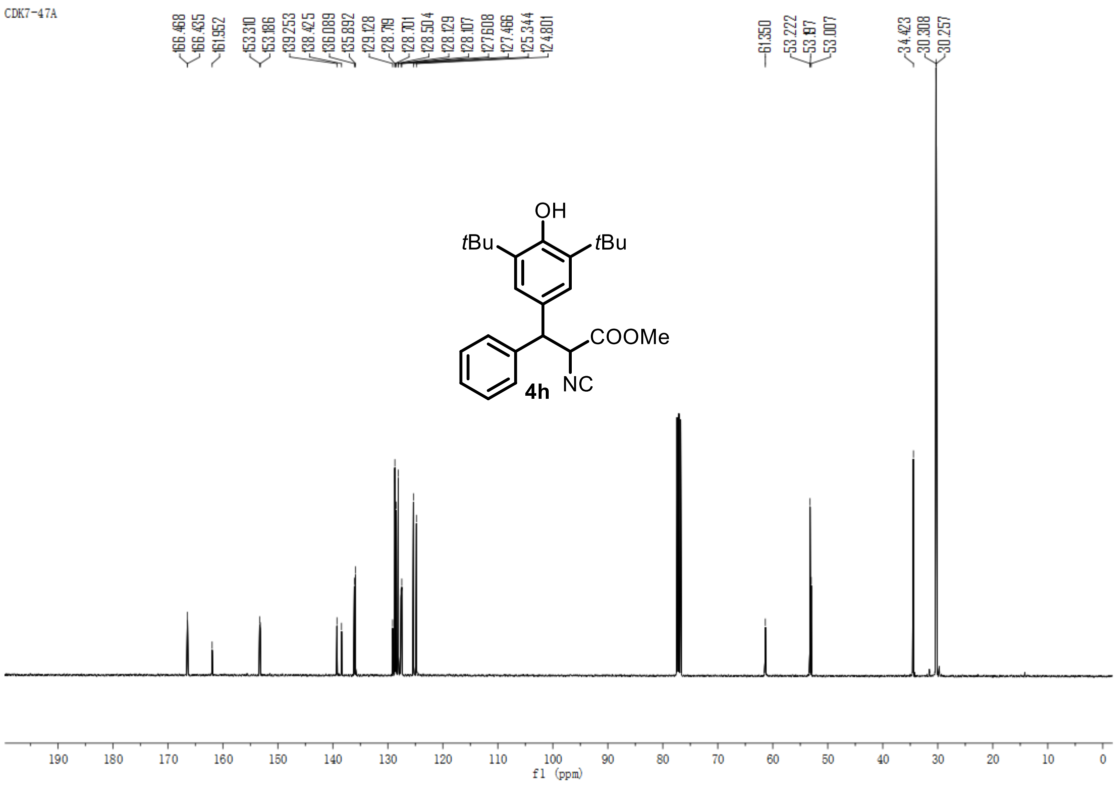

DEPT135

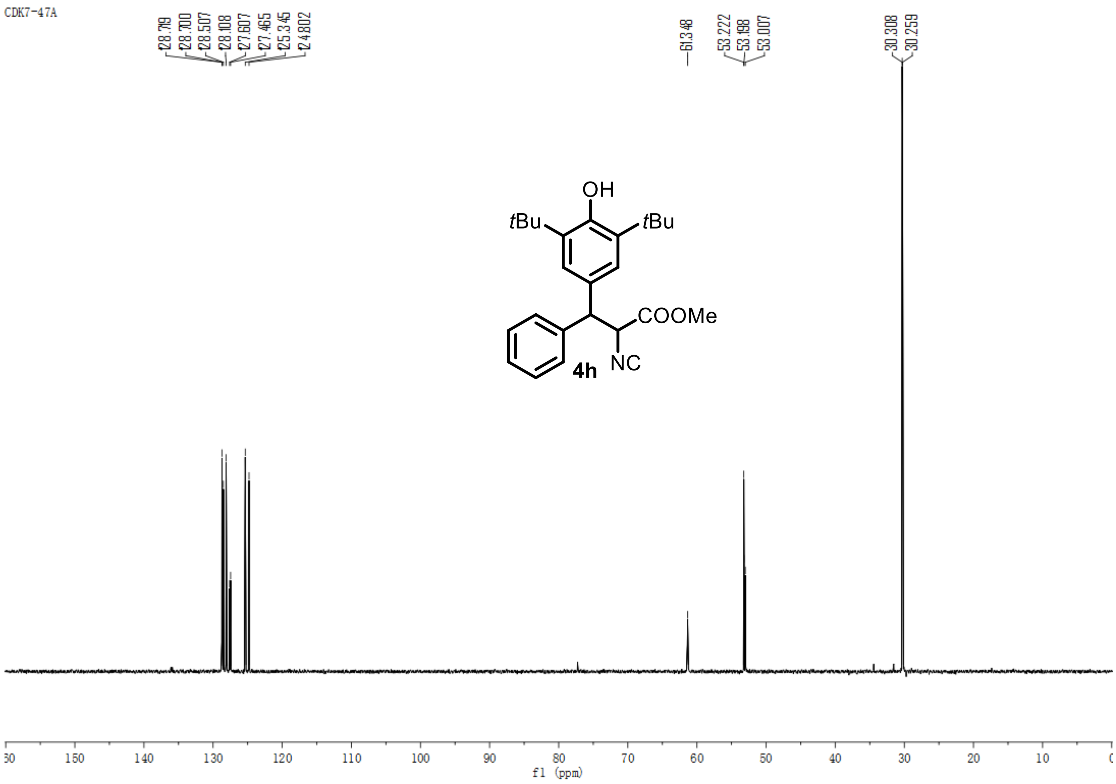

3-(3,5-Di-*tert*-butyl-4-hydroxyphenyl)-2,2-difluoro-1-(naphthalen-2-yl)-3-(*p*-tolyl)propan-1-one (**5**)

^{1}H NMR (400 MHz, CDCl$_3$):

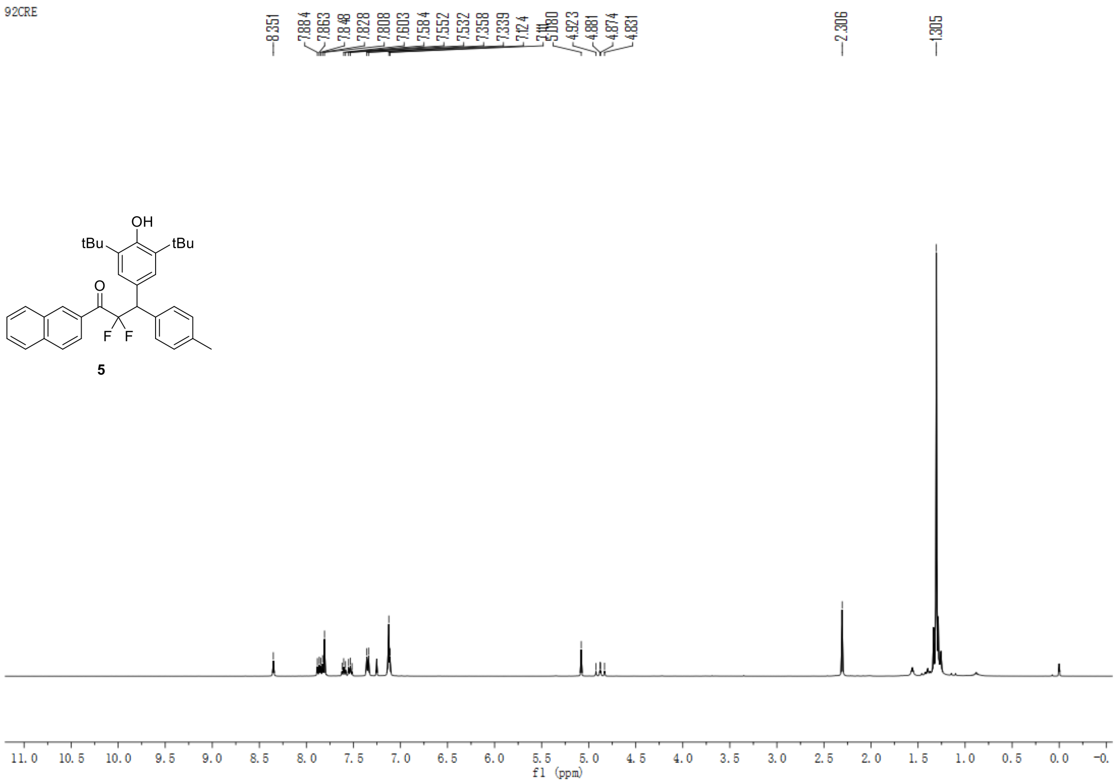

^{19}F NMR (376 MHz, CDCl$_3$):

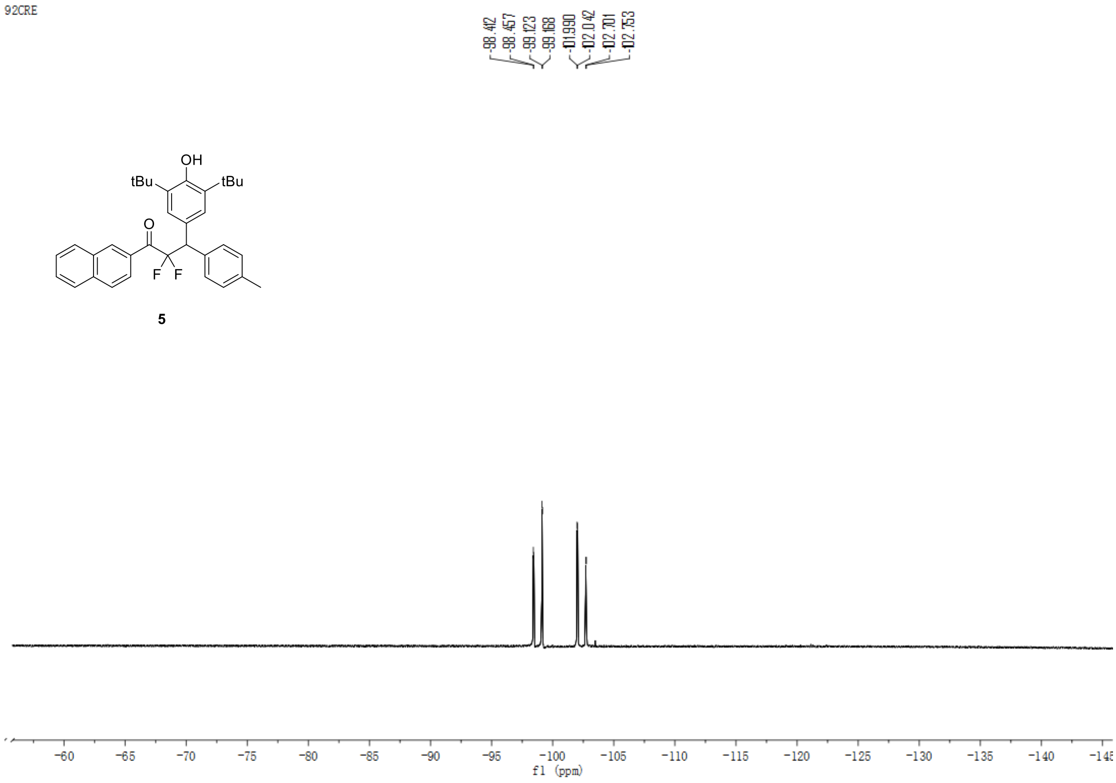

^{13}C NMR (100 MHz, $CDCl_3$):

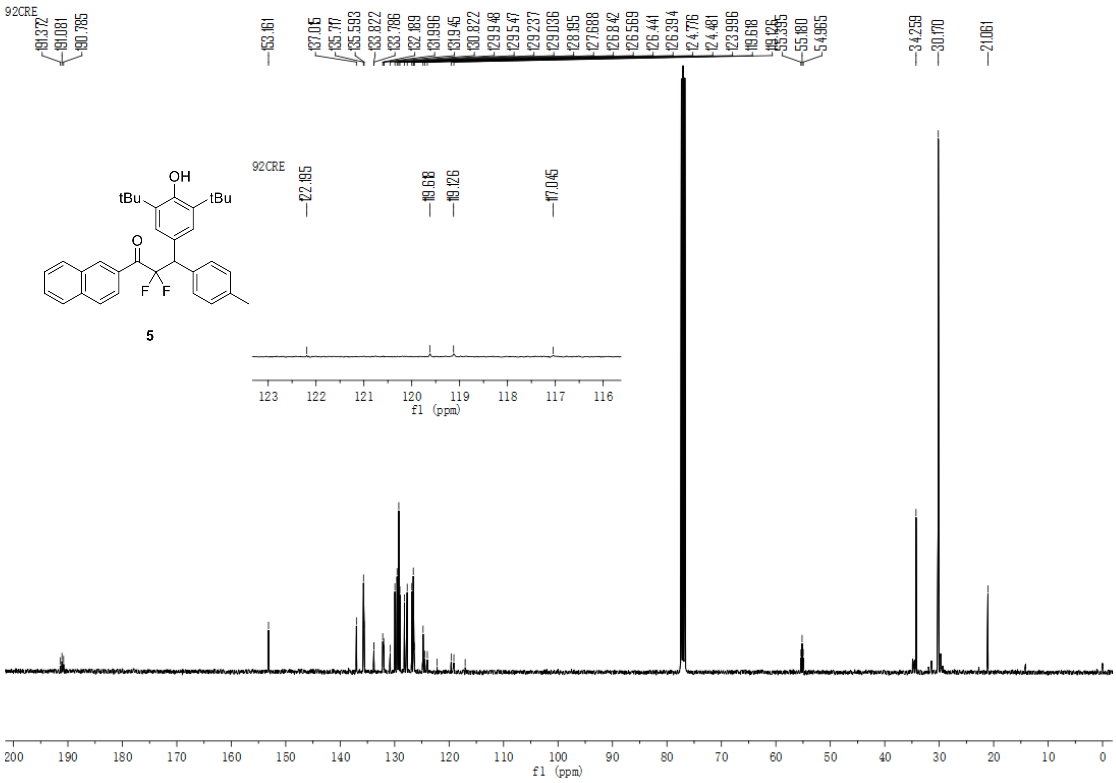

DEPT of 5

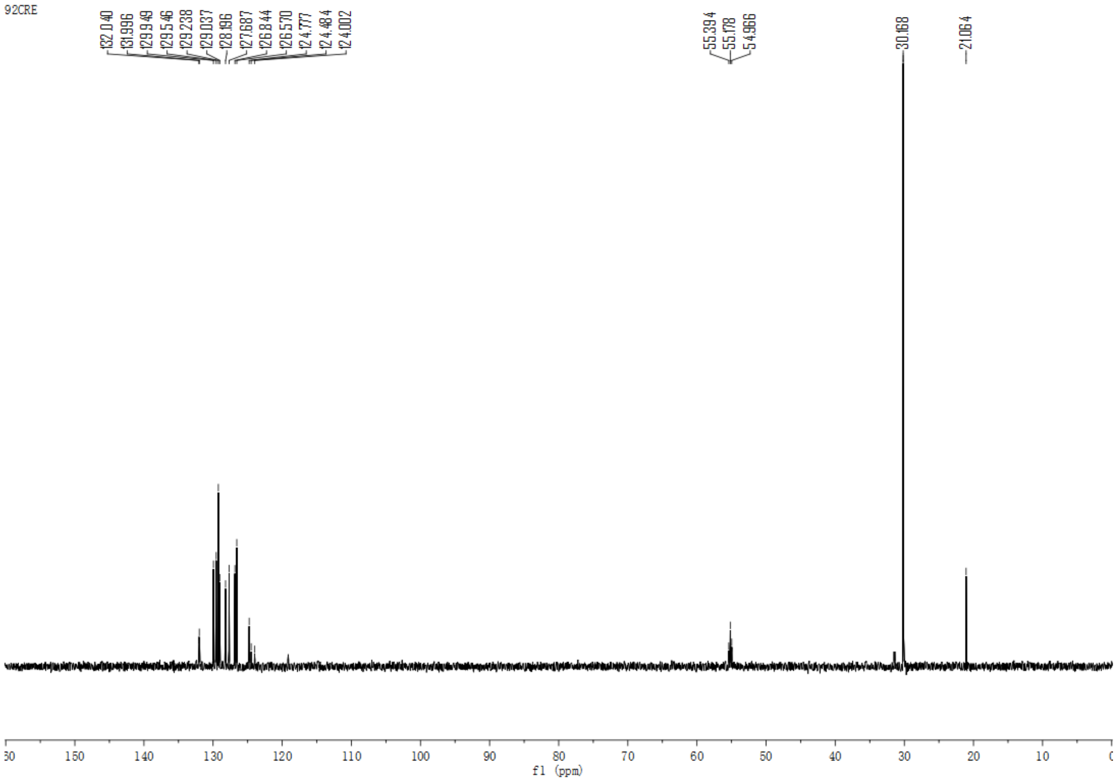

2-(3,5-Di-*tert*-butyl-4-hydroxyphenyl)-1-(1*H*-indol-2-yl)-2-(*p*-tolyl)ethan-1-one (**6**)

^{1}H NMR (400 MHz, CDCl$_3$):

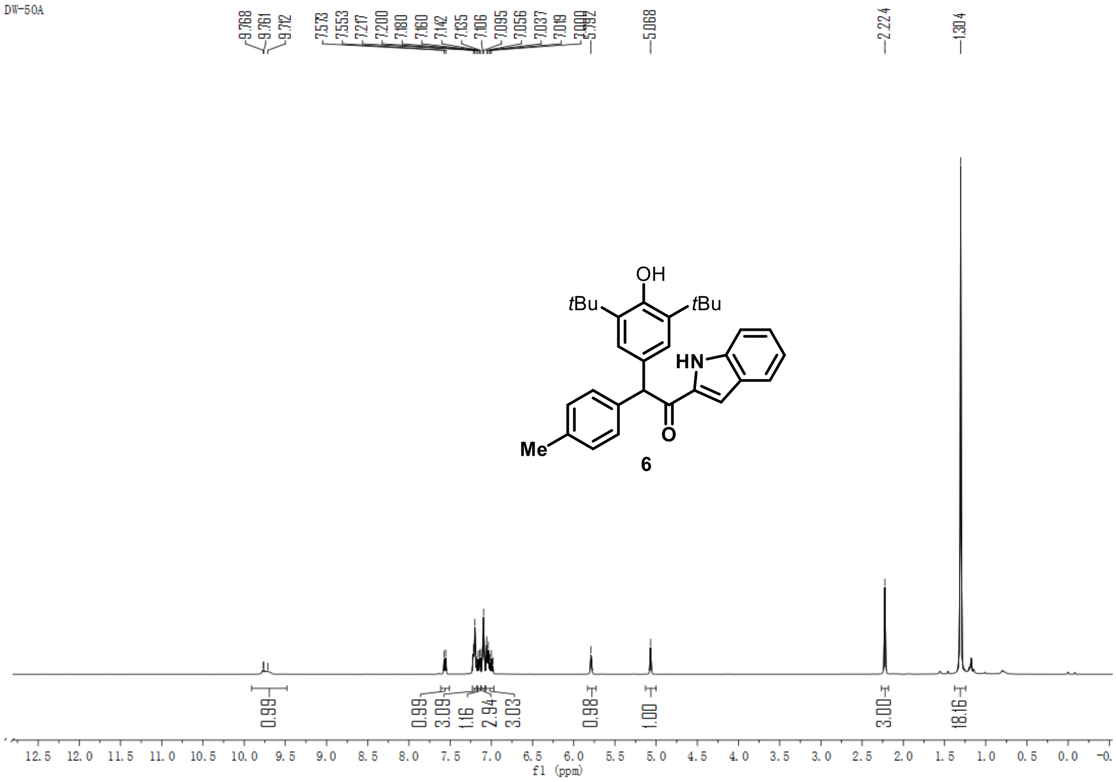

^{13}C NMR (100 MHz, CDCl$_3$):

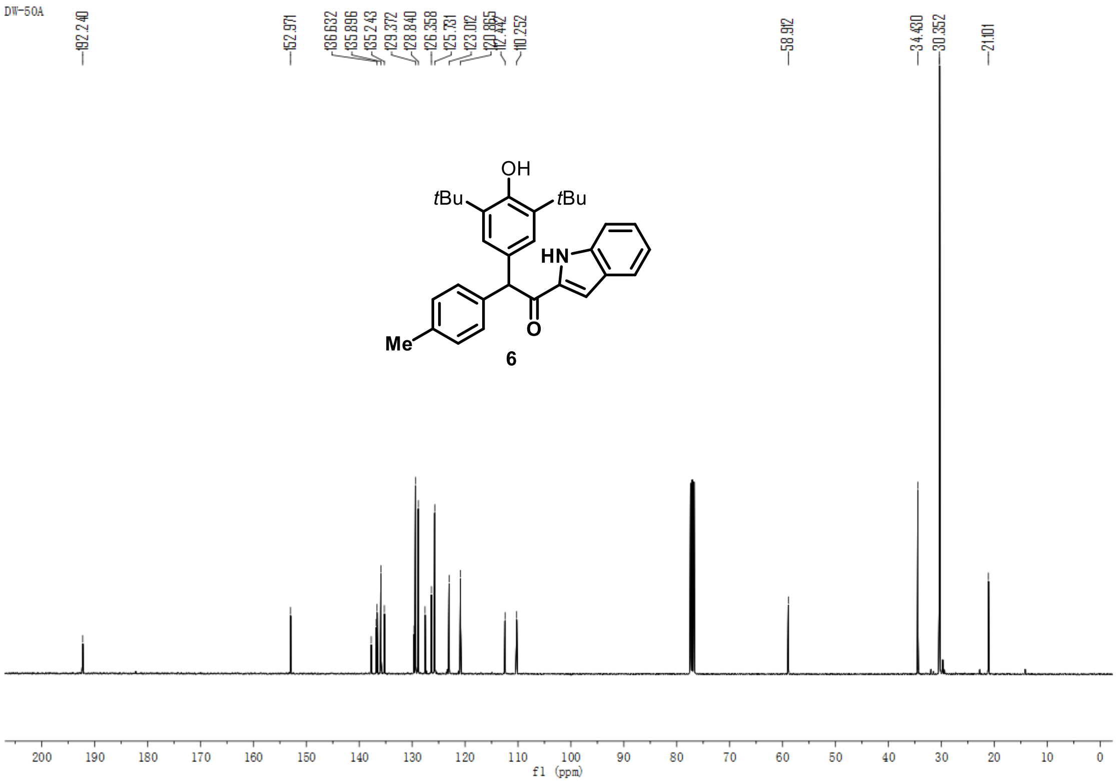

2-(3,5-Di-*tert*-butyl-4-hydroxyphenyl)-1-phenyl-2-(*p*-tolyl)ethan-1-one (**7**)

^{1}H NMR (400 MHz, CDCl$_3$):

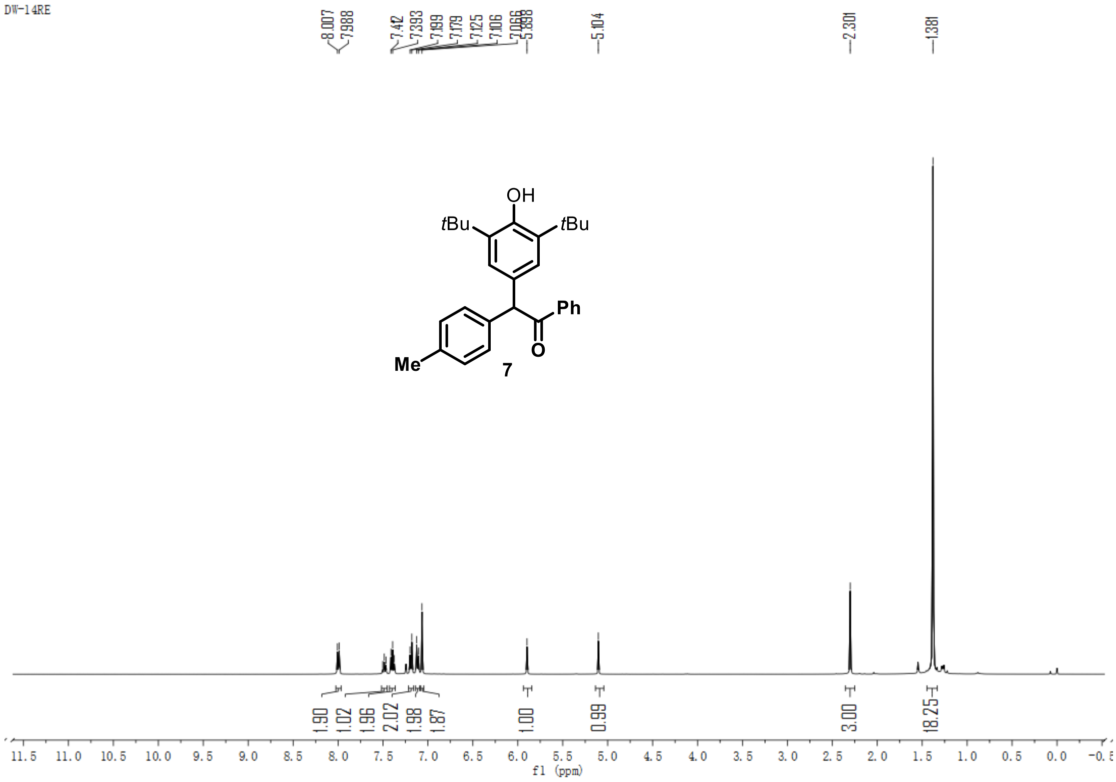

^{13}C NMR (100 MHz, CDCl$_3$):

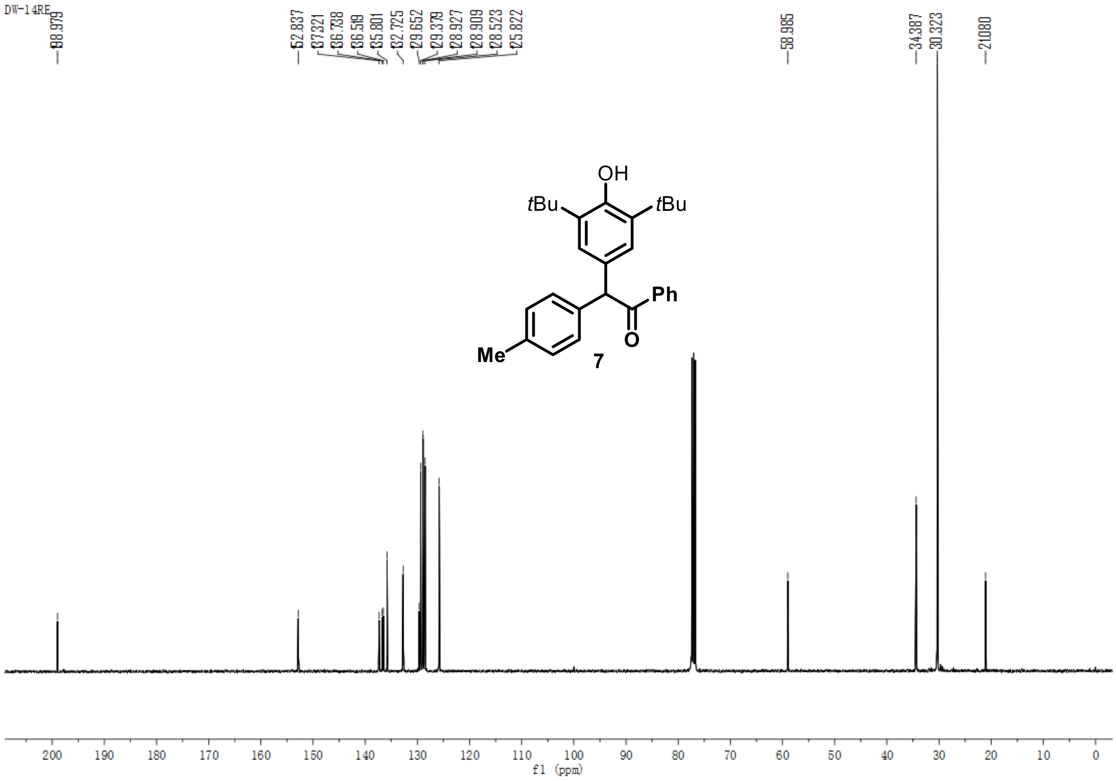

Supplement: File 1 — General information, characterization data, and copies of 1H and 13C NMR spectra. [file Beilstein_J_Org_Chem-17-2822-s001.pdf]
